# Machine learning-based radiomics model: prognostic prediction and mechanism exploration in patients with endometrial cancer

**DOI:** 10.1186/s40364-025-00836-5

**Published:** 2025-09-29

**Authors:** Yu Zhang, Xiaoqing Bao, Yaru Wang, Linrui Li, Long Liu, Qibing Wu

**Affiliations:** 1 Department of Radiation Therapy, The First Affiliated Hospital of Anhui Medical University, Hefei, 230001 Anhui China; 2 Department of Surgery, Perelman School of Medicine, University of Pennsylvania, Philadelphia, Pennsylvania PA 19104 USA; 3 Department of Hepatobiliary and Pancreatic Surgery, The Second Hospital of Zhejiang University, Hangzhou, 310000 Zhejiang China

**Keywords:** Endometrial cancer, Prognosis prediction, Angiogenesis, Radiomics, Machine learning

## Abstract

**Objectives:**

To investigate the predictive value of machine-learning-based Radiomics models for postoperative overall survival (OS) of endometrial cancer (EC) patients and their biological mechanisms.

**Methods:**

Data from 469 patients with endometrial cancer in three Centers (271 in Center 1, 154 in Center 2, and 44 in Center 3) were retrospectively and 90 patients in Center 1 were prospectively analyzed. Three-dimensional Radiomics parameters of the primary lesion and its surrounding 5 mm region in T2WI were collected from all patients. Ten machine learning methods were used to calculate the optimal Radiomics score (Radscore), whose incremental value to the available clinical indexes, pathomics, transcriptomics, and proteomics were revealed. Eventually, TCGA and CPTAC were used for the exploration of biological mechanisms of Radiomics model, with experimental validation.

**Results:**

Radiomics features of tumor and peritumor showed some complementarity in the prognostic prediction of EC patients. The best predictive efficacy was demonstrated by the combined Radiomics model based on XGboost, with AUCs of 0.862, 0.885, 0.870 (validation set) and 0.823, 0.869, 0.849 (test set 1) and 0.850, 0.731, 0.800 (test set 2). Radiomics models demonstrated high incremental value to existing clinical indicators and can effectively improve prognostic prediction. In addition, Radiomics models have been shown to have synergistic prognostic predictive potential with pathomics, transcriptomics, and proteomics. Finally, mechanical explorations suggest that Radiomics models may be associated with tumor angiogenesis-related pathways, of which FLT1 was highlighted.

**Conclusions:**

Machine learning-based Radiomics model contributes to predicting postoperative OS in EC patients and suggests a correlation with tumor angiogenesis.

**Supplementary Information:**

The online version contains supplementary material available at 10.1186/s40364-025-00836-5.

## Keypoints

• Machine-learning-based Radiomics models of tumor and peritumor help in prognostic prediction of EC patients.

• Radiomics has synergistic prognostic potential with clinical markers, pathomics, transcriptomics, and proteomics.

• Radiomics models have value in revealing tumor angiogenesis, blood supply levels, and FLT1 expression.

• IVIM-DWI and DCE-MRI can achieve prognostic prediction of EC patients by revealing angiogenesis.

**Supplementary Information:**

The online version contains supplementary material available at 10.1186/s40364-025-00836-5.

## Introduction

Endometrial cancer (EC) is one of the most common malignant tumors in the female reproductive system, and its incidence is increasing year by year worldwide [1–3]. Comprehensive treatment based on surgery is currently the mainstay of treatment for EC patients [1–5]. Although the five-year survival rate of early-stage EC patients can reach 70–90% after radical surgery, it will drop to about 30% for intermediate-late and recurrent patients [1, 4–6]. Meanwhile, due to the differences in tumor heterogeneity and microenvironment, different patients may show very different efficacy and prognosis after receiving the same treatment [7, 8]. Therefore, accurate prediction of the prognosis of EC patients is of great significance for the development of individualized treatment plans and the improvement of patient survival. Currently, the prognosis prediction of endometrial cancer mainly relies on clinicopathological features, including clinical stage, histological type, degree of differentiation, and lymph node metastasis [2, 9, 10]. However, these traditional prognostic factors cannot fully reflect the biological behavior and progression potential of endometrial cancer. Even The International Federation of Gynecology and Obstetrics (FIGO) staging, which is most commonly used in clinical practice, struggles to meet clinical needs in the prognostic prediction of EC patients (with an accuracy of about 60–70%) [1, 11–13]. Therefore, there is an urgent need to explore more biomarkers to improve the accuracy of prognostic prediction.

Radiomics, as a non-invasive, reproducible, and high-throughput image analysis method, is increasingly used in the diagnosis and treatment of diseases, especially tumors [14]. It can extract a large number of features from medical images, which can reflect biological information about tumors (including morphological, functional, and molecular biological features) and individualized prognostic assessment (providing more precise prognostic information based on the unique characteristics and pathological state of each patient) [14–16]. The combination of Radiomics and machine learning has show promise to facilitate performance optimization and clinical translation of medical image analysis [17]. Radiomics can provide rich image information from which machine learning algorithms can extract valuable features for efficient image classification, segmentation, screening, and prediction [17–19]. In addition, the combination of Radiomics with clinical features and molecular biology features has proved to be promising, which can significantly improve the accuracy, stability, and predictive efficacy of the models [19].

In recent years, multiparametric magnetic resonance imaging (mp-MRI) has played an increasingly important role in studies of tumor function detection and prognostic assessment [20]. Among them, two techniques, Dynamic contrast-enhanced magnetic resonance imaging (DCE-MRI) and Intravoxel incoherent motion diffusion-weighted imaging (IVIM-DWI) have attracted wide attention [20–22]. DCE-MRI can provide information on tumor angiogenesis and permeability by tracking the distribution and clearance process of contrast agents within the tumor [21]. IVIM-DWI allows non-invasive assessment of microvascular structure and hemodynamic characteristics within the tumor [22]. It has shown that DCE-MRI and IVIM-DWI can assess the level of tumor proliferation and vascular status, and predict tumor response to anti-angiogenic therapy and prognosis [20–22]. Meanwhile, with the rapid development of high-throughput sequencing technology and bioinformatics analysis methods, more and more studies have begun to focus on the prognostic impact of molecular features and biological functions of endometrial cancer. For example, polymerase epsilon (POLE) mutation, microsatellite instability (MSI), high hormone levels, and high perfusion are closely associated with the prognosis of endometrial cancer [3, 22].

Therefore, this study aims to clarify the predictive value of machine learning models based on Radiomics features of endometrial cancer and peritumor in the postoperative prognosis of EC patients and their incremental value to existing diagnostic models. It also tries to explore the biological mechanisms of Radiomics prediction of EC and find efficient prognostic molecular markers. This will ultimately provide a more accurate assessment tool for prognostic prediction and individualized treatment of endometrial cancer patients.

## Materials and methods

### Patients

**Fig. 1:**
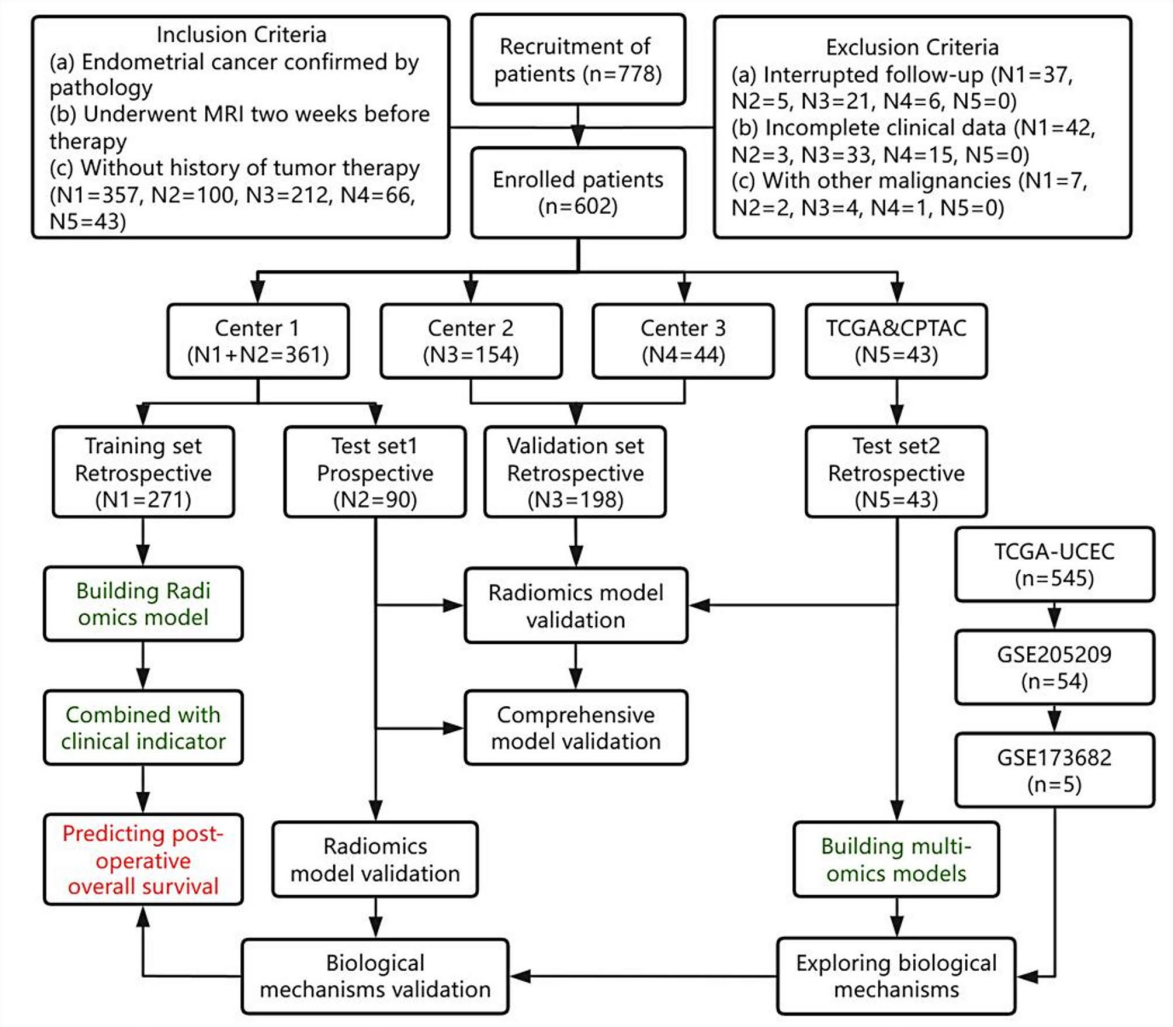
Patient recruitment and study overview

Data of 469 EC patients in Center 1, Center 2, and Center 3 from March 1, 2015 to March 1, 2022 were retrospectively collected. Data of 90 EC patients in Center 1 from 4 March 2020 to 1 May 2024 (test set 1) (registration number ChiCTR2100043892) were prospectively collected. The TCGA-UCEC cohort (8 cases) and the CPTAC-UCEC (35 cases) cohorts were collected from the GDC (test set 2). Based on the inclusion and exclusion criteria finally, 602 patients were included in the study (Fig. 1). All enrolled patients underwent endometrial cancer surgery + lymph node dissection. General clinical data including age, maximum tumor diameter, lymph node metastasis (LNM), and pathological staging were collected from the hospital medical record system. The FIGO stage was reclassified according to the latest guidelines [11]. The study was approved by the ethics committees of the three centers and informed consent for the retrospective study was pardoned (Ethics No: 2024-YXK-08 (Centre 1), PJ2024-03-48 (Centre 2), FNLL202407023 (Centre 3)). Further details of materials and methods are provided in the Supplementary Material. The work has been reported in line with the REMARK criteria [23].

### Protocol

The 3.0T MRI scanner (GE Signa HDXT 3.0T MRI scanner, GE Healthcare, USA) with a phased-array 8-channel sensitivity-encoding abdominal coil was used in Center 1 (Table S1). The 3.0T MRI scanner (Discovery MR750w, General Electric, USA) with an accompanying 8-channel phased-array body coil was used in Center 2. The 1.5T MRI scanner (Avanto, Siemens Healthcare, Erlangen, Germany) with a 16-channel phased array body coil was used in Center 3. All patients had no contraindications to MR examination. Bowel preparation was performed by fasting 8 h before the examination to reduce the influence of bowel contents on the scanning image. Appropriate water was consumed 1 h before the examination to make the bladder full. The scanning range was from the upper edge of the iliac wing to the lower edge of the pubic symphysis.

The surgical protocol was total hysterectomy adnexal resection and pelvic lymph node dissection. Surgical procedures included laparoscopic surgery and open surgery. Follow-up visits were every 3–6 months for 2 years after surgery, every 6–12 months for the 3rd-5th year, and annually thereafter.

### Image

**Fig. 2:**
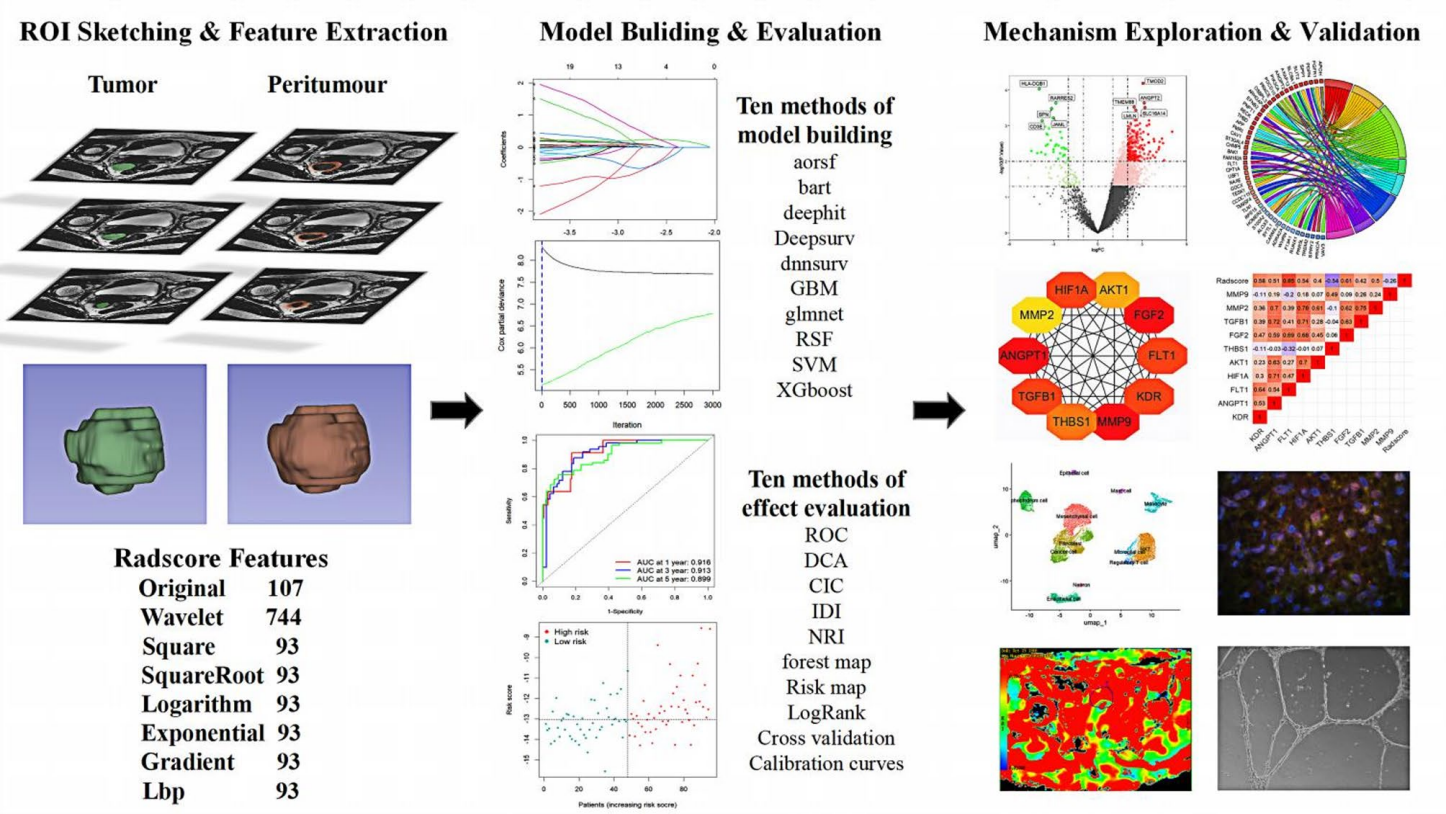
Overview of the study

Radiomics: The region of interest (ROI) of the tumor, which was expanded by 5 mm to extract peritumor Radiomics parameters, on T2WI was outlined layer by layer by two radiologists using 3D slicer. Each level of ROI was fused into a volume of interest (VOI) to extract 3D texture features (Fig. 2). The PyRadiomics was used for Radiomics feature extraction. The robustness of Radiomics features was additionally assessed by two radiologists.

IVIM-DWI: ROIs were manually sketched by two radiologists independently on a GEAW 4.5 workstation. The sequences with b = 800 s/mm^2^ were selected to sketch the tumor ROIs layer by layer, and the parameters of IVIM-DWI were measured: Apparent Diffusion Coefficient (ADC), Diffusion Coefficient (D), Pseudodiffusion Coefficient (D*) and Perfusion Fraction (f).

DCE-MRI: Tumor ROIs (selecting the largest level of the tumor) were manually outlined by 2 radiologists using GE Omni Kinetic. The DCE-MRI parameters: Transfer Constant (Ktrans), rate constant (Kep), Extravascular Extracellular Volume (Ve), and capillary plasma volume (Vp) were derived from the TKmodel. IVIM- DWI and DCE-MRI were used for quantitative assessment of the level of tumor angiogenesis and blood supply.

### Construction of radiomics model

Spearman’s correlation analysis was performed on Radiomics parameters with interclass correlation coefficient (ICC) greater than 0.75 both within and between groups, where redundant parameters greater than 0.90 were eliminated. The least absolute shrinkage and selection operator (LASSO) was used for feature selection. Subsequently, 10 machine learning methods (aorsf, bart, dephit, Deepsurv, dnnsurv, GBM, glmnet, RSF, SVM, XGboost) were used in the constructed models, where the model with the best predictive efficacy was named as the best Radiomics score (Radscore). This study adhered to CLEAR for reporting and its quality was assessed using RQS and METRICS.

### Combination of radiomics and clinical information

Spearman correlation analysis was used to exclude covariate parameters before combining multiple clinical indicators with Radscore. A comprehensive nomogram was constructed based on independent predictors from multifactorial Cox regression analyses. The incremental value of the Radiomics model for clinical parameters was also analyzed using the net reclassification index (NRI) and integrated discrimination improvement (IDI).

### Radiomics in association with pathomics, transcriptomics and proteomics

Radiomics (Radiomics involves high-throughput extraction of quantitative features from medical images to decode tumor pathophysiology), Pathomics (Pathomics extracts histopathological features from H&E-stained images using computational tools), Transcriptomics (Transcriptomics analyzes expression levels of RNA molecules to identify tumor-associated gene signatures), and Proteomics (Proteomics quantifies tumor-related protein expression profiles) parameters were collected from 35 patients of CPTAC-UCEC. The LASSO model was used to construct the Pathomics, Transcriptomics, and Proteomics models. Radiomics models were combined with Pathomics, Transcriptomics, and Proteomics models to analyze their complementary value in the prognostic prediction of EC patients.

### Search for biological mechanisms in radiomics models

Thirty-five patients from CPTAC-UCEC were used for the analysis of the biological mechanisms of the Radiomics model. Patients were stratified according to the Radiomics score (cut-off values were derived from the surv_cutpoint function). Intersecting differential genes in the Transcriptome and Proteome were derived and enriched. The cytoscope was used to find hub genes in the intersecting genes. The Spearman was used to analyze the correlation of hub genes with Radscore. TCGA-UCEC (545 EC patients), GSE205209 (54 EC patients), and GSE173682 (5 single-cell EC samples) were used for hub gene validation.

### Validation of biological mechanisms

IVIM-DWI and DCE-MRI were used for quantitative assessment of tumor angiogenesis and blood supply levels. Cell co-culture, angiogenesis assay, cck8 assay, Transwell assay, scratch assay, cytoskeleton staining, plate cloning assay, and tissue immunofluorescence were used for functional validation of hub genes.

### Statistical analyses

The R4.4.0 was used to perform statistical analyses: ICC was used to test the robustness of Radiomics parameters. The LASSO was used for Radiomics feature selection. Aorsf, bart, dephit, deepsurv, dnnsurv, gbm, glmnet, rsf, svm, xgboost were used for Radiomics model construction. The log-rank test was used for univariate analysis. Multivariate Cox regression analyses were used for nomogram construction. The time-dependent ROC curves, calibration curves, decision curves, clinical impact curves, NRI, and IDI were used to assess the predictive efficacy and value of the combined model.

## Results

### Clinical data

A total of 602 patients were included in this study, 271 (retrospectively) & 90 (prospectively) in Center 1, 154 in Center 2, 43 in Center 3, 8 in TCGA-UCEC, and 35 in CPTAC-UCEC. Median age 56 years, range 27–89 years. 192 deaths (31.3%). Test set 2 was not included in the clinical model validation due to some missing clinical data (Table 1). There were no statistically significant differences in clinical data between the training and validation sets (Table S2). Further details of the result are provided in the Supplementary Material.

**Table 1:** Baseline data table of 602 patients with endometrial cancer

Parameters	Center 1 Training set (*n* = 271)	Center 2 Validation set (*n* = 154)	Center 3 Validation set (*n* = 44)	Center 1 Test set1 (*n* = 90)	TCGA&CPTAC Test set2 (*n* = 43)
Death	92 (33.9%)	53 (34.4%)	13 (29.5%)	25 (27.8%)	9 (20.9%)
Age	56 [52, 61]	57 [55, 64]	56 [52, 61]	55 [51, 58]	65 [59, 70]
Height (m)	1.58 ± 0.36	1.55 ± 0.30	1.56 ± 0.32	1.58 ± 0.35	/
Weight (kg)	61.6 ± 8.51	63.4 ± 8.91	60.2 ± 8.03	61.4 ± 8.20	/
Body mass index	25.22 ± 6.02	26.03 ± 6.74	25.46 ± 6.21	25.27 ± 6.46	/
Age of menarche	15 [14, 16]	15 [14, 16]	15 [14, 15]	15 [14, 16]	/
Menopausal	180 (66.4%)	110 (71.4%)	31 (72.1%)	55 (61.1%)	/
Max diameter (cm)	3.56 ± 1.16	3.34 ± 1.04	3.62 ± 1.30	3.47 ± 1.06	/
CA125(U/mL)	42.83 ± 6.77	47.46 ± 6.92	41.01 ± 5.06	43.58 ± 6.43	/
Lymph node metastasis	17 (6.3%)	6(3.9%)	2(4.5%)	6 (6.7%)	7 (16.2%)
Deep myometrial infiltration	113 (41.7%)	63 (40.9%)	19 (43.2%)	41 (45.6%)	/
Differentiation					/
G1	94 (34.7%)	48 (31.2%)	14 (31.8%)	28 (31.1%)	
G2	106 (39.1%)	59 (38.3%)	18 (40.9%)	32 (35.6%)	
G3	71 (26.2%)	47 (30.5%)	12 (27.3%)	30 (33.3%)	
Clinical types					/
type I	177 (65.3%)	102 (66.2%)	30 (68.2%)	57 (63.3%)	
type II	94 (34.7%)	52 (33.8%)	14 (31.8%)	33 (36.7%)	
Histological type					
Endometrial Adenocarcinoma	223 (82.3%)	134(87.0%)	37(84.1%)	74 (82.2%)	40 (93.0%)
Plasma Cancer	36 (13.3%)	13(8.4%)	5(11.6%)	12 (13.3%)	3 (7.0%)
Clear Cell Adenocarcinoma	5 (1.8%)	3(1.9%)	0(0.0%)	1 (1.1%)	0 (0.0%)
Other types	7 (2.6%)	4(2.6%)	2(4.7%)	3 (3.3%)	0 (0.0%)
FIGO stage					
I	213 (78.6%)	124 (80.5%)	36 (83.7%)	68 (75.6%)	28 (65.1%)
II	33 (12.2%)	22 (14.3%)	5 (11.4%)	14 (15.6%)	5 (11.6%)
III	19 (7.0%)	7 (4.5%)	2 (4.7%)	7 (7.8%)	7 (16.3%)
IV	6 (2.2%)	1 (0.6%)	1 (2.3%)	1 (1.1%)	3 (7.0%)

### Radiomics model construction

**Fig. 3:**
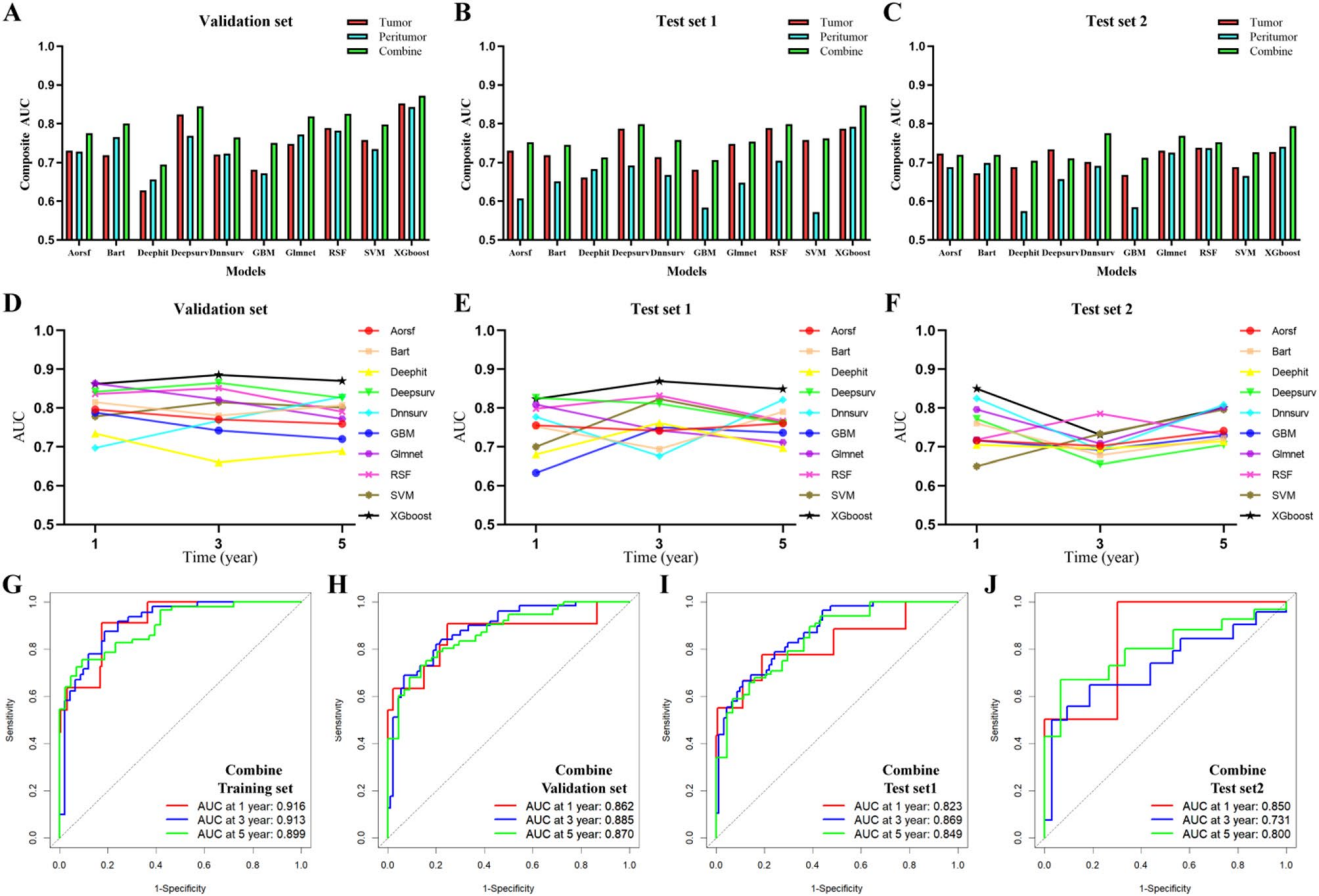
Comparison of predictive efficacy of Radiomics models based on machine learning. Figures **A**, **B**, and **C** show the comparison of the Composite AUC of Radiomics models for predicting the prognosis of EC patients in the validation set, test set 1, and test set 2, which indicates that the combined model with tumor and peritumor has a higher prediction efficacy; Figures **D**, **E** and **F** show the comparison of the AUC of the combined model for predicting the prognosis of EC patients in the validation set, test set 1 and test set 2; Figures **G**, **H**, **I**, and **J** show the ROC curves of the combined tumor and peritumor-based Radiomics model in the training set, validation set, test set 1 and test set 2. The results show that the combined model based on XGboost has the best prediction efficacy. Composite AUC = (1-year AUC + 3-year AUC + 5-year AUC)/3*100%

A total of 16 tumor Radiomics features and 12 peritumor Radiomics features were used for machine learning modeling (Table S3&S4). Among the 10 machine learning models, the XGboost-based Radiomics model showed the best predictive efficacy (Fig. 3&S1). The Radiomics features of tumor and peritumor were complementary to the prognostic prediction of EC patients. The AUCs of the combined model for predicting 1-, 3-, and 5-year OS were 0.916, 0.913, and 0.899 (training set) and 0.862, 0.885, and 0.870 (validation set), and 0.823, 0.869, and 0.849 (test set 1) and 0.850, 0.731, and 0.800 (test set 2), respectively (Table 2, Figure S2). The CLEAR was used for the evaluation of Radiomics studies (Table S5). The RQS and METRICS were used to assess the research methodology, where the RQS score was 31 out of 36 (Table S6) and the METRICS score was 93.1% (Table S7). The DeLong test for XGBoost and other models is shown in Table S8.

**Table 2:** Comparison of the effectiveness of different machine learning models

Group		AUC	Aorsf	Bart	Deephit	Deepsurv	Dnnsurv	GBM	Glmnet	RSF	SVM	XGboost
Tumor	Training set	1-year	0.944	0.926	0.981	0.991	0.987	0.984	0.967	0.906	0.872	0.900
		3-year	0.931	0.896	0.990	0.982	0.994	0.935	0.948	0.911	0.844	0.916
		5-year	0.890	0.903	0.949	0.969	0.970	0.970	0.926	0.924	0.870	0.873
	Validation set	1-year	0.784	0.755	0.601	0.855	0.738	0.619	0.815	0.806	0.732	0.849
		3-year	0.711	0.654	0.679	0.821	0.603	0.734	0.733	0.811	0.790	0.879
		5-year	0.696	0.748	0.603	0.796	0.821	0.691	0.696	0.749	0.753	0.829
	Test set 1	1-year	0.784	0.755	0.601	0.815	0.738	0.619	0.815	0.806	0.732	0.841
		3-year	0.711	0.654	0.779	0.801	0.603	0.734	0.733	0.811	0.790	0.810
		5-year	0.696	0.748	0.603	0.746	0.801	0.691	0.696	0.749	0.753	0.711
	Test set 2	1-year	0.694	0.655	0.658	0.783	0.624	0.685	0.755	0.788	0.705	0.811
		3-year	0.781	0.659	0.626	0.716	0.690	0.627	0.741	0.726	0.657	0.749
		5-year	0.693	0.701	0.781	0.702	0.790	0.693	0.696	0.699	0.702	0.622
Peritumor	Training set	1-year	0.911	0.896	0.998	0.990	0.958	0.891	0.955	0.943	0.875	0.928
		3-year	0.943	0.913	0.989	0.981	0.915	0.908	0.908	0.916	0.892	0.812
		5-year	0.856	0.881	0.881	0.943	0.966	0.889	0.916	0.896	0.796	0.839
	Validation set	1-year	0.744	0.784	0.708	0.782	0.773	0.639	0.795	0.790	0.744	0.863
		3-year	0.739	0.751	0.590	0.743	0.793	0.694	0.800	0.818	0.763	0.873
		5-year	0.701	0.760	0.671	0.781	0.603	0.684	0.721	0.739	0.696	0.793
	Test set 1	1-year	0.691	0.582	0.696	0.644	0.763	0.555	0.632	0.715	0.533	0.765
		3-year	0.533	0.673	0.744	0.731	0.628	0.586	0.711	0.698	0.569	0.781
		5-year	0.596	0.699	0.610	0.702	0.614	0.610	0.602	0.700	0.613	0.831
	Test set 2	1-year	0.674	0.742	0.638	0.591	0.765	0.647	0.701	0.752	0.622	0.785
		3-year	0.691	0.663	0.576	0.666	0.636	0.535	0.698	0.716	0.684	0.756
		5-year	0.700	0.691	0.508	0.713	0.672	0.571	0.778	0.744	0.690	0.680
Combine	Training set	1-year	0.966	0.956	0.985	0.998	0.984	0.997	0.982	0.955	0.912	0.916
		3-year	0.972	0.936	0.995	0.987	0.990	0.983	0.996	0.962	0.946	0.913
		5-year	0.926	0.904	0.956	0.980	0.962	0.991	0.967	0.960	0.881	0.899
	Validation set	1-year	0.796	0.815	0.734	0.842	0.697	0.788	0.863	0.836	0.778	0.862
		3-year	0.770	0.780	0.660	0.865	0.767	0.742	0.821	0.851	0.815	0.885
		5-year	0.759	0.807	0.689	0.826	0.829	0.720	0.772	0.790	0.801	0.870
	Test set 1	1-year	0.755	0.752	0.680	0.826	0.777	0.633	0.809	0.798	0.700	0.823
		3-year	0.741	0.694	0.761	0.811	0.676	0.750	0.742	0.832	0.823	0.869
		5-year	0.760	0.790	0.697	0.760	0.821	0.736	0.711	0.767	0.762	0.849
	Test set 2	1-year	0.716	0.760	0.705	0.772	0.825	0.716	0.796	0.718	0.650	0.850
		3-year	0.702	0.678	0.695	0.655	0.692	0.692	0.708	0.806	0.733	0.731
		5-year	0.741	0.722	0.714	0.705	0.808	0.729	0.802	0.731	0.796	0.800

### Incremental value of radiomics model

**Fig. 4:**
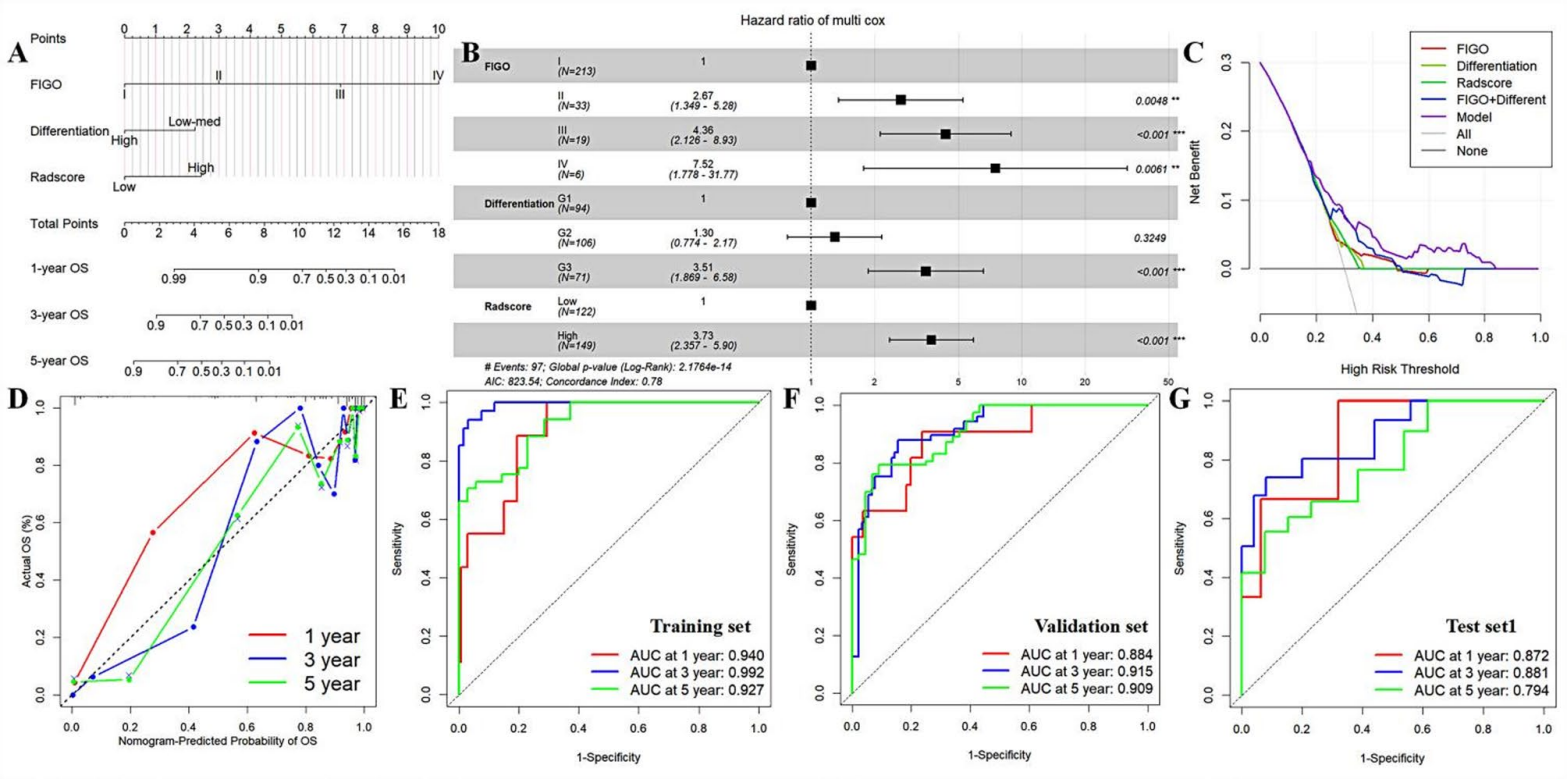
Incremental value of the Radiomics model for clinical indicators. Figure **A** shows the nomogram of the Radiomics model in conjunction with the clinical parameters; Figure **B** shows the forest plot of the OR values of each parameter in the nomogram; Figures **C**, **D**, and **E** show the calibration curves, decision curves, and clinical impact curves of the nomogram, suggesting that the model has a high degree of goodness of fit and clinical gain; Figures **F**, **G**, and **H** show the ROC curves of the nomogram in the training, validation, and test sets, demonstrating that the model is accurate and efficient, stable

Among the clinical indicators, FIGO stage, LNM, differentiation level, and Clinical types were significantly associated with postoperative survival in EC patients. Multivariate Cox regression analysis showed that FIGO stage, differentiation level, and Radscore were independent prognostic factors in EC patients (Fig. 4). The AUCs of the combined model were 0.940, 0.992, and 0.927 (training set) 0.884, 0.915, and 0.909 (validation set), and 0.872, 0.881, and 0.794 (test set 1), respectively. The NRI and the IDI suggested that the overall predictive efficacy of the model was significantly improved by the addition of Radscore (Figure S3, Table S9).

### The value of multi-omics association

**Fig. 5:**
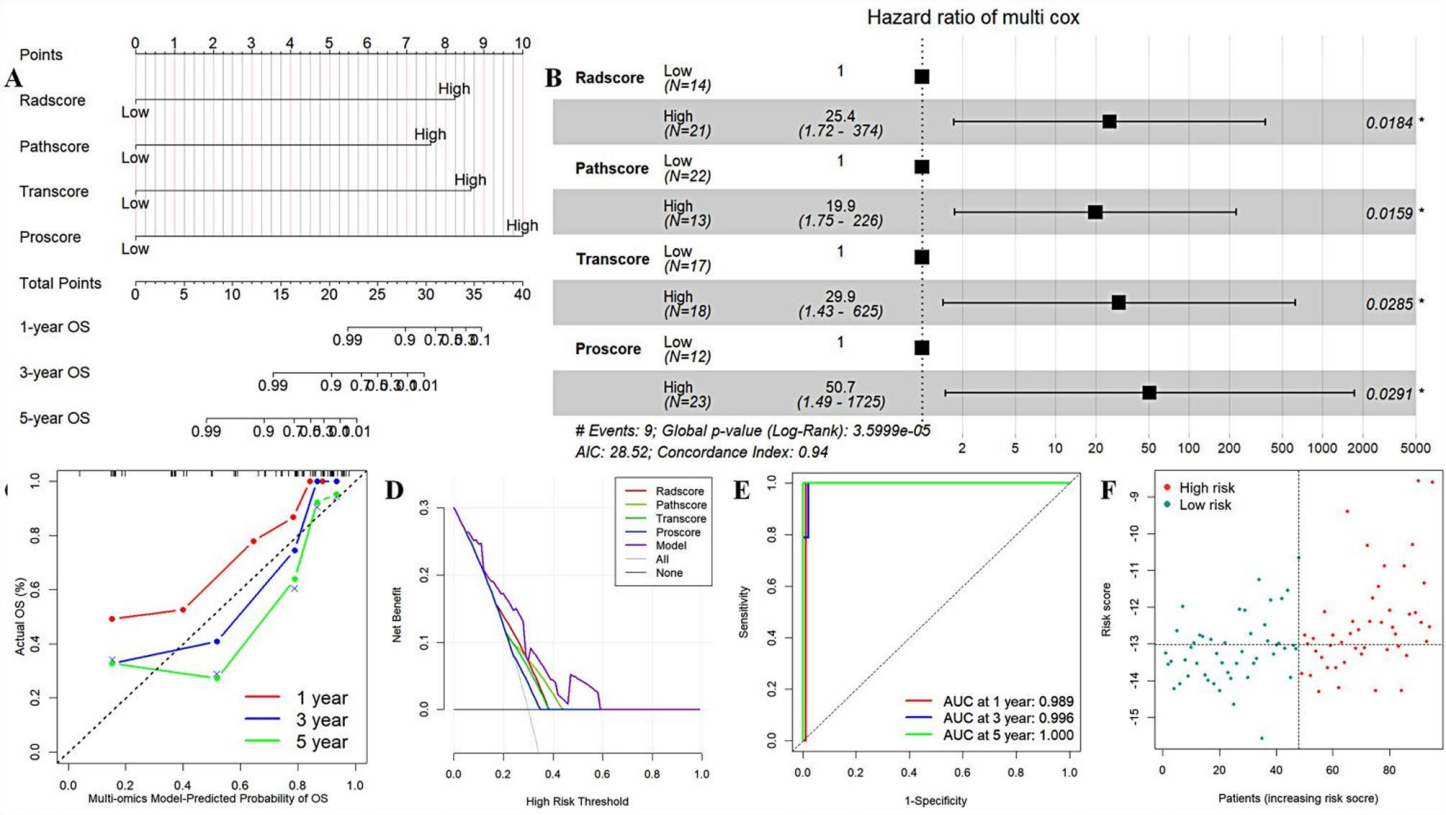
Joint value of multi-omics models. Figure **A** shows the nomogram of the combined Radiomics, Pathomics, Transcriptomics, and Proteomics; Figure **B** shows the forest plot of the combined model; Figures **C**, **D**, and **E** show the calibration curves, decision curves, and ROC curves of the combined model, suggesting that the model has a high level of goodness of fit and clinical benefit; Figure **F** shows the risk distribution plot of the combined model, demonstrating that the model can accurately stratify endometrial cancer patients

Radiomics, Pathomics, Transcriptomics, and Proteomics models were constructed for CPTAC-UCEC patients (Figure S4). The combined model based on the multi-omics models demonstrated strong joint value and predictive efficacy, with 1-, 3-, and 5-year AUCs of 0.989, 0.996, and 1.000 (Fig. 5&Table S10). The calibration curve indicated that the model fit well (Figure S5&Table S11). The model can accurately stratify EC patients according to prognostic risk.

### Biological mechanisms of radiomics model

**Fig. 6:**
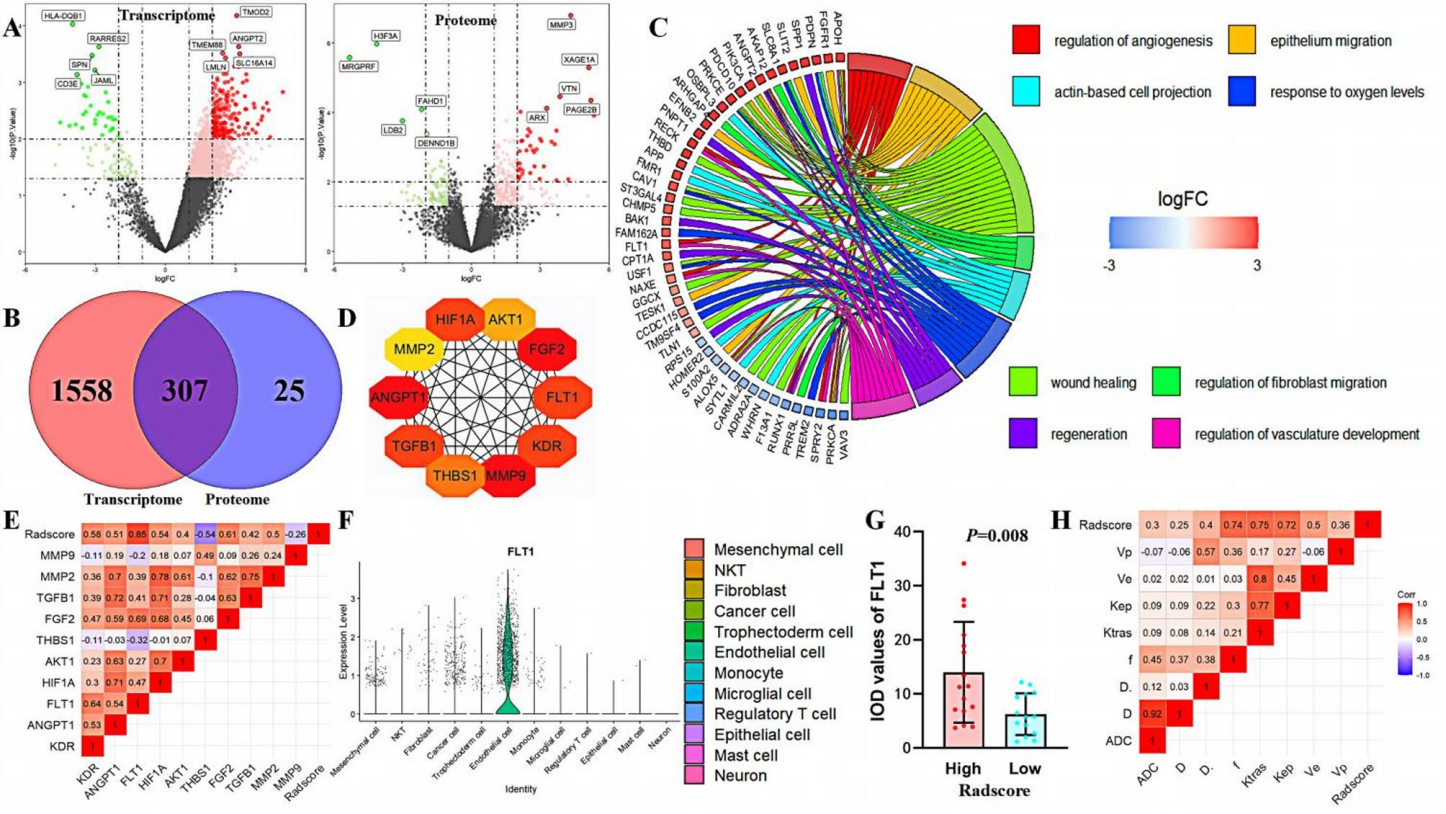
Biological mechanisms of Radiomics model. Figure **A** shows the volcano map of transcriptomic and proteomic differential genes stratified according to Radscore; Figure **B** shows the intersection of transcriptomic and proteomic differential genes, suggesting that there are 307 intersecting genes; Figure **C** shows the GO & KEGG enrichment analysis of transcriptomic and proteomic intersecting differential genes, with the functions mainly enriched in the angiogenesis- and hypoxia-response-associated pathways; Figure **D** shows the cytoscope selection of the top10 hub genes among the intersecting genes; Figure **E** shows the correlation heatmap between hub genes and Radscore, in which FLT1 was most correlated with Radscore (*r* = 0.73, *P* < 0.05); Figure **F** shows the single-cell analysis, suggesting that FLT1 was expressed predominantly in endometrial carcinoma endothelial cells, followed by tumor cells; Figure **G** shows the correlation of FLT1 in the high and low Radscore patients, suggesting that FLT1 was highly expressed in EC patients with high Radscore; Figure **H** shows the correlation between Radiomics scores and quantitative parameters of IVIM-DWI and DCE-MRI (reflecting angiogenesis), suggesting that the Radiomics model was associated with endometrial cancer angiogenesis

Patients in CPTAC-UCEC were grouped according to Radscore (cut-off value of 1.690). The transcriptome had 1865 differential genes (1693 up-regulated, 172 down-regulated), functionally enriched for epithelial cell proliferation and vasculature system regulation (Figure S6). The proteome had 332 differential genes (up-regulated 244, down-regulated 88) with functional enrichment in endothelial cell migration and hypoxia-responsive pathways. The transcriptome and proteome intersected a total of 307 genes, functionally enriched in angiogenesis and hypoxia response (Fig. 6). The top 10 hub genes were clarified by Cytoscape, of which FLT1 was significantly correlated with Radscore (*r* = 0.85, *P* = 0.001, q = 0.008 corrected by the Benjamini-Hochberg) and prognosis (Figure S7).

### Validation of biological function

**Fig. 7:**
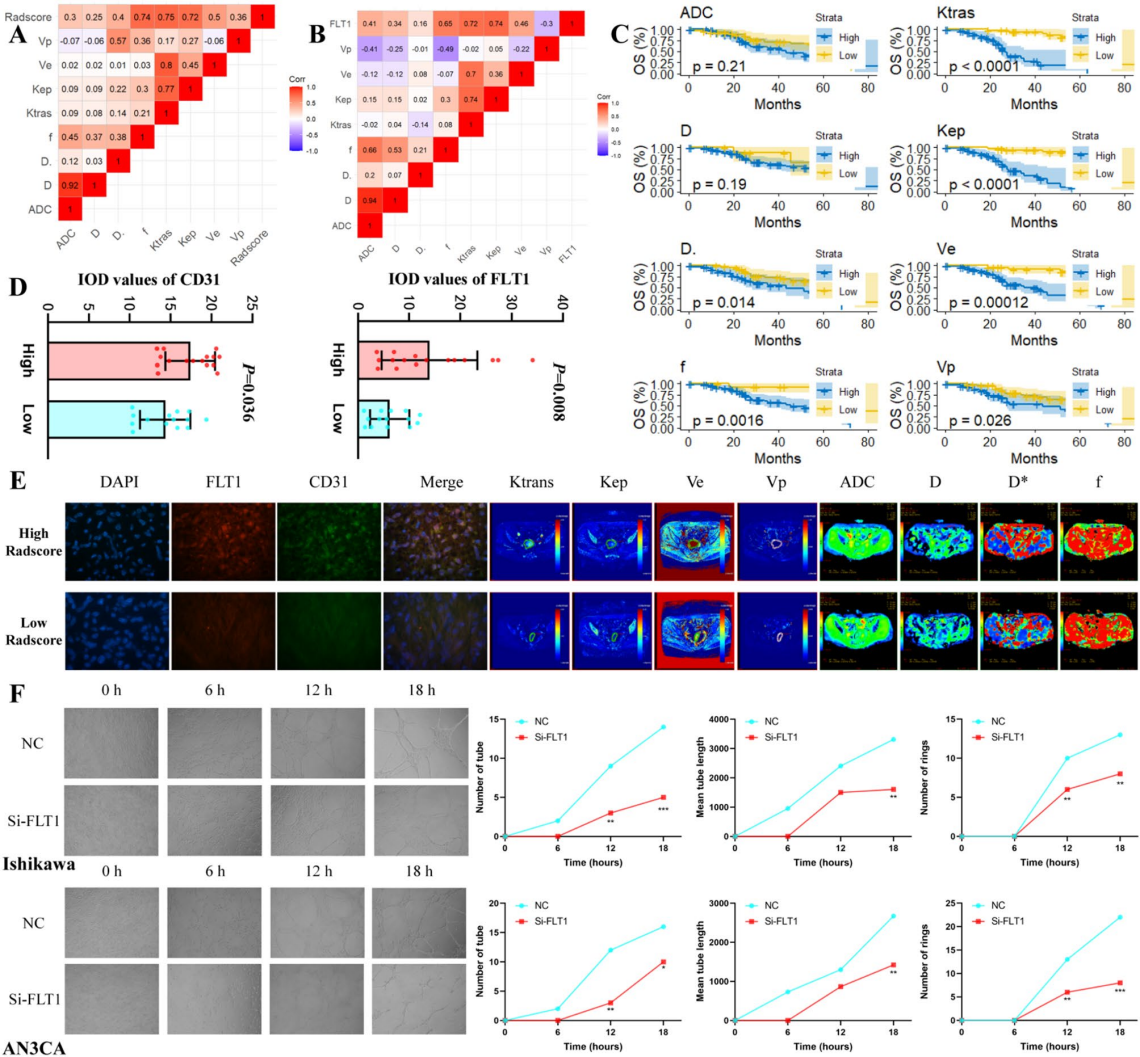
Validation of biological mechanisms. Figure **A** shows the correlation heatmap of IVIM-DWI and DCE-MRI parameters with Radscore, suggesting that f-value, Ktrans, and Kep were significantly correlated with Radscore; Figure **B** shows the correlation heatmap of IVIM-DWI and DCE-MRI parameters with FLT1, suggesting that f-value, Ktrans, and Kep were significantly correlated with FLT1; Figure **C** shows the prognostic value of IVIM-DWI and DCE-MRI parameters in endometrial cancer patients, suggesting that IVIM-DWI and DCE-MRI have the potential to predict the prognosis of EC patients; Figure **D** shows the immunofluorescence of FLT1 and CD31 in 30 endometrial cancer patients with high and low Radscore, suggesting that high Radscore patients have higher levels of FLT1 and CD31 expression; Figure **E** shows typical examples of FLT1, CD31 and IVIM-DWI and DCE-MRI parameters in high and low Radscore patients; Figure **F** shows the angiogenesis assay, where HUVEC were co-cultured with FLT1 knocked down AN3CA/Ishikawa

Retrospective analyses suggested that the Radiomics model may be associated with the level of angiogenesis and blood supply in endometrial cancer. Therefore, we prospectively collected IVIM-DWI and DCE-MRI parameters from 90 patients to reveal the association between the Radscore and angiogenesis. The results showed that the f values of IVIM-DWI, as well as the Ktrans and Kep values of DCE-MRI, were significantly correlated with Radscore, which suggests that patients with a high Radscore tend to have a more robust blood supply and angiogenesis (Fig. 7&Table S12). Meanwhile, the prognostic value of IVIM-DWI and DCE-MRI parameters for endometrial cancer was also revealed. The correlation of FLT1 and Radscore with IVIM-DWI and DCE-MRI was also demonstrated.

### Validation of molecular mechanisms

The endometrial cancer cell lines Ishikawa and AN3CA were used to knock down FLT1 expression by siRNA (Figure S8&S9). The results showed that knockdown of FLT1 inhibited endometrial cancer cell migration, invasion, proliferation, and angiogenesis (after co-culture).

## Discussion

In this study, machine learning-based Radiomics parameters demonstrated the ability to predict postoperative overall survival in endometrial cancer patients, where tumor and peritumor Radiomics features showed a high degree of complementarity. The simultaneous application of multiple machine learning and deep learning provides more potential for optimal screening and combination of Radiomics parameters. Meanwhile, the Radiomics model not only demonstrates incremental value to the existing prediction model (clinical predictors based on the FIGO stage), but also shows high complementary potential in association with Pathomics, Transcriptomics, and Proteomics. The addition of Radiomics has significantly improved the predictive efficacy of the model. The combined model can assist in the early clinical stratification of EC patients, thereby assisting in clinical decision-making and improving clinical outcomes. In addition, we have built a bridge between Radiomics and genomics and explored the possible biological mechanisms of the Radiomics model. Transcriptomics and Proteomics analyses suggested that the Radiomics model established in this study has the value of revealing the level of angiogenesis and blood supply in endometrial cancer, and is highly correlated with the expression of FLT1, which was proved by prospective studies and experimental results.

Radiomics is a promising high-throughput method to quantitatively characterize textural differences in imaging at the microscopic level and from a structural point of view, thereby generating new imaging biomarkers [24, 25]. In this study, three-dimensional texture features of the primary lesion and 5 mm of the peritumor were extracted to non-invasively achieve a “virtual biopsy” of the tumor and subclinical lesions [25, 26]. Moreover, the Radiomic features of tumor and peritumor showed high complementarity in the prediction of postoperative survival of EC patients. This suggests that peritumor radiomics parameters of endometrial cancer also contain important biological information, which can effectively compensate for the limited information about the tumor. Meanwhile, advances in artificial intelligence have made it possible to accurately screen and efficiently combine the vast amount of Radiomics features, providing a broader space for Radiomics applications [18]. In this study, ten machine learning and deep learning methods were employed to select the best possible combination model of radiomics parameters and enhance its clinical application value. The results show that the XGboost model has the best predictive efficacy (although the performance in the training set is not optimal, it performs well in both the validation set and the test set), which can provide reliable support for prognostic prediction and clinical decision-making in EC patients. The model enables risk stratification: high-risk patients may benefit from intensified adjuvant therapy and closer surveillance, while de-escalation could be considered for low-intermediate risk cases.

To further analyze the compatibility, complementarity, and incremental value of Radiomics with existing diagnostic and treatment modalities. We combined multiple common clinical prognostic indicators with Radscore. The results showed that FIGO stage, degree of differentiation, and Radscore have independent predictive value in the prognostic prediction of EC patients. FIGO stage, as the most frequently used clinical indicator in clinical diagnosis and treatment, can reveal the depth and extent of the tumor invasion and has been demonstrated to potentially correlate with tumor malignant potential, immune status, genotypic phenotypes, and drug resistance, which undoubtedly has a high prognostic value [12, 27, 28]. Differentiation has the value of revealing the tumor growth and proliferation phenotype and recurrence and metastatic potential, which is significantly correlated with the prognosis of endometrial cancer [29]. The combination of FIGO stage, differentiation, and Radscore complement each other, revealing the prognostic level of patients with endometrial cancer in terms of macroscopic clinical, microgenetic, and epigenetic imaging, respectively. It is exciting to note the complementary nature of radiomics not only with existing clinical indicators, but also with Pathomics, Transcriptomics, and Proteomics. The multi-omics-based model will provide a more comprehensive predictive perspective for the prognostic prediction of EC patients.

Radiomics is data-driven in nature, and the underlying biological significance has not yet been clarified, which is important for understanding disease development, exploring novel diagnostic and treatment modalities, and promoting the clinical application of models [30, 31]. Unlike previous studies of endometrial cancer prognostic models [32–34], in this study, we correlated radiomics with Transcriptomics and Proteomics to find the biological function (angiogenesis) and possible molecular mechanism (FLT1) behind the Radiomics model. Moreover, compared to other studies that correlate radiomics with genes [30, 35], this study additionally validates the function and mechanism of the Radiomics model in a prospective cohort. The results demonstrated that the prognostic capability of the XGboost-based radiomics model was achieved in part by revealing the level of blood supply in endometrial cancer, which was closely associated with FLT1 expression. This not only can provide objective and effective support for the diagnosis and treatment of endometrial cancer but also implies that the assessment of tumor biological functions and gene expression levels may be achieved by non-invasive means in the future.

In addition, in the validation of the function and mechanism of the radiomics model, we also found that IVIM-DWI and DCE-MRI, which are used to reveal the level of tumor blood supply and vascular permeability [36, 37], also have the potential to predict the prognosis of endometrial cancer. The f (mainly reflecting the level of overall blood supply) and D* (mainly reflecting the velocity of blood flow) in IVIM-DWI and Ktrans (mainly reflecting vascular permeability and plasma flow), Kep (mainly reflecting vascular permeability), Vp (mainly reflecting vascular density), and Ve (mainly reflecting extravascular-extracellular interstitial volume) in DCE-MRI demonstrated predictive value for postoperative survival in EC patients [36–38]. This not only reaffirms the value of blood supply level in the prognosis of EC patients, whose prognostic value has been demonstrated in a variety of tumors [36–41] but also provides new ideas and tools for the prediction of the prognosis of EC patients. In addition, the endometrium has a unique steroidal vasoregulatory mechanism and cyclic growth pattern, which may lay the groundwork for abnormal vascular growth and tumor regulation [41–43]. For Radiomic high-angiogenic features, prioritized anti-angiogenic therapy warrants investigation.

There are also some limitations in this study. Firstly, although manual outlining of tumor ROIs is still the most accurate method available, it lacks reproducibility and objectivity. Consequently, it is necessary to improve the application of artificial intelligence in target areas outlined in the future. Secondly, multi-omics studies are limited by sample size and lack external clinical cohort validation, resulting in insufficient evidence of the model’s clinical utility. In the future, we will analyze the clinical value of multi-omics models in larger prospective studies. In addition, the single-sequence selection (T2WI) and 5 mm tumor margin setting in this study were based on initial exploratory objectives and clinical rationality, but may be limited by insufficient information. Eventually, there is an element of sex hormone-dependent angiogenesis in endometrial cancer. Therefore, in the future, it is necessary to analyze endometrial cancer patients with differences in ER/PR expression independently, to clarify more clearly the prognostic value of angiogenesis and blood supply levels in endometrial cancer patients.

## Conclusion

In conclusion, Radiomics features of tumors and peritumors can to some extent reveal the level of angiogenesis and blood supply in endometrial cancer. Meanwhile, Radiomics has synergistic prognostic prediction potential with clinical markers, Pathomics, Transcriptomics, and Proteomics. The machine learning-based radiomics model showed outstanding performance in the prediction of postoperative overall survival, which can provide a reliable basis for prognostic prediction and clinical decision-making in EC patients.

## Supplementary Information

Below is the link to the electronic supplementary material.

Supplementary Material 1

## Data Availability

No datasets were generated or analysed during the current study.

## Abbreviations

ADC: Apparent diffusion coefficient
D: Diffusion coefficient
D^*^: Pseudodiffusion coefficient
DCE-MRI: Dynamic contrast-enhanced magnetic resonance imaging
DGE: Differential gene expression
DT: Decision Tree
EC: Endometrial cancer
f: Perfusion fraction
FIGO: International federation of gynecology and obstetrics
GBM: Gradient boosting machine
GEO: Gene expression omnibus
ICC: Intraclass correlation coefficient
IDI: Integrated discrimination improvement
IVIM-DWI: Intravoxel incoherent motion diffusion-weighted imaging
Kep: Rate constant
Ktrans: Transfer constant
LASSO: Least absolute shrinkage and selection operator
NB: NaiveBayes
NCCN: National comprehensive cancer network
NRI: Net reclassification improvement
Radscore: Radiomics scores
RF: Random Forest
ROI: Region of interest
RSF: Random survival forest
SVM: Support vector machine
TCGA: The cancer genome atlas
Ve: Extravascular Extracellular Volume
VOI: Volume of interest
Vp: Capillary plasma volume

## Declarations

### Guarantor

The scientific guarantor of this publication is Long Liu.

### Statistics and biometry

Yaqiong Ge kindly provided statistical advice for this manuscript.

### Research registration

This study is registered with the Chinese Clinical Trial Registry. (registration number ChiCTR2100043892). Website: https://www.chictr.org.cn/showproj.html?proj=122517.

### Study subjects or cohorts overlap

There are no studies with subjects or cohorts that overlapped with this study.

### Provenance and peer review

Not commissioned, externally peer-reviewed.
