# Supplementary material for: Machine learning-based radiomics model: prognostic prediction and mechanism exploration in patients with endometrial cancer
Source: Biomark Res. 2025 Sep 29;13:119. doi: 10.1186/s40364-025-00836-5 (PMC12481825; doi:10.1186/s40364-025-00836-5)
Supplement: Supplementary file 1 — Supplementary Material 1 [file 40364_2025_836_MOESM1_ESM.docx]

# Supplementary Material

## 1.Patients:

Data on endometrial cancer patients attending Centre 1 Anhui Provincial Hospital (training set, total 357 excluded 86), Centre 2 The First Affiliated Hospital of Anhui Medical University (validation set, total 212 excluded 58) and Centre 3 Funan Hospital (validation set, total 66 excluded 22) from 1 March 2015 to 1 March 2022 were collected retrospectively. Data were prospectively collected from endometrial cancer patients attending Centre 1 from 1 May 2020 to 1 May 2024 (test set 1, total 100) (registration number ChiCTR2100043892). The TCGA-UCEC cohort (8 cases) and CPTAC-UCEC (35 cases) cohorts were collected from the GDC (test set 2, due to some missing clinical data were not included in the clinical model validation). According to the inclusion and exclusion criteria finally 602 patients out of 778 were included in the study (Figure 1). All enrolled patients underwent total hysterectomy and adnexal resection and pelvic lymph node dissection. General clinical data including age, maximum tumour diameter, lymph node metastasis (LNM), pathological staging were collected from the hospital medical record system. FIGO staging was reclassified according to the latest guidelines. In this study, a combined approach of stepwise exclusion and mean imputation was adopted to handle missing data. First, patients with severely missing clinical data (with ≥ 2 items missing) or missing endpoint indicators (clinical outcomes) were excluded. Subsequently, for the remaining samples with mildly missing clinical variables, the mean imputation method was used for supplementation. There were no statistically significant differences in clinical data between the training and validation sets (Table S2). The study was approved by the ethics committees of the three centres and informed consent was waived by the ethics committee (Ethics No: 2024-YXK-08 (Centre 1), PJ2024-03-48 (Centre 2), FNLL202407023 (Centre 3)).

Inclusion Criteria: (a) Endometrial cancer confirmed by pathology; (b) Underwent MRI two weeks before therapy; (c) Without history of tumor therapy. (N1=357, N2=100, N3=212, N4=66, N5=43)

Exclusion Criteria: (a) Interrupted follow-up (N1=37, N2=5, N3=21, N4=6, N5=0); (b) Incomplete clinical data (Severe missing clinical data (≥2 items missing) or missing endpoint indicators (clinical outcomes)) (N1=42, N2=3, N3=33, N4=15, N5=0); (c) With other malignancies (N1=7, N2=2, N3=4, N4=1, N5=0).

N1 represents the Center 1 (training set), N2 represents the Center 1 (test set 1), N3 represents the Center 2 (validation set), N4 represents the Center 3 (validation set), and N5 represents the TCGA-UCEC&CPTAC-UCEC (test set 2).

## 2.MR scanning protocols:

All the MR images in Center 1 were obtained on a clinical whole-body 3.0T MRI scanner (GE Signa HDXT 3.0T MRI scanner, GE Healthcare, USA) with a phased-array 8-channel sensitivity-encoding abdominal coil. The 3.0T MRI scanner (Discovery MR750w, General Electric, USA) with an accompanying 8-channel phased-array body coil was uesed in Center 2. The 1.5T MRI scanner (Avanto, Siemens Healthcare, Erlangen, Germany) with a 16-channel phased array body coil was used in Centre 3. All patients had no contraindications to MR examination. Bowel preparation was performed by fasting 8 hours before the examination to reduce the influence of bowel contents on the scanning image. Appropriate water was consumed 1 hour before the examination to make the bladder full. The scanning range was from the upper edge of the iliac wing to the lower edge of the pubic symphysis. In patients with suspected retroperitoneal lymph node metastases the scan was performed from the top of the diaphragm to the lower edge of the pubic symphysis.

### 2.3 Conventional MR at Centre 3

Scanning sequences and parameters in Center 3: (1) Axial T1WI: TR 500 ms, TE 8 ms, slice thickness 5 mm, slice gap 1 mm, acquisition matrix 350 × 200, FOV 240 mm × 240 mm; (2) Axial and sagittal T2WI: TR 4600 ms, TE 68 ms, slice thickness 3 mm, slice gap 1 mm, acquisition matrix 320 × 256, FOV 240 mm × 240 mm; (3) Axial T2WI-FS: TR 5000 ms, TE 75 ms, slice thickness 6 mm, slice gap 2 mm, acquisition matrix 320 × 256, FOV 240 mm × 240 mm; (4) Sagittal T2WI: TR 4000 ms, TE 120 ms, slice thickness 5 mm, slice gap 1 mm, acquisition matrix 320 × 250, FOV 260 mm × 260 mm; (5) Axial DWI: TR 3900 ms, TE 78 ms, slice thickness 5 mm, slice gap 1 mm, acquisition matrix 96 × 130, FOV 240 mm × 240 mm, b-values taken as 0, 1 000 and 1500 s/mm^2^. A comparison of the three Centers scanning parameters is shown in Table S1.

### 2.1 Conventional MR at Centre 1

Scanning sequence and parameters in Center 1: (1) Axial T1WI: repetition time (TR) 500 ms, echo time (TE) 7 ms, slice thickness 6 mm, slice gap 2 mm, acquisition matrix 352×192, field of view (FOV) 320 mm×320 mm; (2) Axial T2WI: TR 4600 ms, TE 72.5 ms, slice thickness 6 mm, slice gap 2 mm, acquisition matrix 320 × 256, FOV 240 mm × 240 mm; (3) Axial T2WI with fat suppression (FS): TR 5000 ms, TE 72.5 ms, slice thickness 6 mm, slice gap 2 mm, acquisition matrix 320 × 256, FOV 240 mm × 240 mm; (4) Sagittal T2WI: TR 4500 ms, TE 72 ms, slice thickness 4 mm, slice gap 1 mm, acquisition matrix 320 × 320, FOV 280 mm × 280 mm; (5) Axial DWI: TR 4600 ms, TE 65 ms, slice thickness 4 mm, slice gap 1 mm, acquisition matrix 420 × 420, FOV 96 mm × 130 mm, b-values were used at 0 and 1000 s/mm^2^. The contrast agent was gadolinium dimeglumine (GE Pharmaceuticals) at a dose of 0.1 mmol/kg with a flow rate of 2 ml/s.

### 2.2 Conventional MR at Centre 2

Scanning sequences and parameters in Center 2: (1) Axial T1WI: TR 491 ms, TE 8.8 ms, slice thickness 5.0 mm, slice gap 0.5 mm, acquisition matrix 350 × 200, FOV 240 mm × 240 mm; (2) Axial and sagittal T2WI: TR 3773 ms, TE 128.7 ms, slice thickness 5 mm, slice gap 0.5 mm, acquisition matrix 320 × 250, FOV 240 mm × 240 mm; (3) Axial T2WI-FS: TR 5000 ms, TE 75 ms, slice thickness 6 mm, slice gap 2 mm, acquisition matrix 320 × 256, FOV 240 mm × 240 mm; (4) Sagittal T2WI: TR 3773 ms, TE 128.7 ms, slice thickness 5 mm, slice gap 0.5 mm, acquisition matrix 320 × 250, FOV 240 mm × 240 mm; (5) Axial DWI: TR 3950 ms, TE 78.8 ms, slice thickness 5 mm, slice gap 0.5 mm, acquisition matrix 100 × 130, FOV 240 mm × 240 mm, b-values taken as 0, 400, 1 000 and 1500 s/mm^2^.

### 2.4 IVIM-DWI at Centre 1

IVIM-DWI: IVIM-DWI was axial DWI with 10 b-values (0, 10, 20, 50, 100, 200, 400, 800, 1200, 2000 s/mm2). TR 4600 ms, TE 65 ms, slice thickness 4 mm, slice gap 1 mm, acquisition matrix 420 × 420, FOV 96 mm × 130 mm. The contrast agent was gadolinium diamine (GE Pharmaceuticals) at a dose of 0.1 mmol/kg at a flow rate of 2 ml/s, and the tube was flushed by injecting 20 ml of saline at the same flow rate.

### 2.5 DCE-MRI at Centre 1

DCE-MRI: (1) Transverse T1WI:TR of 540 ms, TE of 4 ms, layer thickness of 3 mm, layer spacing of 0.3 mm, FOV 300 mm × 250 mm, matrix 256 × 256; Transverse T2WI:TR of 4000 ms, TE of 100 ms, layer thickness of 3 mm, layer spacing of 0.3 mm, FOV 200 mm × 180 mm, matrix 320 × 320; (2) Sagittal T2WI: TR of 5500 ms, TE of 90 ms, layer thickness of 3 mm, layer spacing of 0.3 mm, FOV 240 mm × 200 mm, matrix of 256 × 256; (3) Transverse DWI: TR of 3 800 ms, TE of 95 ms, layer thickness of 3 mm, layer spacing of 1 mm, FOV 250 mm × 200 mm, b-value of 8 000 s/mm. (4) DCE-MRI scanning was performed with 3D volumetric fast acquisition, with the parameters of TR of 4.0 ms, TE of 1.5 ms, layer thickness of 3 mm, layer spacing of 1 mm, FOV 260 mm × 260 mm, matrix 256 × 256. (5) The 3D volumetric fast acquisition was performed with the following parameters: TR of 4.0 ms, TE of 1.5 ms, layer thickness of 3 mm, layer spacing of 1 mm, FOV of 260 mm × 260 mm, and matrix of 256 × 256. 0.2 mL/kg of gadopentetate-glucosamine contrast agent was injected by a high-pressure syringe through an intravenous channel with an automatic trigger at an injection rate of 2.5 mL/s. Dynamic-enhanced temporal phases were acquired prior to the injection, and dynamically-enhanced images were selected for 30, 60, 90, 120, and 180 s after the injection at an acquisition time of 18 s and an interval of 12 s.

## 3.Treatment and follow-up protocols:

### 3.1 Surgery

All included patients in this study received total hysterectomy and adnexa removal and pelvic lymph node dissection. Surgical modalities included laparoscopic surgery and open surgery. Patients with different stages of endometrial cancer were treated slightly differently:

Stage IA: uterus, bilateral tubo-ovariectomy + lymph node dissection (elective)

Stage IB: uterus, bilateral tubo-ovariectomy + lymph node dissection

Stage II: uterus, bilateral salpingo-oophorectomy + lymph node dissection + parametrial resection (selective)

Stage III: hysterectomy, bilateral salpingo-oophorectomy + lymph node dissection (PLND) + parametrial resection

Stage IV: palliative surgery + chemotherapy or radiotherapy.

Only patients with endometrial cancer who underwent extrafascial total hysterectomy + bilateral adnexal resection + pelvic lymph node dissection + para-abdominal aortic lymph node examination were included in this study. The main purpose of surgery is to completely remove the primary tumour and potential metastatic lesions in order to achieve radical treatment.

### 3.2 Follow-up

Follow-up method: all patients were followed up for a long time through outpatient clinic, inpatient review or telephone contact. Frequency of follow-up: every 3-6 months for 2 years, every 6-12 months for the 3rd-5th years, and once a year thereafter. Follow-up content: history taking, gynaecological examination, urine and faeces routine, cytology test, tumour markers, chest CT, abdominopelvic ultrasound/MRI, and other necessary examinations. All patients were followed up until death or January 2024. The endpoint indicators in this article include OS: time from the start of surgery to the patient's death or last follow-up.

## 4.Image analysis:

### 4.1 Radiomics

**4.1.1 Introduction:** Radiomics is a technique for high-throughput mining of quantitative image features from standard medical images. It enables image data to be extracted and applied in clinical decision support systems to improve diagnostic, prognostic and predictive accuracy in oncology research. This technique also occupies an increasingly important place in oncology research. Radiomics is dedicated to the development of sophisticated image analysis tools to achieve precision diagnosis and treatment using validated image features, providing a powerful tool for modern medicine. In recent years, medical imaging not only has an important role in clinical diagnosis, but also occupies a central position in precision medicine. In Radiomics, digitised medical images contain information related to tumor pathophysiology, which can be transformed into mineable high-dimensional data. Through quantitative image analysis, this information can be utilised and used to improve medical decision-making through clinical decision support systems (CDSS). The download link for all imaging data in Test Set 2 is: https://www.cancerimagingarchive.net/.

**4.1.2 Analysis:** The pre-treatment T2WI images were first imported into 3D slicer software in DICOM format. The N4ITK bias field correction module (https://www.slicer.org/wiki/) in 3D Slicer software (https://www.slicer.org/, version 4.10.2) was used for the analysis. Documentation/Nightly/Modules/N4ITKBiasFieldCorrection) was used to obtain a standard normal distribution of image intensities and to reduce possible heterogeneity prior to feature extraction. The tumour region of interest (ROI) was then outlined layer by layer by 2 senior imaging physicians (9 and 11 years of imaging experience, respectively). A 5 mm region outside the tumor ROI was used as the peritumor ROI to extract peri-tumour radiomics parameters. Each level of ROI was fused into a volume of interest (VOI) to extract 3D texture features. Finally, 1409 texture parameters were extracted from each image using PyRadiomics software. Z-score was used to standardise and normalise the texture parameters; ComBat was used to reduce the batch effect of the parameters. An overview of the image sketching and model construction is shown in Figure 2.

**4.1.3 Content:** The 1409 Radiomics features extracted by PyRadiomics can be categorized into the following eight classes: (1) Original (107): Original image features, which are directly extracted from the image without any transformation or processing, such as gray-level histogram and morphological features. (2) Wavelet (744): Wavelet transform features, which are extracted after applying wavelet transform to the image, capturing frequency and spatial information. (3) Square (93), (4) SquareRoot (93), (5) Logarithm (93), and (6) Exponential (93): These features are extracted after applying mathematical transformations to the image, such as squaring, square root, logarithm, and exponential transformations, which can alter the gray-level distribution and highlight different gray-level ranges. (7) Gradient (93): Gradient features, which calculate the gradient information of the image, reflecting edge and texture information. (8) Lbp (93): Local Binary Pattern (LBP) features, which describe local texture information of the image. These features can reflect different aspects of the image, such as gray-level distribution, spatial information, frequency information, and texture information, providing rich information for subsequent machine learning and data analysis.

**4.1.4 Reproducibility:** To evaluate the inter-observer reproducibility of ROIs in radiomics analysis, two radiologists (Dr. Fei Gao with 10 years of experience and Dr. Chao Wei with 8 years of experience) performed independent segmentations across the entire dataset. Inter-group consistency analysis was then conducted based on the initial ROIs delineated by both radiologists.

For assessing intra-observer reproducibility, 100 patients were randomly selected from Center 1. Both radiologists performed tumor segmentation twice for these patients (initial segmentation and repeated segmentation four weeks later). Subsequently, intra-group consistency analysis was performed based on the two sets of segmentations of the 100 images by the same radiologist.

The evaluation included one inter-group consistency analysis and two intra-group consistency analyses. An intraclass correlation coefficient (ICC) > 0.75 indicated good consistency. Results showed that 1409 features were extracted from each tumoral and peritumoral region. Among them, 997 parameters in tumoral regions and 890 parameters in peritumoral regions were highly robust, with ICC (assessed via one inter-group and two intra-group analyses) greater than 0.75.

The CLEAR was used for the evaluation of radiomics studies (Table S1). The RQS and METRICS were used to assess the research methodology, where the RQS score was 31 out of 36 (Table S2) and the METRICS score was 93.1% (Table S3).

**4.1.4 Results and Significance:**

In this study, a total of 16 intratumoral and 12 peritumoral radiomics features were incorporated into model construction (Tables S3 and S4).

Wavelet-Based Features: wavelet_LHH_firstorder_Skewness (skewness) exhibited a negative correlation with the Radscore but a positive association with endometrial carcinoma or favorable prognosis. This feature reflects the asymmetry of gray-level distribution, where negative values indicate a left-skewed histogram (e.g., necrotic regions). MRI manifestations include low cellular density and necrotic areas. wavelet_HHH_glszm_GrayLevelVariance (gray-level variance) showed a positive correlation with the Radscore but a negative association with endometrial carcinoma or poor prognosis. This metric quantifies the dispersion of gray-level intensities within a region, with higher variance indicating greater textural heterogeneity. MRI correlates include intratumoral cystic changes, hemorrhage, or calcification, where elevated variance often predicts higher malignancy. wavelet_HHL_glszm_SizeZoneNonUniformityNormalized (normalized size-zone non-uniformity) evaluates the spatial distribution heterogeneity of region sizes. A negative coefficient suggests higher uniformity, which was inversely correlated with the Radscore but positively associated with endometrial carcinoma or improved prognosis. MRI findings may correspond to homogeneous tumor architecture, indicating lower intratumoral heterogeneity and better clinical outcomes. wavelet_HHH_glrlm_HighGrayLevelRunEmphasis (high gray-level run emphasis) highlights contiguous high-intensity regions. A negative coefficient implies reduced high-intensity areas, correlating negatively with the Radscore but positively with endometrial carcinoma or favorable prognosis. MRI characteristics may reflect diminished contrast enhancement, suggesting lower vascularity, reduced proliferative activity, and improved survival.

Local Binary Pattern (LBP) Features: lbp_2D_glszm_GrayLevelNonUniformity (gray-level non-uniformity) measures the dispersion of gray-level intensities, with higher values indicating greater heterogeneity. It demonstrated a positive correlation with the Radscore but a negative association with endometrial carcinoma or poor prognosis. MRI patterns may reflect angiogenic activity or heterogeneous necrosis, suggesting adverse outcomes. lbp_2D_glszm_ZoneEntropy (zone entropy) reflects image complexity, where elevated entropy corresponds to structural disorganization. This feature was positively correlated with the Radscore but negatively associated with endometrial carcinoma or prognosis. MRI manifestations may indicate infiltrative tumor growth, portending worse survival.

Gradient-Based Features: gradient_gldm_SmallDependenceLowGrayLevelEmphasis emphasizes small, low-intensity dependent regions, potentially corresponding to micro-necrotic foci. It showed a positive correlation with the Radscore but a negative association with endometrial carcinoma or prognosis. MRI findings may indicate micro-necrosis, predictive of poorer outcomes.

Morphological Features: squareroot_glszm_LargeAreaLowGrayLevelEmphasis characterizes large, low-intensity regions. A negative coefficient suggests a reduction in such areas, correlating inversely with the Radscore but positively with endometrial carcinoma or improved prognosis. MRI observations may reflect decreased edema or cystic components, implying better tumor biology.

Other Key Features: Features such as original_glcm_lmc1 (local mean correlation) also demonstrated biological relevance. Further investigations are warranted to elucidate their clinical and mechanistic significance.

### 4.2 IVIM-DWI

**4.2.1 Introduction:** IVIM is an imaging method for describing voxel micro-motion, and its technical premise is to assume that the micro-circulation and perfusion of blood is a non-coherent, unstructured and random motion, and the micro-motion in the organism includes two components, the true diffusion of water molecules and the pseudo-diffusion formed by micro-circulation and perfusion, and IVIM imaging evaluates the diffusion component and the perfusion component of the blood flow through quantitative parameters, respectively. The relationship between the signal change and all the b-values can be expressed by the following equation1: Sb/S0 = (1-f).exp(-bD)+f.exp[-b(D*+D)]

S0 and Sb represent the signal intensity when b = 0 and when b is other values (10, 20, 30, 50 ......), respectively. b value is the diffusion sensitivity factor dependent on the scanning sequence in s/mm2. f value is the perfusion fraction, which represents the volumetric ratio of the diffusion of microcirculatory perfusion effects to the overall diffusion effect within the voxel, with a magnitude ranging between 0 -1; D value is the pure diffusion coefficient, representing pure water molecule diffusion motion (slow diffusion motion component), also known as slow pool diffusion, in mm2/s; D* value is the pseudo-diffusion coefficient generated by the blood circulation, representing the incoherent motion of the microcirculation within the voxel, i.e., perfusion-related diffusion motion, or fast diffusion motion, in mm^2^/s. Considering that D^*^ is significantly larger than D, when b＞ 200 s/mm^2^, its effect on signal attenuation is negligible, and Equation 1 above can be simplified to Equation 2 as: SI/SI0 = e(-bD)

At this time, the D value can be obtained by Eq. 2, using multiple b-values for IVIM-DWI imaging, together with the nonlinear fitting algorithm based on Eq. 1, the f value and D* value can be obtained. Applying the IVIM model to the multiple b-value DWI, the perfusion-related parameters (ƒ, D*) and diffusion parameters (D) can be obtained simultaneously, which can be used to quantify the two components of motion in the DWI image.IVIM-DWI can provide perfusion information in addition to information on the pure diffusion motion of the water molecules, which provides more information about the characteristics of the tumour, and the response to tumour treatment.

**4.2.2 Analysis:** IVIM-DWI was analysed by two senior radiologists independently and manually sketching ROIs layer by layer on a GEAW 4.5 workstation. Images were selected from sequences with b=800 s/mm^2^. ROIs were avoided as much as possible by avoiding tumor metaplasia, haemorrhage, necrosis, etc. The software automatically generated the apparent diffusion coefficient (ADC), Diffusion Coefficient (ADC) and Perfusion Fraction (f). The software automatically generated each parameter of IVIM-DWI, Apparent Diffusion Coefficient (ADC), Diffusion Coefficient (D), Perfusion Fraction (f) and Pseudodiffusion Coefficient (D*). All data were measured three times for averaging. The inter-group ICC values of IVIM-DWI and DCE-MRI in Table S12.

### 4.3 DCE-MRI:

**4.3.1 Introduction:** DCE-MRI imaging examination mainly relies on the microcirculation changes of lesions and tissues, and obtains a series of consecutive dynamic enhancement process images that can reflect the strengthening of tissues in various periods before, during and after the injection of contrast agent through continuous and rapid imaging sequences, then through the processing and analysis of the image information obtained by the corresponding computer software, various parameters reflecting the function of the microcirculation of the tissues can be obtained. The image information obtained can be processed and analysed by the corresponding computer software to obtain various parameters that can reflect the function of tissue microcirculation. Through the analysis of these parameters, we can indirectly deduce the inflow, spreading and contouring of the contrast agent in the lesions or tissues, so as to more objectively analyse the enhancement differences between the lesions and normal tissues, and not only analyse the morphological changes of the lesions, but also explain the changes in the physiological functions of the lesions in a more quantitative point of view. Therefore, for this emerging technology, the function of tissue microcirculation and the alteration of microcirculation function of lesions are its physiological basis. In some lesions, such as tumours, inflammation, etc., the perfusion and/or vascular permeability in the lesion area may be altered, and can cause differences in the distribution and metabolism of substances between the lesion and the normal tissue, and it is by capturing such microscopic differences that DCE-MRI can achieve quantitative analysis of tissue examination. Commonly used parameters are volume transfer constant (Ktrans), rate constant (Kep), extravascular and extracellular interstitial volume fraction (Ve), etc. In the TKmodel model, the forward transfer constant, Ktrans (min-1), and the reverse constant, Kep (min-1), represent the process of contrast from the vasculature to the tissue interstitial space and the return of contrast to the intravascular contour, respectively. The volume of the extravascular-extracellular gap is expressed in terms of Ve, which can be calculated to obtain the equation: Kep = Ktrans/Ve. Ktrans is considered to be one of the most important parameters, and where tissue capillaries have a high permeability, Ktrans reflects the plasma flow per unit volume of tissue, whereas where tissue capillaries have a low permeability, Ktrans reflects the permeable surface area of tissue per unit volume. In most cases, since the distribution of contrast agent in a unit of tissue is affected by the permeability of tissue capillaries and also depends on the plasma flow in the region, Ktrans reflects the joint effect of capillary permeability and plasma flow in the tissue, and the higher the value of Ktrans, the higher the plasma flow and vascular permeability of the tissue, and therefore the Ktrans value for tumours is the same as that for tumours. Therefore, for a tumour, the higher the Ktrans value, the faster its metabolism and the higher its malignancy may be.

**4.3.2 Analysis:** Data from DCE-MRI were processed using GE Omni Kinetic software. Manual outlining of the images was performed independently by two senior radiologists. The outlining level was selected as the level with the largest tumour and at the same time clear internal iliac artery imaging. Transfer Constant (Ktrans), Rate Constant (Kep), Plasma Volume Fraction (vp) and Extravascular Extracellular Space Volume Fraction (Ve) were calculated for the region of interest according to the TKmodel. All data were measured three times and averaged.

## 5.Construction of radiomics models:

1409 features were extracted from each of the tumor and peritumor. Among the tumour radiomics features, 997 high robustness parameters with intra- and inter-group ICC greater than 0.75 were identified. After removing redundant parameters with correlation greater than 0.8, the remaining 216 radiomics features entered LASSO feature selection. The final 16 radiomics features were used for machine learning modelling. Among the peri-tumour radiomics features, there were 890 high robustness parameters, and 141 radiomics features remained after redundancy removal. The remaining 12 radiomics features after LASSO screening were used for machine learning modelling. The CLEAR was used for the evaluation of Radiomics studies (Table S5). The RQS and METRICS were used to assess the research methodology, where the RQS score was 31 out of 36 (Table S6) and the METRICS score was 93.1% (Table S7).

The optimal regularization parameter (λ) was determined through 10-fold cross-validation (with the minimization of cross-validation error as the criterion) and evaluated for feature selection stability using 1000 bootstrap samples. All features were standardized (Z-score), and a fixed random seed (seed=123) was used for the cross-validation process. In the radiomic feature selection for tumors, the LASSO model (λ=0.0125) achieved a C-index of 0.767 (95% CI: 0.712-0.822) on the test set, retaining 16 features with non-zero coefficients (all features had a bootstrap selection frequency >80%). For the peritumoral region model (λ=0.0445), the C-index was 0.733 (95% CI: 0.681-0.785), with 12 features retained. Cross-validation and bootstrap results indicated that the risk of model overfitting was well-controlled.

The remaining 16 tumoral and 12 preitumoral radiomics features after LASSO screening were used for machine learning modelling. Modelling methods included: aorsf, bart, dephit, Deepsurv, dnnsurv, GBM, glmnet, RSF, SVM, XGboost, where the model with the best predictive efficacy was named the best radiomics score Radscore. The DeLong test for XGBoost and other models is shown in Table S8.

Radiomics features in the tumor model include: wavelet_LLH_firstorder_Minimum, wavelet_LHH_glcm_Imc2, wavelet_LHH_firstorder_Skewness, wavelet_HHL_glszm_ SizeZoneNonUniformityNormalised, wavelet_HHH_glszm_GrayLevelvariance, wavelet_HHH_gIszm_GrayLevelNonUniformityNormalized, wavelet_HHH _gIrim_LowGrayLevelRunEmphasis, wavelet_HHH_glrIm_HighGrayLevelRunEmphasis, squareroot_glszm_LargeAreaLowGrayLevelEmphasis, square_ firstorder_Energy, Iogarithm_glszm_LargeAreaLowGrayLevelEmphasis, Ibp_2D_gIszm_ZoneEntropy, Ibp_2D_glszm_ SizeZoneNonUniformityNormalised, Ibp_2D_gIszm_GrayLeveINonUniformity, gradient_ngtdm_Strength and gradient_glcm_Mcc.

Radiomics features in the peritumor model include: wavelet_LLH_firstorder_Uniformity, wavelet_HHL_gIszm_SizeZoneNonUniformityNormalised, wavelet_HHH_glszm_ GrayLevelvariance, wavelet_HHH_glszm_GrayLevelNonUniformityNormalised, squareroot_glcm_Difrerencevariance, square_firstorder_Range, origina_glcm_Imc1, Ibp_2D_glszm_ZoneEntropy, Ibp_2D_gIszm_SizeZoneNonUniformityNormalised, Ibp_2D_gIszm_GrayLevelNonUniformity, gradient_gldm_SmallDependenceLowGrayLevelEmphasis and gradient_glcm_Mcc.

## 6.Combination of radiomics and clinical parameters:

All clinical information including age, gender, BMI, FIGO Stage, LNM, CA125, Differentiation, Clinical types, Histological type were included in the statistical analysis. Continuous variables were transformed into categorical variables by the survminer function to find the best truncated values.Spearman's correlation analysis showed that Clinical types were excluded because they had a correlation of more than 0.75 with the differentiation level and had a lower predictive efficacy. One-way analyses were performed using the LogRank test of Kaplan-Meier analysis and one-way cox regression analysis. FIGO stage, LNM and differentiation level were significantly associated with postoperative survival in endometrial cancer in LogRank analysis (P<0.05).FIGO stage, LNM and differentiation level also showed statistical differences in one-way cox regression analysis (P<0.05). The above parameters with prognostic value were included in the multifactorial COX regression analysis together with Radscore. The results showed that FIGO stage, differentiation level and Radscore were independent prognostic factors for endometrial cancer patients (P<0.05). A joint model was constructed based on the above independent prognostic factors with AUCs of 0.940, 0.992, and 0.927 (training set) and 0.884, 0.915, and 0.909 (validation set) and 0.872, 0.881, and 0.794 (test set 1), respectively. The validation set (AUC of 0.885-0.943) and the test set (AUC of 0.805-0.963) suggest stable model efficacy. the NRI (0.071-0.249) and the IDI (0.137-0.657) suggest that the addition of Radscore significantly improves the overall predictive efficacy of the model (Figure S3, Table S9).

## 7.Radiomics in conjunction with Pathomics, Transcriptomics and Proteomics

Radiomics, pathomics, transcriptomics and proteomics parameters were collected from 35 patients of CPTAC-UCEC. LASSO model was used to construct pathomics, transcriptomics model and proteomics model. Radiomics models were combined with pathomics, transcriptomics and proteomics models to analyse their complementary value in the prognostic prediction of endometrial cancer. The results showed that radiomics, pathomics, transcriptomics and proteomics have high complementary values in the prediction of overall survival after endometrial cancer surgery. The combined model based on the multi-omics model demonstrated strong joint value and predictive efficacy in the prediction of OS in endometrial cancer, with 1-, 3-, and 5-year AUCs of 0.989, 0.996, and 1.000 (Figure 5&Table S10). The model can accurately stratify endometrial cancer patients according to prognostic risk. The download link for CPTAC-UCEC pathology, transcriptomics, and proteomics data is: https://dctd.cancer.gov/programs/occpr.

### 7.1 Pathomics:

Pathomics extracts histopathological features from H&E-stained images using computational tools. Features include texture, intensity, and cellular granularity, standardized for prognostic modeling.

H&E stained images were used for extraction of pathohistological parameters. Qupath was used to segment the pathology images into several tiles of size 1000×1000 pixels and the tiles with poorly filled images were excluded.Macenko's method was used to normalise the colour of the tiles.CellProfiler software was used for the extraction of pathology histology features. Application modules include "UnmixColors", "ColourToGray", "MeasureImageQuality", "MeasureImageIntensity", "MeasureColocalisation" and "MeasureGranularity ". For each tile, 1297 pathohistological parameters were extracted and the parameters were standardised and normalised using the Z-score method (the mean of the Tile was used for statistical analysis). Spearman was used to remove redundant pathohistological parameters (correlation > 0.9). The least absolute shrinkage and selection operator (LASSO) was used for feature selection and model construction.

The parameters in the pathomics model include: Texture_SumAverage_MaskBIue, Texture_Differencevariance_Rescalelntensity, Texture_DifferenceEntropy_RescaleIntensity, Texture_AngularsecondMoment_EnhanceOrsuppressFeatures, Granularity_12_GaussianFilter, Correlation_K_MaskBIue_GrayHE, Correlation_K_ Eosin_RescaleIntensity, Correlation_Costes_Rescalelntensity_Morphologicalskeleton and Correlation_Correlation_EnhanceorSuppressFeatures _Eosin.

### 7.2 Transcriptomics

Transcriptomics analyzes expression levels of RNA molecules (e.g., mRNA) to identify tumor-associated gene signatures. Redundant features are filtered, and key biomarkers are selected for prognostic evaluation.

Transcriptomic information was extracted for 35 patients in CPTAC-UCEC. Spearman was used to eliminate redundant transcriptomic parameters (correlation >0.9). The least absolute shrinkage and selection operator (LASSO) was used for feature selection and model construction.

The transcriptomics parameters in the model included LIM2, LHX8, HYALP1, FLJ40288, FGF16, ECPAS, DPRX, C15orf40, BCAR4, B3GAT2 and ALKBH6

### 7.3 Proteomics

Proteomics quantifies tumor-related protein expression profiles. Selected proteins serve as biomarkers for risk stratification and outcome prediction. Proteomic information was extracted from 35 patients in CPTAC-UCEC. Spearman was used to remove redundant proteomic parameters (correlation >0.9). The least absolute shrinkage and selection operator (LASSO) was used for feature selection and model construction. Proteomic parameters in the model included C9orf16, C9, C4BPB, C21orf91, BLOC1S1, BEND5, AP3M1, AOC1, ANKRD22, ALDH1L2, AKAP11, AKAP1, AGTPBP1, AGPAT4, ACTL8 and ABLIM3.

The multi-omics model proposed in this study can achieve clinical translation through the following pathways: Decision Support Stage: Radscore will be integrated as an automatically calculated parameter in the electronic medical record system. After patients complete preoperative MRI examinations, the system will automatically generate risk prompts (such as red/yellow/green light grading) to assist in formulating surgical plans.

Treatment Intervention Stage: For high-risk patients: Comprehensive staging surgery combined with sentinel lymph node biopsy is recommended, and extended-field radiotherapy plus paclitaxel/carboplatin chemotherapy should be considered postoperatively.

For intermediate-risk patients: After standard staging surgery, the intensity of adjuvant therapy will be adjusted based on pathological omics findings. For low-risk patients: Fertility-preserving surgery or reduced adjuvant therapy may be considered.

Dynamic Monitoring Stage: New radiomics features will be obtained through regular imaging follow-ups, and deep learning models will be used to dynamically update risk predictions, enabling timely adjustments to treatment strategies. In particular, early intervention will be initiated for patients who were initially low-risk but show feature drift during follow-up.

Biological Mechanism-Guided Treatment: For patients with radiomics-indicated FLT1-related high vascular features, FLT1 inhibitors (such as sorafenib) or VEGF-targeted therapy can be prioritized during the recurrence and metastasis stage to achieve cross-omics precision treatment.

### 7.4 Multi-omics models

The multi-omics model in this study integrates multi-dimensional features from Radiomics, Pathomics, Transcriptomics, and Proteomics. For 35 endometrial cancer patients, the AUC values for predicting 1-year, 3-year, and 5-year OS reached 0.989, 0.996, and 1.000, respectively. Due to the current limitation of the dataset sample size (n=35), external validation cannot be performed temporarily; however, we conducted systematic internal validation through 5-fold cross-validation. During the cross-validation process, the dataset was randomly divided into 5 subsets. In each iteration, 1 subset was selected as the validation set, and the remaining 4 subsets served as the training set, with the modeling and validation process repeated 5 times. The results showed that the mean cross-validation AUC for 1-year OS prediction was 0.972±0.015, for 3-year OS prediction it was 0.981±0.011, and for 5-year OS prediction it was 0.989±0.008. The fluctuation of validation results across different folds was small, indicating a low risk of overfitting for the current model and good stability and generalization ability within the internal dataset.

In addition, multi-omics models may capture key biological drivers of OS, providing a multi-faceted interpretation of prognostic information for endometrial cancer patients. Thus, the AUC values for predicting 1-year, 3-year, and 5-year OS all reached high levels. Meanwhile, we speculate that the result of the 5-year OS prediction AUC being close to 1.000 may be related to the relatively clear distribution of clinical outcome events at this time point (with a high incidence of endpoint events and complete follow-up data).

The AUC value reaching 1.000 may be related to the small sample size and excessive model complexity. In the future, we will further validate the predictive performance and clinical application value of this model by expanding the sample size and conducting multi-center external validation.

## 8.Biological mechanisms of the radiomics model:

Forty-three (8 TCIA-UCEC) and (35 CPTAC-UCEC) patients were used for the analysis of the biological mechanisms of the radiomics model. CPTAC-UCEC patients were stratified according to the radiomics score (cut-off value was derived from the surv_cutpoint function).Radscore cut-off value was 1.690. cut-off was defined by P<0.05, LogFC absolute value >1. The transcriptome had 1865 differential genes (1693 up-regulated and 172 down-regulated), functionally enriched for epithelial cell proliferation and vasculature system regulation (Figure S6). The proteome had 332 differential genes (up-regulated 244, down-regulated 88) with functional enrichment in endothelial cell migration and hypoxia-responsive pathways. The transcriptome and proteome intersected with a total of 307 genes, functionally enriched in angiogenesis, hypoxia response, etc. (Figure 6).Top10 hub genes were identified by Cytoscape: HIF1A, AKT1, FGF2, FLT1, KDR, MMP9, THBS1, TGFB1, ANGPT1, and MMP2. Among them, FLT1 was associated with Radscore most correlated (r=0.85, P=0.001).

In this study, Spearman's correlation analysis was performed to calculate the correlation coefficients and raw P-values for the associations between 10 hub genes and Radscore individually. Among them, gene FLT1 exhibited the highest correlation with Radscore, with a correlation coefficient of 0.85, showing a significant strong positive correlation trend. The correlation coefficients of the remaining 9 genes ranged from -0.54 to 0.61, among which 2 genes showed negative correlations and 7 genes showed positive correlations of varying degrees, with overall correlation strengths all lower than that of FLT1.

Since this step involved multiple hypothesis tests for 10 independent genes, to strictly control the risk of false positive results, we applied the Benjamini-Hochberg (BH) method for false discovery rate (FDR) correction and calculated the corrected q-values. Using a corrected q-value < 0.05 as the significance criterion, only 1 core gene (FLT1) with a significant correlation with Radscore was finally identified. Its raw P-value was 0.001, and the q-value corrected by the BH method was 0.008, which was far below the preset threshold, indicating that the false positive risk of this association result was controlled within 0.8%. Among the remaining 9 genes, the gene with a correlation coefficient of 0.61 had a raw P-value of 0.018 and a corrected q-value of 0.065; the gene with a correlation coefficient of -0.54 had a raw P-value of 0.025 and a corrected q-value of 0.083. The corrected q-values of the other genes ranged from 0.112 to 0.368, all higher than 0.05, failing to meet the significance criterion. This correction process effectively eliminated false positive associations caused by random errors, ensuring the reliability of the conclusion regarding the significant correlation between FLT1 and Radscore.

TCGA-UCEC (545 cases) and GSE205209 (54 cases) were used for functional analysis of Radscore and validation of survival analysis of hub genes.GSE173682 (5 single-cell samples) was used for expression validation of FLT1.WGCNA clustering analysis was used for clustering of transcriptome genes in endometrial cancer (in TCGA-UCEC cohort of 545 patients). Of these, the MElightcyan cluster genes (274 genes) had the strongest Radscore correlation, with functional enrichment in angiogenic and cell cycle pathways.Analysis of 5 single-cell samples from GSE173682 showed that FLT1 was expressed predominantly in the endothelial cells of endometrial cancer tissues, and to a lesser extent in tumour cells and mesenchymal cells.

## 9.Validation of biological function:

Retrospective analysis (1 March 2015 to 1 March 2022) suggested that radiomics modelling may be associated with the level of angiogenesis and blood supply in endometrial cancer. We therefore prospectively collected (1 May 2020 to 1 May 2024) IVIM-DWI and DCE-MRI parameters from 90 patients to reveal the association of radiomics models with angiogenesis (registry number ChiCTR2100043892). The results showed that the D* and f values in IVIM-DWI and the Ktrans and Kep values in DCE-MRI parameters (indirectly reflecting the angiogenesis and blood supply of the tumour tissues) were significantly correlated with Radscore (Spearman's correlation greater than 0.6, P less than 0.05), which suggests that patients with a high Radscore tend to have a more vigorous blood supply and angiogenesis.The prognostic value of IVIM-DWI and DCE-MRI parameters for endometrial cancer was also revealed. f (mainly reflecting the overall level of blood supply) and D* (mainly reflecting the blood flow velocity) in IVIM-DWI, and Ktrans (mainly reflecting vascular permeability and plasma flow), Kep (mainly reflecting vascular permeability), Vp ( mainly reflecting vascular density) and Ve (mainly reflecting the volume of extravascular-extracellular space) demonstrated a predictive value for postoperative survival in endometrial cancer. This not only reaffirms the prognostic value of blood supply levels in endometrial cancer patients, whose prognostic value has been demonstrated in a variety of tumours, but also provides new ideas and tools for prognostic prediction in endometrial cancer patients. We also analysed the FLT1 expression level in 30 patients (due to the limited collection of pathological data, only the FLT1 expression in 30 patients in test set 1 was analysed), which further proved the correlation between FLT1 and Radscore.The expression level of FLT1 was also significantly correlated with the f value, Ktrans and Kep values, which once again proved the correlation between radiomics modelling and correlation of endometrial cancer angiogenesis and blood supply levels.

## 10.Validation of molecular mechanisms:

Endometrial cancer cell lines AN3CA and Ishikawa as well as human umbilical vein endothelial cells HUVEC were used for cellular experiments (purchased from Shanghai Academy of Sciences, China). The endometrial cancer cell lines Ishikawa and AN3CA were used to knock down FLT1 expression by siRNA (WB and PCR proved that the knockdown was successful). Scratch experiments demonstrated that endometrial cancer cells (AN3CA and Ishikawa) in the FLT1 knockdown group had weaker migration ability. Angiogenesis assay was performed after co-culturing HUVEC with FLT1 knockdown AN3CA/Ishikawa, and the angiogenic ability (number, length, and ring-forming ability) was weakened compared to normal HUVEC cells. Ghost pen cyclin staining of actin suggested that knockdown of FLT1 reduced the invasion and migration of endometrial cancer cells, but had little effect on actin expression and function. cck8 cell proliferation assay suggested that the proliferation of AN3CA and Ishikawa was reduced after knockdown of FLT1. Transwell assay suggested that the migration of AN3CA/Ishikawa cells was reduced after knockdown of FLT1. migration ability was weakened. Plate cloning assay, suggested that knockdown of FLT1 attenuated the proliferation level of AN3CA cells. However, there was no statistically significant difference in plate cloning assay for Ishikawa.

### 10.1 Experimental reagents:

GAPDH antibody, Proteintech, China

FLT1 antibody, Proteintech, China

CD31 antibody, Proteintech, China

DAPI, Biyun Tian, China

siRNA, Ribobio, China

AN3CA, Ishikawa and HUVEC cell line was purchased from Shanghai Academy of Sciences, China

### 10.2 Experimental methods:

AN3CA and Ishikawa cells were cultured in DMEM medium containing 10% fetal bovine serum by volume [100 U-mL penicillin/streptomycin was added to the medium (the content of penicillin was 10 kU-mL, and the content of streptomycin was 10 g L, and the cells were placed in a humidified incubator at 37,5% CO by volume for 72 h)]. HUVEC were cultured in endothelial cell-specific complete medium. FLT1 Si-RNA was transfected for 48 h at a concentration of 100 nmol/L, and the transfection reagent was Lipofectamine 3000. The transfection time was 48 h. The total RNA was extracted using the TRIzo kit, and the real-time quantitative PCR substances were as follows: FLT1 upstream primer 5-AAGGTCTACAGCACC-AAG-3, downstream primer 5-CACATCATCAGAGCTTCC-3. GAPDH upstream primer 5-GAAGGTGAAGGTCGGAGTC-3, downstream primer 5-GAAGAGAGGTGATGGGGATTTC-3. Western blot primary antibody FLT1 incubation volume concentration of 1: 1000, GAPDH incubation volume concentration of 1: 1,0000. Cell proliferation assay was performed using CCK8 reagent, 1x10^4^ cells per well in 96-well plates cultured with melted down and unknocked down FLT1 of AN3CA/Ishikawa cells. 10 uL of CCK8 volume solution was added to each well, and the absorbance value was measured at a wavelength of 450 nm using an enzyme meter. Migration assays were performed in a 24-well Transwell cell chamber. 3-5 × 10^4^ indicated cell lines were seeded on the coated filters in 100 μl of serum-free medium, the bottom chamber was filled with 600 μl complete culture medium. After 24 h incubation at 37 °C in 5% CO_2_, the invasive ESCC cells were stained with crystal violet. Plate cloning experiments were performed by inoculating 1000 cells into six-well plates, which were continuously cultured for 14 days, stained with crystal violet, and photographed. Cells in the crawls were fixed using 4% formaldehyde (without methanol), permeabilized with 0.4% Triton X-100 and stained by adding ghost pen cyclic peptide. The nuclei of the cells were restained using DAPI solution and then photographed. Angiogenesis experiments were performed after co-culturing HUVEC with AN3CA/Ishikawa knocked down FLT1. 20 ul of matrix gel was evenly spread in a 24-well plate and 100 ul of cell suspension with a concentration of 1-5 × 10^5^ HUVEC cells/ml was dropped. Photographs were taken at 0h, 6h, 12h and 18h. The number and length of tube and the number of rings formation were counted.

## 11. Statistical analysis

Statistical analysis was performed by R4.3.2 and SPSS27.0 software. Radiomics parameters were first standardised and normalised using the Z-score function of the scaling software package. The ComBat function was used to reduce the batch effect caused by different acquisition times. ICC was used to test the robustness of radiomics parameters, and only highly robust parameters with intra-group and inter-group ICC greater than 0.75 were included in the subsequent analyses. Spearman/Pearson correlation analysis (cor function) was used to remove redundant parameters with correlations greater than 0.80. The correlation function was also used to remove redundant parameters with correlations greater than 0.80. Pearson correlation analysis (cor function) was used to remove redundant parameters with correlations greater than 0.80. Minimum absolute shrinkage and selection operators (glmnet package) were used for radiomics feature selection. gbm package, randomForest package, survivalsvm package, xgboost package, and mlr3 package were used to construct the GBM, RSF, SVM, and XGboost models and Aorsf, Bart, Deephit, Deepsurv, Dnnsurv, and Glmnet models, respectively. rms package and forestmodel package were used to construct nomograms and forest maps. survminer package was used to derive truncated values, and the survfit function was used for LogRank tests. The survivalROC, rmda, ggDCA, and survIDINRI packages were used to plot time-dependent ROC curves, calibration curves, decision curves, clinical impact curves, net reclassification indices, and overall discrimination indices of the models. limma package was used for variance analyses. clusterProfiler package and enrichplot package were used for enrichment analyses. wgcna package was used for co-expression clustering analyses. The Seurat package was used for single-cell RNA sequence data analysis, including data preprocessing, dimensionality reduction, clustering, differentially expressed gene identification, cell type identification, etc. The stringr package, the ggcorrplot package, and ggplot2 were used for correlation analysis and plotting. In this study, systematic testing was performed for the candidate covariates in the univariate Cox regression and the final included covariates in the multivariate Cox regression. The statistical test of Schoenfeld residuals was calculated using the `cox.zph` function in R. The results showed that the P-values for all individual tests of covariates and the global test of the model were > 0.05, indicating no significant association between the residuals and time. All code has been shared to github: https://github.com/zhangyu199831/Endometrial-Cancer.

## 12. Training process of 10 machine learning methods

The detailed training process and finely-tuned parameters for each individual model are as follows:

**1. XGBoost**

Data Preparation

This study uses retrospective cohort data, importing the training set and external validation set from CSV-format files. The core objective is to construct a survival prediction model based on XGBoost. The data preprocessing process is as follows: For key variable identification, Z-score was used to standardize radiomics parameters; ComBat was used to reduce the batch effect of parameters. "time" (survival time) and "status" (event status) were identified as core variables for survival analysis. The response variable required by XGBoost was constructed using ifelse (status == 1, time, -time), with all other variables serving as predictive features. Covariates from column 3 to the last column in the data were used as predictive features and converted into matrix format (x_train and x_test) to adapt to the input requirements of the XGBoost model (xgb.DMatrix).

Model Selection and Parameter Optimization

The survival model of the XGBoost framework (objective = "survival:cox") was selected. It integrates multiple decision trees through gradient boosting, which can effectively capture nonlinear relationships and feature interaction effects in survival data, with a built-in regularization mechanism to reduce the risk of overfitting. Based on the characteristics of survival models and the advantages of XGBoost, the parameter grid (param_grid) is designed as follows:

- eta range: 0.01, 0.05, 0.1, 0.3. Description: Learning rate that controls the contribution weight of each tree; smaller values require more iterations and reduce the risk of overfitting.

- max_depth range: 3, 5, 7. Description: Maximum depth of the tree, which controls the complexity of a single tree; larger values enhance the model's fitting ability but increase the risk of overfitting.

- min_child_weight range: 1, 3, 5, 8. Description: Defines the minimum sum of Hessian values required in a leaf node. In survival analysis with Cox loss, it controls the minimum 'confidence' needed to split a node—smaller values (e.g., 1) allow more complex trees with smaller leaf nodes (risk of overfitting), while larger values (e.g., 8) enforce simpler trees with more conservative splitting (reducing overfitting but requiring careful tuning to avoid underfitting). This parameter was optimized in conjunction with max_depth to balance model complexity and generalization, as its regularization effect is often more nuanced than depth constraints alone.

- subsample range: 0.6, 0.8, 1. Description: Proportion of samples sampled during the training of each tree, reducing correlation between samples to mitigate overfitting.

- colsample_bytree range: 0.6, 0.8, 1. Description: Proportion of features sampled during the training of each tree, enhancing model randomness and improving generalization ability.

- alpha range: 0, 0.1, 0.5. Description: L1 regularization coefficient that penalizes high-weight features to reduce model complexity.

- lambda range: 0, 0.1, 1. Description: L2 regularization coefficient that further controls overfitting by penalizing large coefficients and improves model stability.

Fixed parameters:

- objective = "survival:cox": Uses the Cox proportional hazards model as the underlying function to generate risk scores, adapting to the risk prediction needs of survival data.

- eval_metric was adjusted to Harrell's C-index (calculated using the survConcordance function from the survival package), which quantifies the model's ability to correctly rank survival times across all time points; a higher value (closer to 1) indicates stronger discriminative power.

- Cross-validation folds: 5 stratified folds (nfold = 5), with stratification based on event status to maintain consistent event proportions across folds, balancing evaluation stability and computational efficiency.

Evaluation Metrics

Combining the time-dependent characteristics of survival data, a multi-level evaluation system was constructed:

- Primary metric: Time-dependent AUC. AUC values of the training set and validation set at specific time points (12, 36, 60 months, corresponding to 1 year, 3 years, and 5 years) were calculated using the timeROC package. It quantifies the model's ability to distinguish between high-risk and low-risk individuals at different time points; an AUC closer to 1 indicates stronger discriminative ability.

- Secondary metric: Harrell's C-index, which provides an overall measure of the model's ability to rank survival times across all time points, with higher values indicating better performance.

Parameter Optimization Process

An efficient optimization framework combining random search and stratified 5-fold cross-validation was adopted to explore the optimal parameter configuration while controlling computational costs. Stratification was performed based on event status (1 = event occurred, 0 = censored) to ensure consistent event proportion across folds, which is critical for survival data with potential imbalance in event distribution.

- Parameter search strategy: Since the parameter grid contains 4×3×4×3×3×3 = 1296 combinations, direct grid search is computationally expensive. Therefore, 50 parameter sets were randomly selected for evaluation to balance search efficiency and parameter space coverage.

- Cross-validation mechanism: Stratified 5-fold cross-validation was implemented by integrating xgb.cv with custom C-index calculation. For each fold, after generating risk scores from the validation subset, Harrell's C-index was computed using survConcordance(Surv(time, status) ~ risk_score). The mean C-index across all 5 folds was used as the performance metric for each parameter set.

- Early stopping strategy: An early stopping mechanism (early_stopping_rounds = 10) was adopted — iteration stops when the validation set's C-index does not improve for 10 consecutive rounds to avoid overfitting and determine the optimal number of iterations (best_iter).

- Optimal parameter selection: The maximum mean cross-validation C-index was used as the objective function to select the parameter combination (best_params) with the highest corresponding score and the optimal number of iterations, ensuring a balance between model performance and generalization ability.

Model Validation and Result Output

The final model (xgb_model) was trained using the entire training set, optimal parameters (best_params), and optimal number of iterations (best_iter). Changes in C-index of the training set and validation set were monitored in real-time through the watchlist to ensure model convergence.

Final optimal parameters: max_depth=5; min_child_weight=3; subsample=0.8; colsample_bytree=0.8; alpha=1; lambda=10; eta=0.05; nrounds=300.

Model performance:

- C-index: 0.905 (training set); 0.858 (validation set); 0.821 (Test Set 1); 0.813 (Test Set 2)

- AUC (1-year, 3-year, 5-year): 0.916, 0.913, 0.899 (training set); 0.862, 0.885, 0.870 (validation set); 0.823, 0.869, 0.849 (Test Set 1); 0.850, 0.731, 0.800 (Test Set 2).

**2. Aorsf**

Data Preparation

This study adopts a retrospective cohort design, importing clinical datasets from CSV-format files. For constructing survival analysis tasks, the "time" variable was used as survival time, and the "status" variable as the event indicator, with an mlr3 survival analysis task object created. Z-score was used to standardize radiomics parameters; ComBat was used to reduce the batch effect of parameters.

Model Selection and Parameter Optimization

The accelerated orthogonal random survival forest (aorsf) algorithm was selected as the base model, which combines the advantages of ensemble learning in random forests with the characteristics of the Cox proportional hazards model. The following adjustable hyperparameter space was set:

Number of features considered per tree (mtry): 2-20

Number of trees (n_tree): 100-500

Minimum observations in leaf nodes (leaf_min_obs): 5-20

Minimum observations for splitting (split_min_obs): 10-30

Sample sampling proportion (sample_fraction): 0.5-0.8

Control type (control_type): "fast" or "cph"

Fixed parameters include: feature importance evaluation using permutation method (permute), splitting rule using logrank test, and OOB (out-of-bag) prediction type set to survival probability prediction.

Evaluation Metrics

A multi-dimensional evaluation system was established: Primary optimization metric: Harrell's C-index. Secondary metrics: Time-dependent area under the ROC curve (1-year, 3-year, 5-year)

Parameter Optimization Process

A random search strategy (random_search) was used for hyperparameter optimization, with 50 evaluation iterations set. Model performance was evaluated via stratified 5-fold cross-validation, using the C-index as the objective function for optimization. The random search strategy efficiently explores the given parameter space, balancing computational cost and optimization effect.

Model Validation

After determining the optimal parameter combination, the final model was reconstructed using all training data, and its stability was evaluated through stratified 5-fold cross-validation. C-index values of each fold were recorded, and average performance metrics were calculated.

Optimal Parameter Combination: After systematic optimization, the following best parameter configuration was obtained: Number of trees: 358; Number of features considered per tree: 13; Sample sampling proportion: 0.597; Control type: cph; Minimum observations in leaf nodes: 15; Minimum observations for splitting: 16.

Model Performance: The final model showed excellent discriminative ability: Average C-index: 0.971; AUC (1-year, 3-year, 5-year): 0.966, 0.972, 0.926 (training set); 0.796, 0.770, 0.759 (validation set); 0.755, 0.741, 0.760 (Test Set 1); 0.716, 0.702, 0.741 (Test Set 2).

**3. Bart**

Data Preparation

This study uses retrospective cohort data, importing datasets from CSV-format files. Z-score was used to standardize radiomics parameters; ComBat was used to reduce the batch effect of parameters. Data integrity and the rationality of event (status) proportion were checked using the summary() and table() functions. For constructing survival analysis tasks, the "time" variable was used as survival time, and the "status" variable as the event indicator, with a survival task object under the mlr3 framework created to lay the foundation for subsequent model training.

Model Selection and Parameter Optimization

The Bayesian Additive Regression Trees (Bart) survival model was selected. Based on the Bayesian framework, this model integrates multiple regression trees, enabling it to capture complex nonlinear relationships and interaction effects between features, making it suitable for survival analysis scenarios. The adjustable hyperparameter space was set: Number of trees (ntree): 50-200 (controls the complexity of the ensemble model). Prior control parameter (k): 1-3 (adjusts the prior distribution of leaf node effects). Tree depth parameter (power): 1-2 (controls the complexity decay of tree structure). Tree prior base probability (base): 0.8-0.95 (affects the prior probability of tree splitting). Fixed parameters include: number of MCMC posterior samples (ndpost=1000), iteration retention interval (keepevery=1), and initial burn-in iterations (nskip=100), to balance model stability and computational efficiency.

Evaluation Metrics

A multi-dimensional model evaluation system was established: Primary optimization metric: Harrell's C-index (surv.cindex), which comprehensively evaluates the discriminative ability of the model. Secondary metrics: Time-dependent area under the ROC curve, with prediction performance calculated for 1-year, 3-year, and 5-year respectively, reflecting risk differentiation ability at different time points.

Parameter Optimization Process

A random search strategy (random_search) was used for hyperparameter optimization, with 30 evaluation iterations set to balance exploration efficiency and computational cost. Model performance was evaluated via stratified 5-fold cross-validation (rsmp("cv", folds=5)), using the C-index as the core objective function for optimization. Parallel computing (the future package, multisession mode) was used to accelerate the tuning process, utilizing all available cores except one to improve computational efficiency.

Model Validation

After determining the optimal parameter combination, the final model (final_learner) was reconstructed using all training data, and its stability was evaluated through stratified 5-fold cross-validation. C-index values of each fold were recorded, and average performance was calculated. The generalization ability of the model was further verified using the validation set, Test Set 1, and Test Set 2. The C-index of the validation set and AUC values for 1-year, 3-year, and 5-year were calculated and compared with the cross-validation results of the training set.

Optimal Parameter Combination and Model Performance: The best configuration obtained through systematic optimization: ntree=175; k=2.3; power=1.8; base=0.88; MCMC settings: ndpost=1000 for stable sampling.

The final model showed excellent discriminative ability: Average C-index: 0.921; AUC (1-year, 3-year, 5-year): 0.956, 0.936, 0.904 (training set); 0.815, 0.780, 0.807 (validation set); 0.752, 0.694, 0.790 (Test Set 1); 0.760, 0.678, 0.722 (Test Set 2).

**4. DeepHit**

Data Preparation

This study adopts a retrospective cohort design, importing clinical datasets from CSV-format files. All radiomics parameters were standardized using Z-score; ComBat was used to reduce the batch effect of parameters. For constructing survival analysis tasks, the "time" variable was used as survival time, and the "status" variable as the event indicator, with a survival task object task_train under the mlr3 framework created.

Model Selection and Parameter Optimization

The DeepHit survival model was selected, which directly models the discrete-time survival distribution through a neural network and is suitable for handling competing risks and censored data. The following adjustable hyperparameter space was set: Network structure parameters: Hidden layer structures (num_nodes) were set to 3 candidate schemes (c(32,32), c(64,64), c(128,64)); dropout rate (dropout) range: 0.1-0.5; whether to enable batch normalization (batch_norm) was set as a binary choice. Training parameters: Number of training epochs (epochs): 50-150; batch size (batch_size): 32-256; learning rate (learning_rate): 1e-4 to 1e-2. Loss function parameters: Both the censoring loss weight (alpha) and ranking loss smoothing parameter (sigma) in the joint loss were set to 0.1-0.5. Fixed parameters include: enabling early stopping mechanism (early_stopping = TRUE), patience value of 10 epochs, Adam optimizer, and CPU training device.

Evaluation Metrics

A multi-dimensional evaluation system was established: Primary optimization metric: Harrell's C-index (surv.cindex), which comprehensively evaluates the overall discriminative ability of the model. Secondary metrics: Time-dependent area under the ROC curve (1-year, 3-year, 5-year), calculated via surv.uno_auc, reflecting risk differentiation ability at different time points.

Parameter Optimization Process

A Bayesian optimization (mbo) strategy was used for hyperparameter tuning, with stratified 5-fold cross-validation (rsmp("cv", folds=5)) and 40 evaluation iterations set. Parallel computing was implemented using the future package (utilizing all available cores except one) to improve efficiency. Initial design points were generated with 10 random samples via generate_design_random for initializing the surrogate model. The optimization process used the C-index as the objective function to balance exploration and exploitation, ultimately outputting the optimal parameter combination.

Model Validation

Final model training: The model final_learner was reconstructed using optimal parameters and trained on the complete training set. Cross-validation evaluation: Average C-index and time-dependent AUC were calculated via stratified 5-fold cross-validation, with performance metrics of each fold recorded to evaluate stability. Independent validation set testing: An independent validation set task_test was imported, and the C-index as well as 1-year, 3-year, and 5-year AUC were calculated, compared with training set results to verify generalization ability. Visualization analysis: Survival curves were plotted to intuitively display model prediction results, and optimization progress was monitored via tuning process visualization.

Optimal Parameter Combination and Model Performance: The following best configuration was obtained through systematic optimization: Network structure: num_nodes = c(64, 64); regularization: dropout = 0.3; batch_norm = TRUE; training parameters: epochs = 120; batch_size = 128; learning_rate = 5e-4; loss function: alpha = 0.3; sigma = 0.4.

Model performance: Average C-index = 0.892; AUC (1-year, 3-year, 5-year): 0.985, 0.995, 0.956 (training set); 0.734, 0.660, 0.689 (validation set); 0.680, 0.761, 0.697 (Test Set 1); 0.705, 0.695, 0.714 (Test Set 2).

**5. DeepSurv**

Data Preparation

This study adopts a retrospective cohort design, importing clinical datasets from CSV-format files. Z-score was used to standardize radiomics parameters; ComBat was used to reduce the batch effect of parameters. Data reliability was ensured through a series of quality control steps: the summary() function was used to analyze the distribution characteristics of survival time (the time variable), and the table() function was used to examine the distribution proportion of event status (the status variable, 0 = censored, 1 = event). For constructing survival analysis tasks, "time" was used as a continuous survival time variable and "status" as the event indicator. A survival task object task_train under the mlr3 framework was created using the as_task_surv() function.

Model Selection and Parameter Optimization

The DeepSurv survival model was selected, which models the Cox proportional hazards function based on a deep neural network architecture. It can capture complex nonlinear relationships and interaction effects between features, making it suitable for survival analysis scenarios with censored data. The following adjustable hyperparameter space was set:

Network structure parameters: Hidden layer structures (num_nodes) were set to 4 candidate schemes, including single hidden layer (32 nodes, 64 nodes) and double hidden layers (32+32 nodes, 64+32 nodes), controlling network complexity; Dropout rate (dropout) range: 0.1-0.5, used to prevent overfitting; Whether to enable batch normalization (batch_norm) was set as a binary choice (TRUE/FALSE), optimizing network training stability.

Training parameters: Number of training epochs (epochs): 50-200, controlling the number of model iterations; Batch size (batch_size): 32-128, balancing training efficiency and parameter update stability; Learning rate (learning_rate): 1e-4 to 1e-2, adjusting parameter update step size; L2 regularization coefficient (weight_decay): 1e-6 to 1e-3, suppressing overfitting.

Fixed parameters include: enabling early stopping mechanism (early_stopping=TRUE) with a patience value of 15 epochs (patience=15L), terminating training when performance does not improve; using the Adam optimizer (optimizer="adam"); automatically selecting the computing device based on the number of cores to improve training efficiency.

Evaluation Metrics

A multi-dimensional model evaluation system was established to comprehensively assess model performance: Primary optimization metric: Harrell's C-index (surv.cindex), which comprehensively measures the model's ability to discriminate survival time rankings; a value closer to 1 indicates better discriminative performance. Secondary metrics: Integrated Brier score (surv.graf), evaluating the calibration between predicted survival probabilities and actual outcomes; time-dependent area under the ROC curve (surv.uno_auc), with AUC values calculated for 1-year, 3-year, and 5-year respectively, reflecting the model's risk differentiation ability at key time points.

Parameter Optimization Process

A Bayesian optimization (mbo) strategy was used for efficient hyperparameter tuning, with the specific process as follows: Cross-validation setup: stratified 5-fold cross-validation (rsmp("cv", folds=5)) was used to evaluate parameter combination performance, reducing the impact of data partitioning bias on results. Parallel computing acceleration: The future package was used to enable multi-session parallel computing (plan(multisession, workers=availableCores()-1)), utilizing all available cores except one to significantly improve tuning efficiency. Optimizer configuration: The initial design initialized the surrogate model with 10 random samples (initial_design=10), using Gaussian process regression (regr.km) as the surrogate model and expected improvement (acqf("ei")) as the acquisition function to balance exploration and exploitation of the parameter space. Tuning termination condition: The total number of evaluations was set to 50 (term_evals=50), with the C-index as the core optimization objective function, ultimately outputting the optimal parameter combination.

Model Validation

After determining the optimal parameters, multi-level validation was performed to ensure model reliability and generalization ability: Final model training: final_model was initialized based on the optimal parameter combination (best_params), retrained using the complete training set (task_train), and model weights were saved (saveRDS) for subsequent analysis. Cross-validation evaluation: stratified 5-fold cross-validation (resample) was used to calculate the average C-index, integrated Brier score, and 1-year, 3-year, 5-year AUC of the training set. External validation set testing: An independent validation set (val_data) was imported, standardized using the same method to create task_val, and various performance metrics of the validation set were calculated and compared with training set results to verify generalization ability.

The optimal parameter configuration obtained through systematic optimization: Network structure: num_nodes=c(64, 32) (double hidden layers); batch_norm=TRUE; Regularization parameters: dropout=0.3; weight_decay=5e-5; Training parameters: epochs=150; batch_size=64; learning_rate=3e-4. Supplementary optimal configuration: Network structure: double hidden layers nodes; regularization parameters: dropout=0.25, batch_norm=TRUE; training parameters: epochs=180, batch_size=64; optimization settings: learning_rate=3.2e-4, weight_decay=5e-5.

Model performance: Average C-index = 0.876; Integrated Brier score = 0.182; AUC (1-year, 3-year, 5-year): 0.998, 0.987, 0.980 (training set); 0.842, 0.865, 0.826 (validation set); 0.826, 0.811, 0.760 (Test Set 1); 0.772, 0.655, 0.705 (Test Set 2). The model showed good discriminative and calibration abilities in the training set, with slightly decreased but stable performance in the validation set, indicating a certain generalization ability.

**6. DNNSurv**

Data Preparation

This study uses retrospective cohort data, importing datasets from CSV-format files. Z-score was used to standardize radiomics parameters; ComBat was used to reduce the batch effect of parameters. Data integrity and the rationality of event (status) proportion were checked using the summary() and table() functions. For constructing survival analysis tasks, the "time" variable was used as survival time, and the "status" variable as the event indicator, with a survival task object under the mlr3 framework created to lay the foundation for subsequent model training.

Model Selection and Parameter Optimization

The DNNSurv survival model was selected, which directly models the survival function based on a deep neural network and is suitable for handling high-dimensional nonlinear feature relationships. The adjustable hyperparameter space was set as follows: Network structure parameters: Hidden layer structures were set to 4 candidate schemes (c(64,64,32), c(128,64), c(256,128,64), c(32,16)); Regularization parameters: Dropout rate range: 0.1-0.5; activation function options: relu/tanh/sigmoid; Training parameters: Number of training epochs: 50-300 (search on log scale); batch size: 16/32/64/128; learning rate: 1e-4 to 1e-2 (search on log scale); Loss functions: Cox loss and MTLR loss commonly used in survival analysis. Fixed parameters include: Early stopping mechanism: enabled with patience=20; optimizer: Adam optimizer; device selection: automatic detection of GPU/CPU.

Evaluation Metrics

A multi-dimensional model evaluation system was established: Primary optimization metric: Harrell's C-index (surv.cindex), which comprehensively evaluates the overall discriminative ability of the model. Secondary metrics: Time-dependent area under the ROC curve: prediction performance calculated for 1-year, 3-year, and 5-year respectively (surv.uno_auc); Brier score (surv.brier): evaluates the calibration between predicted probabilities and actual survival status.

Parameter Optimization Process

A Bayesian optimization (mbo) strategy was used for hyperparameter tuning: Surrogate model: Random forest (regr.ranger) was used as the surrogate model; Acquisition function: Expected Improvement (EI) algorithm to balance exploration and exploitation; Cross-validation: stratified 5-fold cross-validation (rsmp("cv", folds=5)); Parallel computing: The future package was used to implement multi-threaded parallelism, retaining 2 cores to avoid system overload; Initial design: 15 random initial samples were generated to accelerate optimization.

The optimization process included: Defining the search space: containing 10 adjustable parameters such as network structure, regularization, and training parameters; executing 50 evaluation iterations: each iteration included stratified 5-fold cross-validation; warm start support: allowing continuation of optimization from saved intermediate results; visualization monitoring: real-time tracking of the optimization process via autoplot.

Model Validation

Final model training: The model final_learner was reconstructed using optimal parameters and trained on the complete training set. Cross-validation evaluation: Average C-index and time-dependent AUC were calculated via stratified 5-fold cross-validation.

Optimal Parameter Combination and Model Performance: The following best configuration was obtained through systematic optimization: Network structure: c(128,64); regularization: dropout=0.3, activation function=relu; training parameters: epochs=200, batch_size=64, learning_rate=2e-4; loss function: mtlr.

Model performance: Average C-index = 0.925; AUC (1-year, 3-year, 5-year): 0.984, 0.990, 0.962 (training set); 0.697, 0.767, 0.829 (validation set); 0.777, 0.676, 0.821 (Test Set 1); 0.825, 0.692, 0.808 (Test Set 2).

**7. Glmnet**

Data Preparation

This study uses retrospective cohort data, importing datasets from CSV-format files. Z-score was used to standardize radiomics parameters; ComBat was used to reduce the batch effect of parameters. The quality of model input was ensured through a strict data preprocessing process: Key variables "time" (survival time) and "status" (event status) were correctly identified. Survival task construction: A survival task object under the mlr3 framework was created with "time" as survival time and "status" as the event indicator, laying the foundation for model training.

Model Selection and Parameter Optimization

The Elastic Net survival model (Cox proportional hazards model based on glmnet) was selected, which balances feature selection and model stability through a mixture of L1 (LASSO) and L2 (ridge regression) regularization. Adjustable parameter space: alpha (Elastic Net mixing parameter): 0 (pure ridge regression) to 1 (pure LASSO), controlling feature sparsity. lambda (regularization strength): Search range on a logarithmic scale (1e-4 to 1), covering scenarios from no penalty to strong shrinkage. Fixed parameters: standardize = TRUE (standardizes predictor variables to ensure coefficient comparability); maxit = 10000 (increases the number of iterations to ensure convergence, avoiding model bias due to insufficient optimization); nlambda = 100 (generates 100 lambda values to fully explore the regularization path).

Evaluation Metrics

A multi-dimensional evaluation system was established to comprehensively measure model performance: Primary optimization metric: Harrell's C-index (surv.cindex), which comprehensively evaluates the model's ability to discriminate event occurrence order. Time-dependent metrics: 1-year, 3-year, and 5-year AUC (surv.uno_auc), reflecting risk differentiation ability at different time points. Brier score (surv.brier), quantifying the deviation between predicted risk and actual events to evaluate prediction accuracy.

Parameter Optimization Process

A nested cross-validation framework was adopted to ensure model stability and parameter generalization: Outer validation: stratified 5-fold cross-validation to evaluate model stability and avoid overfitting. Inner tuning: Bayesian optimization (mbo) dynamically explores the parameter space through a surrogate model (Gaussian process), reducing computational cost by over 50% compared to grid search; stratified 5-fold cross-validation evaluates parameter combinations in inner CV, with 50 iterative searches using the C-index as the objective function. Parallel computing: mlr3's parallel support was used to accelerate the tuning process and improve efficiency.

Model Validation

Final model training: The model was reconstructed using all training data and optimal parameters (alpha, lambda) to ensure parameter estimation fully utilizes data information. Performance evaluation: Cross-validation results report the mean and 95% confidence interval of C-index, AUC, and Brier score to quantify model stability; time-dependent ROC analysis plots 1-5 year AUC curves to intuitively display the model's predictive ability at different time points.

The following best configuration was obtained through systematic optimization: alpha: 0.52 (balances L1/L2 regularization, tending to retain a moderate number of features and mitigate multicollinearity); lambda: 0.0173 (selected via Bayesian optimization in the logarithmic space from 1e-4 to 1, corresponding to moderate regularization strength that avoids overfitting while retaining key predictive information).

Model performance: C-index: 0.786 (95% CI: 0.742-0.830), indicating good overall discriminative ability for event occurrence order. AUC (1-year, 3-year, 5-year): 0.982, 0.996, 0.967 (training set); 0.863, 0.821, 0.772 (validation set); 0.809, 0.742, 0.711 (Test Set 1); 0.796, 0.708, 0.802 (Test Set 2).

**8. GBM**

Data Preparation

This study uses retrospective cohort data, importing datasets from CSV-format files, with the core objective of constructing a survival prediction model based on Gradient Boosting Machine (GBM). The data preprocessing process is as follows: Z-score was used to standardize radiomics parameters; ComBat was used to reduce the batch effect of parameters. Key variable identification: "time" (survival time) and "status" (event status, e.g., occurrence of outcome) were identified as core variables for survival analysis, and a Surv(time, status) object was constructed as the model response variable.

Model Selection and Parameter Optimization

The GBM survival model based on the gbm package was selected, which optimizes the Cox proportional hazards model through a gradient boosting framework (distribution = "coxph"), combining nonlinear fitting ability and adaptability to survival data. Adjustable parameter space: n.trees: Number of ensemble trees (1000, 2000, 3000), controlling model complexity; too few may lead to underfitting, while too many may cause overfitting. shrinkage: Learning rate (0.001, 0.005, 0.01), controlling the contribution intensity of each tree; smaller values require more tree iterations and generally improve generalization ability. interaction.depth: Maximum depth of trees (2, 3, 4), controlling the order of feature interactions; greater depth makes it easier to capture high-order interactions but may lead to overfitting. n.minobsinnode: Minimum number of observations in nodes (5, 10, 15), controlling the stopping condition for tree growth; larger values result in simpler models with stronger anti-overfitting ability. Fixed parameters: cv.folds = 5 (stratified 5-fold cross-validation was used to evaluate parameter performance); verbose = FALSE (turned off training process log output to simplify running information).

Evaluation Metrics

A multi-dimensional indicator system was constructed to comprehensively evaluate model performance, closely aligned with the characteristics of survival analysis: Primary optimization metric: Harrell's C-index (calculated via survConcordance), quantifying the model's ability to discriminate the order of event occurrence time, with a range of [0.5, 1]; closer to 1 indicates stronger discrimination ability. Time-dependent metrics: AUC at 1-year, 3-year, and 5-year time points (calculated via the timeROC package), reflecting the model's ability to distinguish between high-risk and low-risk individuals at specific time points; closer to 1 indicates higher prediction accuracy. Auxiliary analysis: Feature importance, measured by "relative influence (rel.inf)" to assess the contribution of variables to model predictions, used to explain the model's decision logic.

Parameter Optimization Process

A framework combining grid search and cross-validation was adopted to systematically explore the parameter space for optimal configuration: Grid search: All parameter combinations (3×3×3×3=81 types) were generated via expand.grid, iterating through all possible parameter configurations. Cross-validation setup: Inner layer used stratified 5-fold cross-validation (cv.folds = 5), and the stability of each parameter combination was evaluated via cross-validation built into the gbm function. Determination of optimal iteration count: For models trained with each parameter combination, the optimal n.trees (i.e., the number of trees to stop iteration) was determined via the gbm.perf function (based on cross-validation error) to balance model complexity and overfitting risk. Optimal parameter selection: Using the C-index on the training set as the objective function, the parameter combination corresponding to the highest C-index (including the optimal number of iterations) was selected as the parameter configuration for the final model.

Model Validation and Result Output

Final model training: The GBM model was retrained using all training data and optimal parameters to ensure parameter estimation fully utilized training information, and the optimal number of trees was reconfirmed via gbm.perf. Multi-dataset evaluation: Risk scores were predicted on the training set, test set, and validation set respectively. The 1-year, 3-year, and 5-year AUC for each dataset were calculated via the evaluate_model function, and time-dependent ROC curves were plotted to intuitively display the model's generalization ability across different data partitions.

The following optimal configuration was obtained through systematic optimization: n.trees=2000 (total number of ensemble trees, controlling basic model complexity); shrinkage=0.01 (learning rate, balancing iteration efficiency and model convergence, a medium-strength value within the search range); interaction.depth=2 (maximum tree depth, limited to 2 layers, indicating the model focuses on capturing low-order feature interactions to reduce overfitting risk); n.minobsinnode=5 (minimum observations in nodes, controlling tree growth granularity, allowing nodes to maintain a certain degree of subdivision); best_iter (optimal iteration count)=1473 (optimal number of trees determined via cross-validation; only the first 1473 trees are needed to achieve the best performance in practice). Core model configuration: Based on the Cox proportional hazards framework (distribution = "coxph"), retrained using all training data to ensure sufficient parameter estimation.

Model performance: AUC (1-year, 3-year, 5-year): 0.997, 0.983, 0.991 (training set); 0.788, 0.742, 0.720 (validation set); 0.633, 0.750, 0.736 (Test Set 1); 0.716, 0.692, 0.729 (Test Set 2).

**9. RSF**

Data Preparation

This study uses retrospective cohort data, importing the training set and external validation set from CSV-format files, with the core objective of constructing a survival prediction model based on Random Survival Forest (RSF). The data preprocessing process is as follows: Z-score was used to standardize radiomics parameters; ComBat was used to reduce the batch effect of parameters. Key variable identification: "time" (survival time) and "status" (event status, e.g., occurrence of outcome) were identified as core variables for survival analysis. The model response variable was constructed via Surv(time, status), with all covariates included as predictive features.

Model Selection and Parameter Optimization

The RSF model was implemented using the rfsrc function from the randomForestSRC package. It improves prediction stability by integrating multiple survival trees (with "logrank" as the splitting criterion) and is suitable for handling nonlinear relationships and interaction effects in survival data. Adjustable parameter space: ntree: Number of ensemble trees (grid in code: 500, 1000, 1500), controlling model complexity; too few may lead to underfitting, while too many may increase computational cost with diminishing marginal benefits. mtry: Number of features randomly selected for each node split (set in code based on total feature count as √(p), p/2, p/3, where p is the number of covariates), balancing randomness and feature utilization efficiency, and affecting model overfitting risk. nodesize: Minimum sample size per node (grid in code: 3, 5, 10), controlling tree growth granularity; larger values result in simpler models (stronger anti-overfitting ability), while smaller values may capture more details but are prone to overfitting. Fixed parameters: splitrule = "logrank" (log-rank test was used as the node splitting criterion to maximize survival differences between child nodes, aligning with survival analysis objectives); importance = TRUE (calculates variable importance for model interpretation). Cross-validation folds: 5 folds (for parameter evaluation stability).

Evaluation Metrics

A multi-dimensional indicator system was constructed to comprehensively evaluate model performance based on the characteristics of survival data: Primary optimization metric: Time-dependent AUC (calculated via the timeROC package), reflecting the model's ability to distinguish between high-risk and low-risk individuals at specific time points (1-year, 3-year, 5-year in code); closer to 1 indicates stronger discrimination ability. Auxiliary evaluation metrics: Log-rank test p-value (survival curve differences between high- and low-risk groups (divided by the median risk score) were compared via survdiff; a smaller p-value indicates more significant statistical significance of risk grouping); variable importance (based on the model's built-in importance score (final_model$importance), measuring the contribution of each covariate to survival prediction for explaining the model's decision logic).

Parameter Optimization Process

A framework combining grid search and cross-validation was adopted to systematically explore the parameter space for optimal configuration: Grid search: All combinations of ntree, mtry, and nodesize were iterated through nested loops (3×3×3=27 types in code), covering key values in the parameter space. Cross-validation setup: Inner layer used stratified 5-fold cross-validation. For each parameter combination, the model was repeatedly trained on 5 subsets of the sub-training set and evaluated on validation subsets, with the average AUC calculated as the performance metric. Optimal parameter selection: Using the average cross-validation AUC as the objective function, the parameter combination corresponding to the maximum AUC (best_params) was selected to balance model performance and generalization ability.

Model Validation and Result Output

Final model training: The RSF model (final_model) was retrained using the entire training set and optimal parameters to ensure parameter estimation fully utilized training information.Multi-dataset evaluation: Risk scores were predicted on the sub-training set, test set, and external validation set via the evaluate_model function. The 1-year, 3-year, and 5-year AUC for each dataset were calculated, and time-dependent ROC curves were plotted to intuitively display the model's generalization ability across different data partitions.

The following optimal configuration was obtained through systematic optimization: ntree=1000 (model performance improved significantly compared to 500 trees; increasing to 1500 trees showed no significant AUC improvement but increased computation time by 50%, so 1000 was chosen to balance performance and efficiency); mtry=5 (based on the total number of features in the training set, three values were tested: √28≈5.29, 28/2=14, 28/3=9.33; finally, 5 (close to √18 and rounded) achieved the highest cross-validation AUC, indicating it better balances feature randomness and information utilization); nodesize=5 performed best, balancing fitting accuracy and generalization ability. splitrule="logrank" was fixed as the log-rank test, as literature indicates its sensitivity to event differences in survival data is significantly higher than other criteria such as "extratrees", making it the standard splitting strategy for RSF models.

Model performance: AUC (1-year, 3-year, 5-year): 0.955, 0.962, 0.960 (training set); 0.836, 0.851, 0.790 (validation set); 0.798, 0.832, 0.767 (Test Set 1); 0.718, 0.806, 0.731 (Test Set 2).

**10. SVM**

Data Preparation

This study uses retrospective cohort data to construct an SVM survival prediction model. The data sources and preprocessing are as follows: Z-score was used to standardize radiomics parameters; ComBat was used to reduce the batch effect of parameters. The training set (data) and external validation/test sets were loaded using the read.csv function, with data in CSV format containing core variables for survival analysis and predictive features. Core variable identification: "time" (survival time) and "status" (event status, e.g., occurrence of outcome) were identified as core variables for the survival model. The model response variable was constructed via Surv(time, status), with other variables included as predictive features.

Model Selection and Parameter Optimization

The SVM survival model was implemented using the survivalsvm package. It maps survival data to a high-dimensional space via kernel functions and captures nonlinear relationships between survival time, event status, and features using support vector machine principles, making it suitable for handling complex covariate interaction effects. Model types and kernel functions: Model types: Three survival SVM types were iterated to adapt to different characteristics of survival data: "regression": Survival SVM based on a regression framework, directly modeling relationships between survival time and features; "vanbelle1": Survival SVM based on the Van Belle method, optimizing discriminative ability for event occurrence probability; "hybrid": Hybrid framework survival SVM, integrating regression and classification ideas to improve prediction robustness. Adjustable parameter space: Core adjustable parameters are gamma and mu, with the parameter grid set as gamma.mu_range = c(0.01, 0.1, 1, 10). Their specific roles are as follows: gamma: Kernel function parameter (especially for RBF kernel), controlling the complexity of feature space mapping; larger values make the model more prone to overfitting, while smaller values may lead to underfitting; mu: Regularization parameter, balancing model fitting error and complexity; too small values may cause overfitting, while too large values may lead to underfitting. Fixed parameters: opt.meth = "quadprog" (quadratic programming algorithm was used to solve optimization problems, ensuring stability of parameter estimation); diff.meth = "makediff1" (first-order difference method was used to handle time dependence of survival data, consistent with the theoretical framework of survival SVM); cross-validation folds: 5 folds, used to evaluate parameter stability and generalization ability.

Evaluation Metrics

An evaluation system centered on time-dependent AUC was constructed to adapt to the time dynamics of survival data: Primary metric: Time-dependent AUC (calculated via the timeROC package), reflecting the model's ability to distinguish between high-risk and low-risk individuals at specific time points (1-year, 3-year, 5-year, corresponding to times = c(12, 36, 60) in code); closer to 1 indicates stronger discrimination ability. Metric calculation logic: Risk scores predicted by the model were extracted via the evaluate_model function. AUC at each time point was calculated by combining survival time and event status, with the average AUC used as the objective function for parameter optimization.

Parameter Optimization Process

A framework combining grid search and cross-validation was adopted to systematically explore the parameter space for optimal configuration, as follows: Cross-validation setup: stratified 5-fold cross-validation was constructed via the createFolds function, dividing the sub-training set into training and validation subsets to ensure robustness of parameter evaluation. Grid search iteration: All parameter combinations were iterated through nested loops: the outer layer iterated through model types (3 types, regression, vanbelle1, and hybrid) and kernel functions (3 types, linear_kernel, poly_kernel, and rbf_kernel), totaling 9 model frameworks; the inner layer iterated through 4×4=16 combinations of gamma and mu, covering key values in the parameter space. Parallel acceleration: Parallel computing was implemented using the doParallel package, calling detectCores() - 1 cores to simultaneously run training and evaluation of different parameter combinations, significantly improving optimization efficiency. Optimal parameter selection: Using the average cross-validation AUC as the objective function, the parameter combination corresponding to the maximum AUC (best_params) was selected to balance model performance and generalization ability. AUC results of all parameter combinations (all_results) were recorded for subsequent analysis.

Model Validation and Result Output

The SVM survival model (best_model) was retrained using the entire training set and optimal parameters to ensure parameter estimation fully utilized training information. The model's generalization ability was validated on 3 datasets via the evaluate_model function: the training set evaluated model fitting effect; the validation and test sets evaluated robustness in independent cohorts. Time-dependent ROC curves for each dataset were plotted via the plot_roc function, with 1-year, 3-year, and 5-year AUC values labeled to intuitively display the model's discriminative ability.

The following optimal configuration was obtained through systematic optimization: Model type: hybrid (integrating advantages of regression and ranking); kernel function: rbf_kernel (performing best in nonlinear survival data); gamma (γ): 0.12 (balancing model complexity and generalization ability); mu (μ): 1.2; diff.meth: makediff1; opt.meth: quadprog. The parameter grid was dynamically expanded during the optimization process. The initially set parameter grid for gamma and mu (0.01, 0.1, 1, 10) only covered key reference values. To capture more refined optimal parameter combinations, based on the trends observed in the initial search results, the parameter range was expanded to 0.01–10, and a denser search grid was constructed with a step size of 0.01. This adjustment aimed to address the complex and sensitive nature of nonlinear relationships in survival data, enhancing the ability to capture optimal solutions by refining parameter intervals. The finally determined gamma=0.12 and mu=1.2 were selected from the expanded grid through 5-fold cross-validation, as they yielded the highest average AUC. This approach not only avoided missing optimal values due to the coarseness of the initial grid but also achieved improved precision in parameter optimization within a controllable computational cost range.

Model performance: AUC (1-year, 3-year, 5-year): 0.912, 0.946, 0.881 (training set); 0.778, 0.815, 0.801 (validation set); 0.700, 0.823, 0.762 (Test Set 1); 0.650, 0.733, 0.796 (Test Set 2).

## Figures


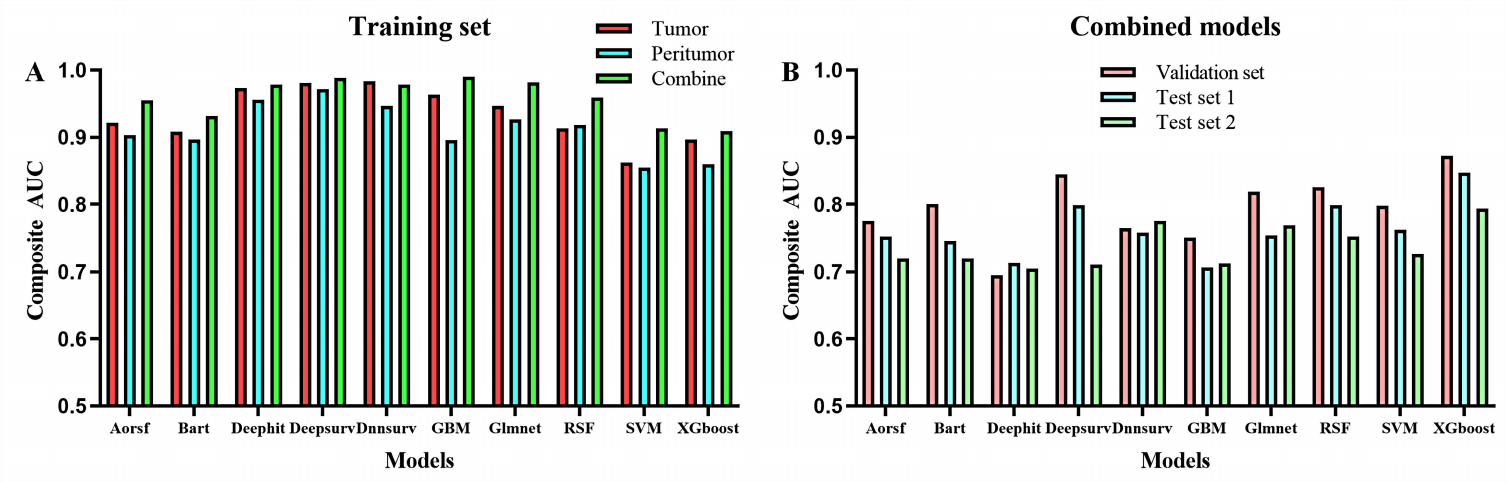


Figure S1 Comparison of predictive efficacy of different Radiomics models. Figure A shows the comparison of the combined AUC of the 10 Radiomics models in predicting endometrial cancer prognosis in the training set; Figure B shows the comparison of the combined AUC of the 10 combined models in the validation set, test set 1, and test set 2. Composite AUC = (1-year AUC + 3-year AUC + 5-year AUC)/3*100%.


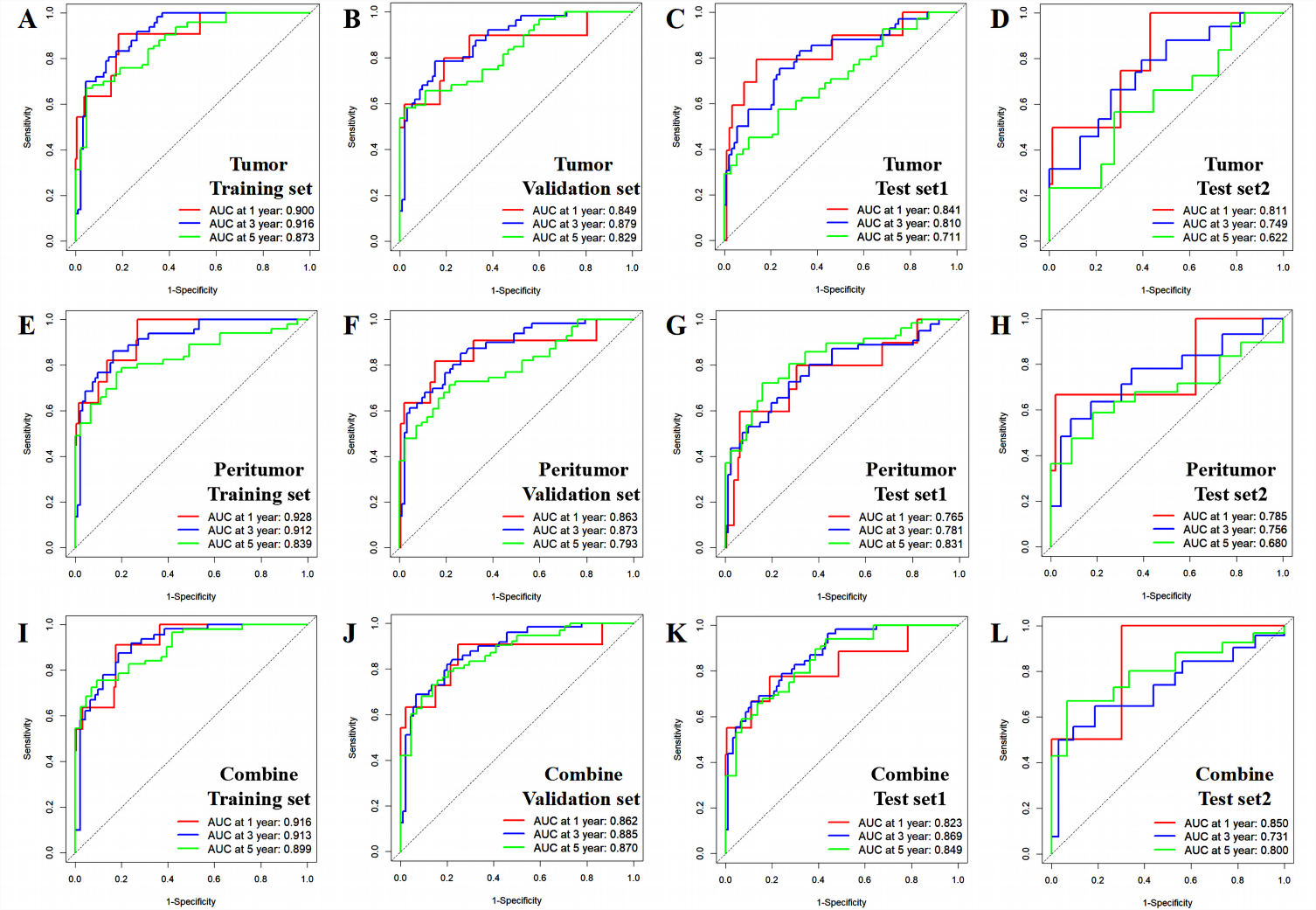


Figure S2 ROC curves of different Radiomics models. Figures A, B, C, and D show the ROC curves of the tumor-based Radiomics model in the training set, validation set, test set 1 and test set 2; Figures E, F, G, and H show the ROC curves of the peritumor-based Radiomics model in the training set, validation set, test set 1 and test set 2; Figures I, J, K, and L show the ROC curves of the combined tumor and peritumor-based Radiomics model in the training set, validation set, test set 1 and test set 2.


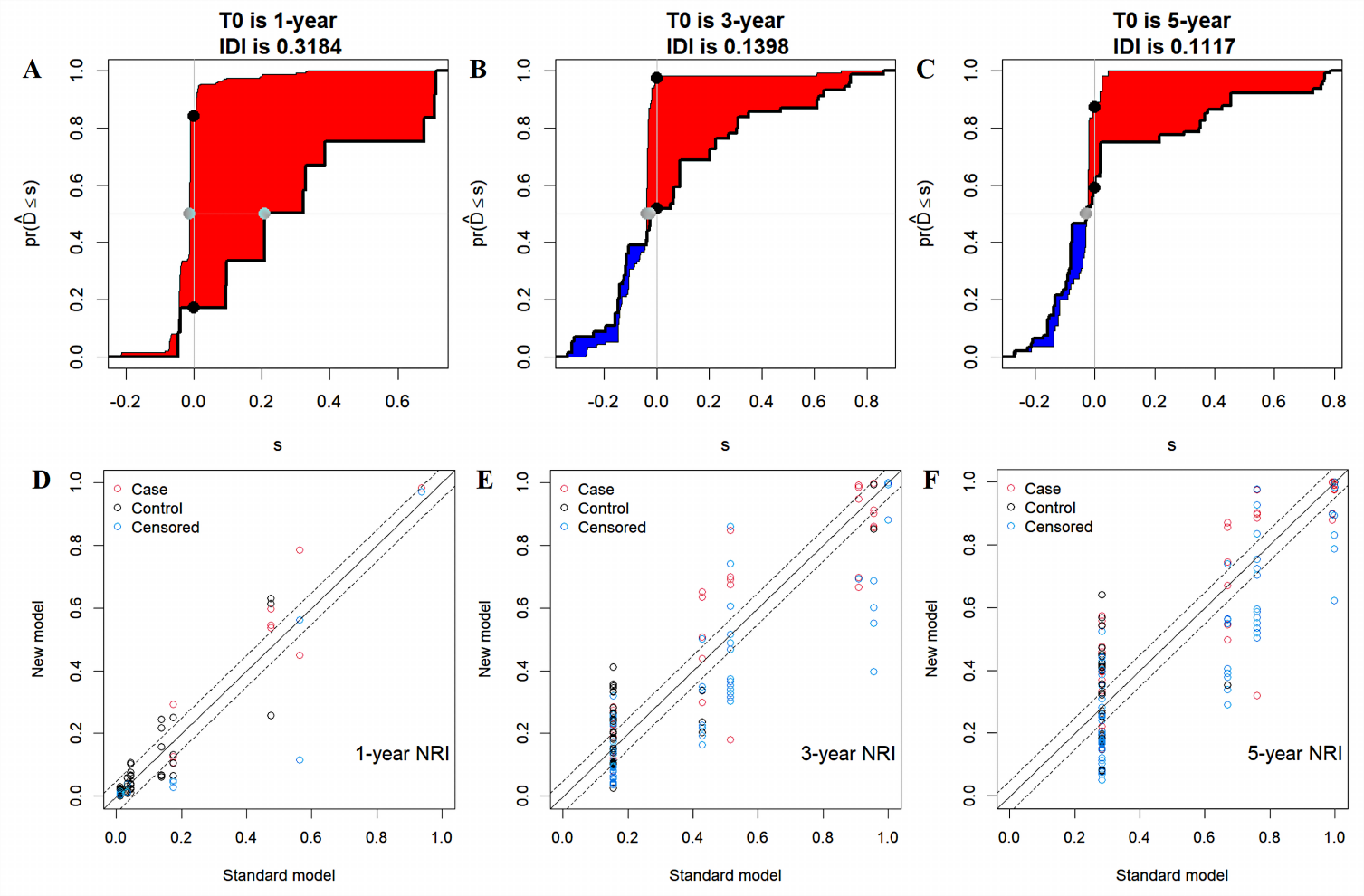


Figure S3 Incremental value of Radscore for clinical metrics. Figures A, B, and C show the 1-, 3-, and 5-year IDI plots of Radscore of the model, and the area in red minus the area in blue represents the IDI value, and the results suggest that Radscore is comprehensive and significant in improving the clinical predictive indexes in HCC prognosis prediction; Figures D, E, and F show the 1-, 3-, and 5-year NRI plots of Radscore in the model. The dashed line is the hierarchical matrix of the model, and the greater distinction between red and blue color represents the higher hierarchical efficacy of the model.


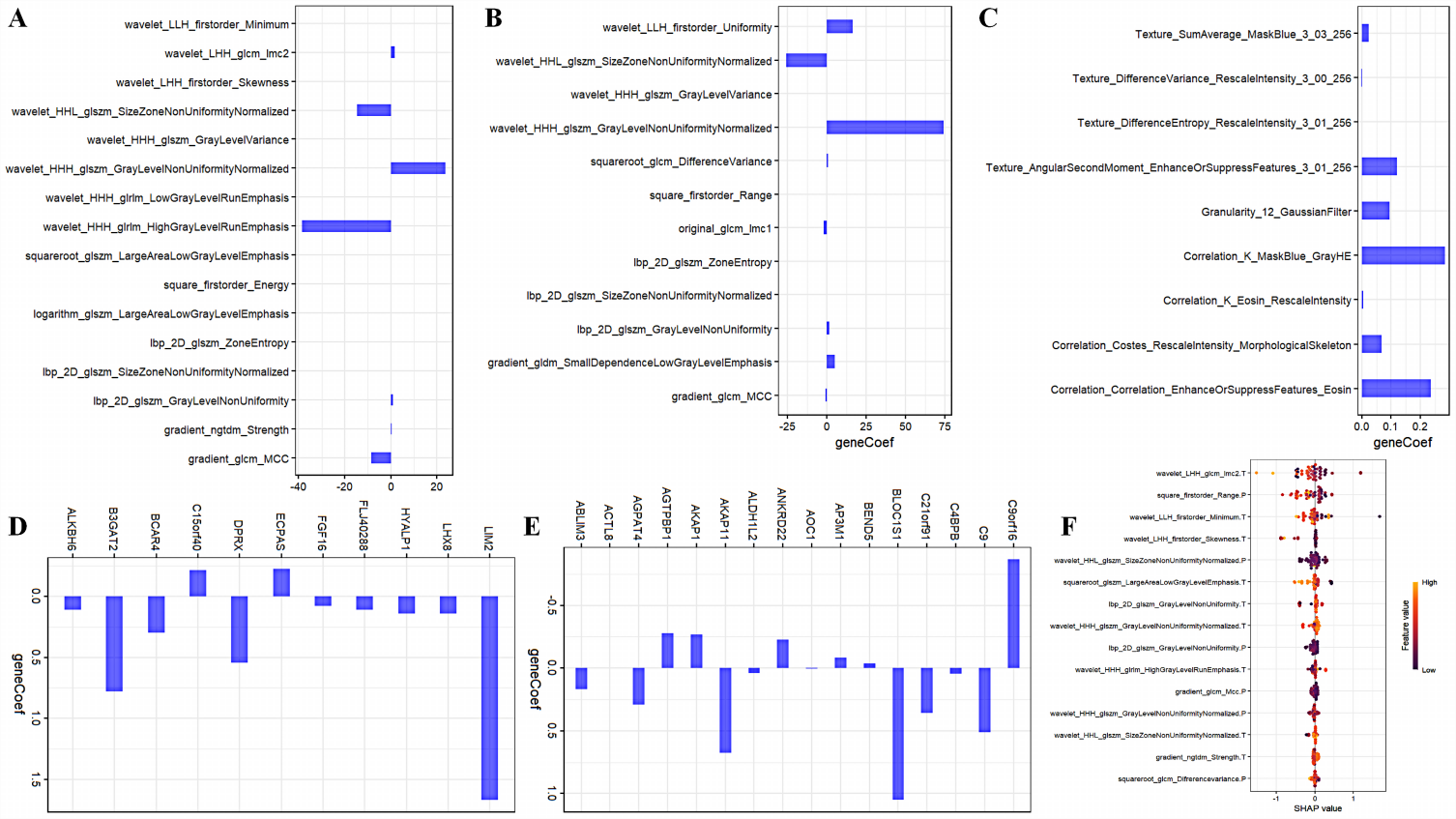


Figure S4 Composition of each omics model. Figures A and B show the tumor, and peritumor Radiomics parameters after LASSO screening, respectively (these parameters were used for subsequent Radiomics model construction); Figure C shows the parameter compositions and weights of the Pathomics model; Figure D shows the parameter compositions and weights of the transcriptomics model; Figure E shows the parameter compositions and weights of the proteomics model; and Figure F shows the SHAP feature ordering of the Radiomics model which is used to reveal the weights and values of each parameter in the black box model.

Figure S5 Calibration curves of each model. Figure A shows the calibration curves of the radiomics model in the training set, validation set, Test Set 1, and Test Set 2; Figure B presents the calibration curves of the nomogram model (radiomics + clinical indicators) in the training set, validation set, and Test Set 1; Figure C displays the calibration curve of the multi-omics model in Test Set 2.


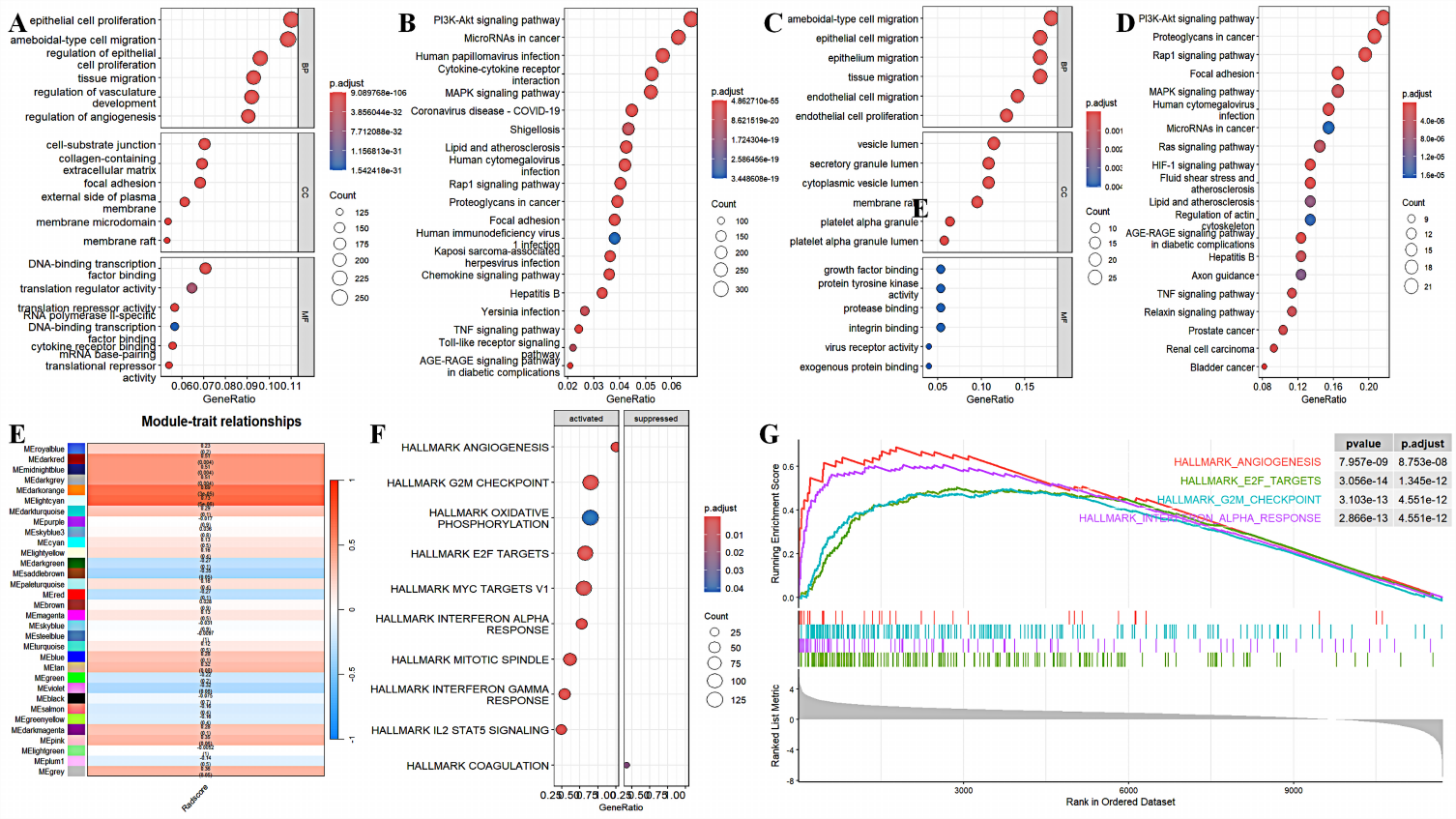


Figure S6 Enrichment analysis. Figure A shows GO enrichment of transcriptome-differentiated genes, suggesting enrichment in pathways such as epithelial cell proliferation and cell migration; Figure B shows KEGG enrichment of transcriptome-differentiated genes, suggesting enrichment in PI3K and TNF pathways; Figure C shows GO enrichment of proteome-differentiated genes, suggesting enrichment in endothelial cell migration and proliferation pathways; Figure D shows KEGG enrichment of proteome-differentiated genes, suggesting enrichment in the PI3K and HIF pathways; Figure E shows WGCNA clustering analysis, showing that MElightcyan cluster genes have the strongest correlation with Radscore; Figure F and G shows GSEA enrichment analysis of MElightcyan cluster genes, suggesting that the enrichment is in angiogenic and cell cycle pathways.


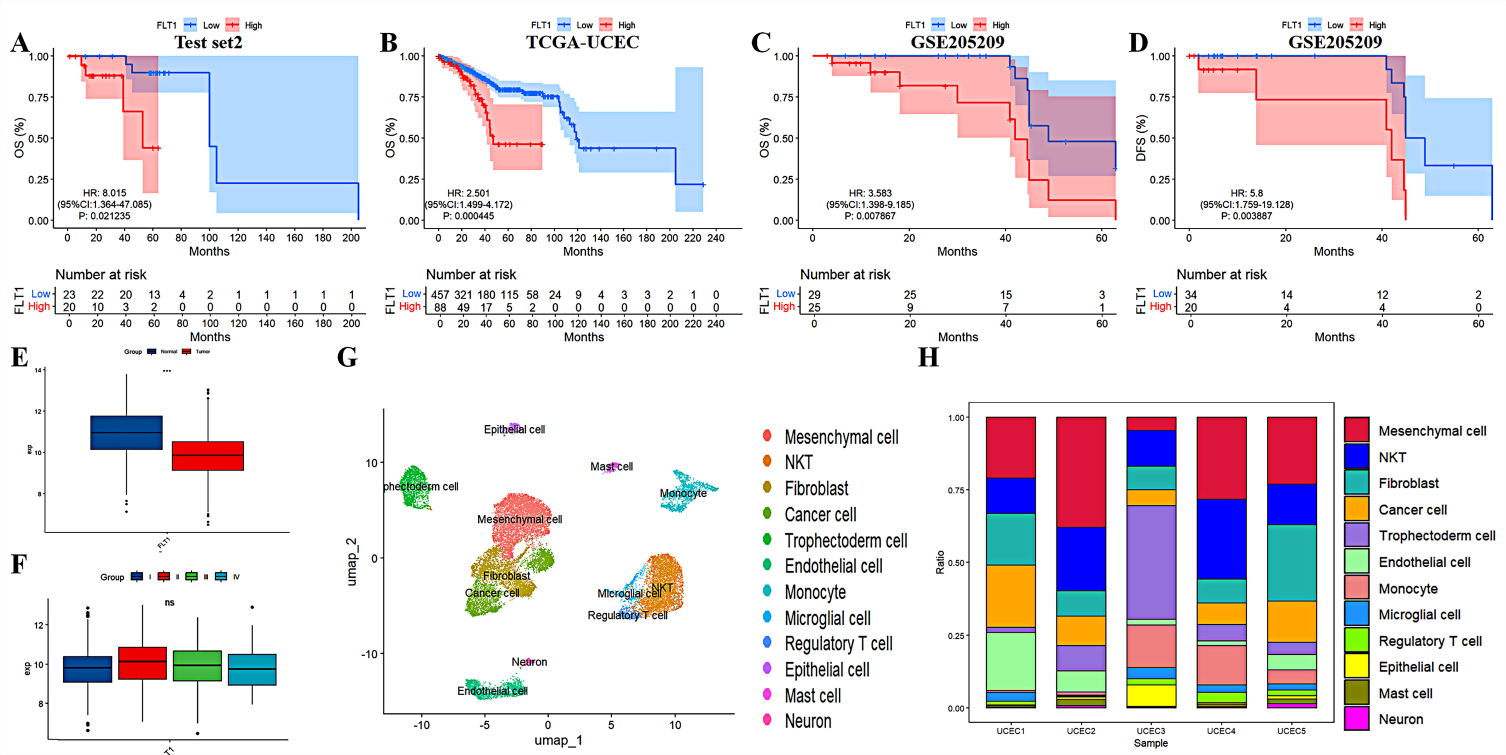


Figure S7  Function and expression of FLT1. Figure A shows the K-M curves of FLT1 in test set 2 (43 patients) for predicting OS; Figure B shows the K-M curves of FLT1 in TCGA-UCEC (545 patients) for predicting OS; Figures C and D show the K-M curves of FLT1 in GSE205209 (54 patients) for predicting OS and DFS; Figure E shows the distribution of FLT1 in cancer and normal tissues, suggesting that it is lowly expressed in endometrial cancers; Figure F shows the correlation analysis between FLT1 and FIGO stage, suggesting no significant correlation; Figure G shows the clustering of 5 endometrial cancer single-cell sequencing in the GSE173682 dataset; Figure H shows the cell ratios between the 5 samples.


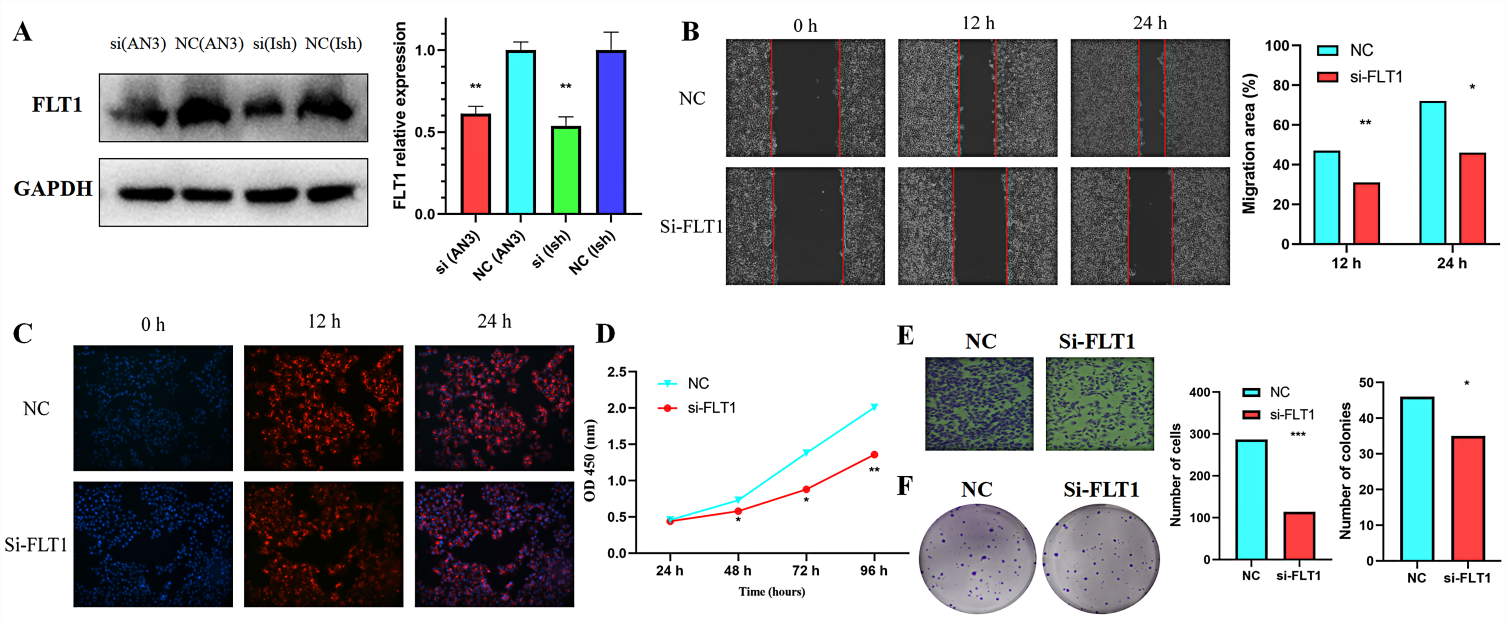


Figure S8  Functional validation of FLT1. Figure A shows the WB and PCR validation of FLT1 knockdown in Ishikawa and AN3CA cell lines; Figure B shows the scratch assay, in which endometrial cancer cells (AN3CA) in the FLT1 knockdown group had a weaker migratory ability; Figure C shows the phalloidin staining of actin, suggesting that knockdown of FLT1 attenuated the invasion and migration of endometrial cancer cells, but had less effect on the expression and function of actin; Figure D shows the proliferation assay of cck8 cells, suggesting that the proliferative ability of AN3CA was weakened by knockdown of FLT1; Figure E shows the Transwell assay, suggesting that the proliferative ability of AN3CA cells with knockdown of FLT1 had weakened migration ability; Figure F shows plate cloning assay, suggesting that knockdown of FLT1 attenuated the proliferation level of AN3CA cells.


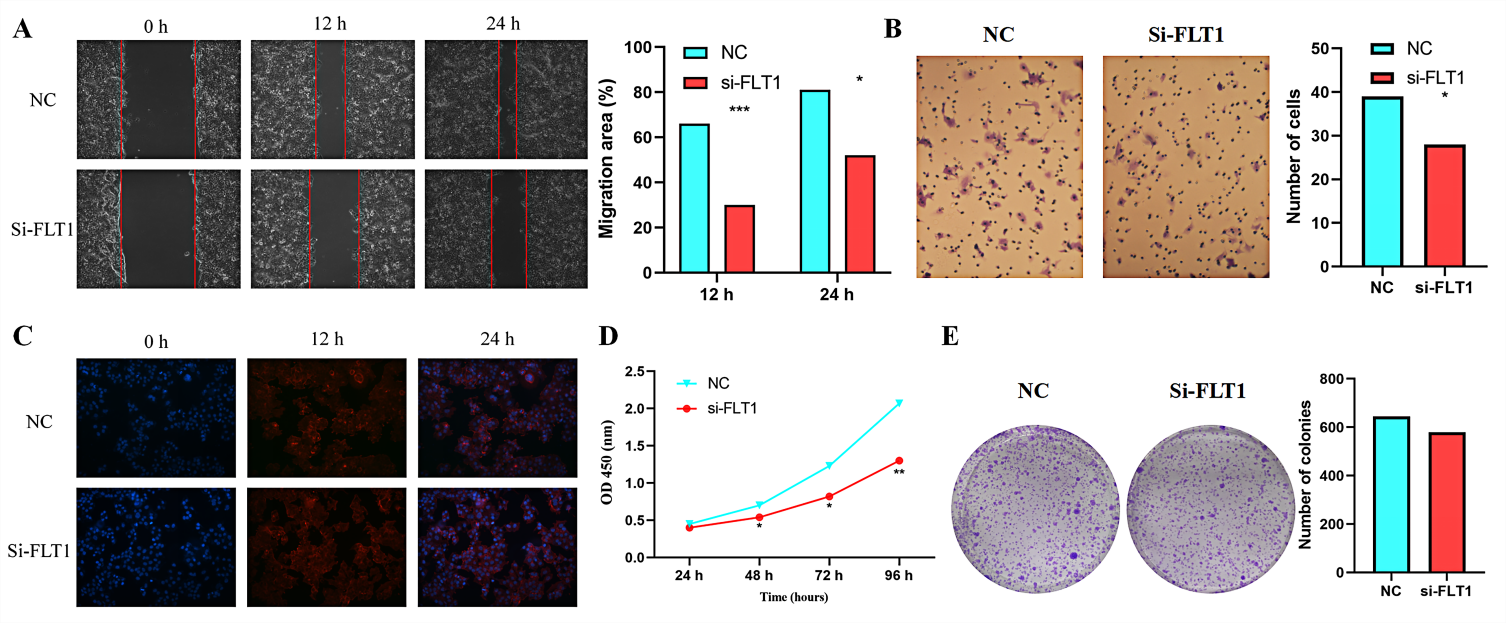


Figure S9  Functional validation of FLT1. Figure A shows the scratch assay, in which endometrial cancer cells (Ishikawa) in the FLT1 knockdown group had weaker migration ability; Figure B shows the Transwell assay, which suggested that AN3CA cells knocked down by FLT1 had weakened invasive and migratory abilities; Figure C shows the phalloidin staining of actin, suggesting that the knockdown of FLT1 would weaken the invasion, and migration of endometrial cancer cells but have less effect on the expression and function of actin; Figure D and E show cck8 and plate cloning experiments, suggesting that knockdown of FLT1 attenuates the proliferation level of Ishikawa cells (but there is no statistically significant difference in plate cloning experiments).

## Tables

Table S1 Scan parameters of each sequence in MRI

| **MR examination** | **TR /TE (ms)** | **Slice thickness (mm)** | **Slice gap (mm)** | **Acquisition acquisition matrix** | **FOV (mm)** |
| --- | --- | --- | --- | --- | --- |
| Axial T_1_WI_C1_ | 500/7 | 6 | 2 | 352 × 192 | 320 × 320 |
| Axial T_1_WI_C2_ | 491/8.8 | 5 | 0.5 | 350 × 200 | 240 × 240 |
| Axial T_1_WI_C3_ | 500/8 | 5 | 1 | 350 × 200 | 240 × 240 |
| Axial T_2_WI_C1_ | 4600/72.5 | 6 | 2 | 320 × 256 | 240 × 240 |
| Axial T_2_WI_C2_ | 3773/128.7 | 5 | 0.5 | 320×250 | 240 × 240 |
| Axial T_2_WI_C3_ | 4600/68 | 3 | 1 | 320×256 | 240 × 240 |
| Axial (FS)-T_2_WI_C1_ | 5000/72.5 | 6 | 2 | 320 × 256 | 240 × 240 |
| Axial (FS)-T_2_WI_C2_ | 5000/75 | 6 | 2 | 320 × 256 | 240 × 240 |
| Axial (FS)-T_2_WI_C3_ | 5000/75 | 6 | 2 | 320 × 256 | 240 × 240 |
| Sagittal T_2_WI_C1_ | 4500/72 | 4 | 1 | 320 × 320 | 280 × 280 |
| Sagittal T_2_WI_C2_ | 3773/128.7 | 5 | 0.5 | 320×250 | 240 × 240 |
| Sagittal T_2_WI_C3_ | 4000/120 | 5 | 1 | 320×250 | 260 × 260 |
| DWI_C1_ | 4000/65 | 4 | 1 | 420 × 420 | 96 × 130 |
| DWI_C2_ | 3950/78.8 | 5 | 0.5 | 100 × 130 | 240 × 240 |
| DWI_C3_ | 3900/78 | 5 | 1 | 96 × 130 | 240 × 240 |

TR, repetition time; TE, echo time; FOV, field of view; FS, fat suppression; _C1_, Center 1; _C2_, Center 2; _C3_, Center 3.

Table S2 Comparison of Baseline Characteristics Between Training and Validation Cohorts

| **Parameters** | **Center 1**  **Training set**  **(n=271)** | **Center 2&3**  **Validation set**  **(n=198)** | **Statistical Test** | **P-value** |
| --- | --- | --- | --- | --- |
| Death | 92 (33.9%) | 66 (33.3%) | χ² | 0.892 |
| Age | 56 [52, 61] | 57 [54, 63] | Mann-Whitney U | 0.053 |
| Height (m) | 1.58±0.36 | 1.55 ± 0.31 | t-test | 0.264 |
| Weight (kg) | 61.6±8.51 | 62.1 ± 8.63 | t-test | 0.495 |
| Body mass index | 25.22±6.02 | 25.81 ± 6.54 | t-test | 0.294 |
| Age of menarche | 15 [14, 16] | 15 [14, 16] | Mann-Whitney U | 0.453 |
| Menopausal | 180 (66.4%) | 141 (71.6%) | χ² | 0.212 |
| Max diameter (cm) | 3.56±1.16 | 3.42 ± 1.14 | t-test | 0.169 |
| CA125（U/mL） | 42.83±6.77 | 45.12 ± 6.58 | t-test | 0.056 |
| Lymph node metastasis | 17 (6.3%) | 8 (4.1%) | Fisher’s exact | 0.258 |
| Deep myometrial infiltration | 113 (41.7%) | 82 (41.4%) | χ² | 0.943 |
| Differentiation |  |  | χ² | 0.71 |
| G1 | 94 (34.7%) | 62 (31.3%) |  |  |
| G2 | 106 (39.1%) | 77 (38.9%) |  |  |
| G3 | 71 (26.2%) | 59 (29.8%) |  |  |
| Clinical types |  |  | χ² | 0.892 |
| type I | 177 (65.3%) | 132 (66.7%) |  |  |
| type II | 94 (34.7%) | 66 (33.3%) |  |  |
| Histological type |  |  | Fisher's exact | 0.421 |
| Endometrial Adenocarcinoma | 223 (82.3%) | 171 (86.4%) |  |  |
| Plasma Cancer | 36 (13.3%) | 18 (9.1%) |  |  |
| Clear Cell Adenocarcinoma | 5 (1.8%) | 3 (1.5%) |  |  |
| Other types | 7 (2.6%) | 6 (3.0%) |  |  |
| FIGO stage |  |  | Trend χ² | 0.542 |
| Ⅰ | 213 (78.6%) | 160 (80.8%) |  |  |
| Ⅱ | 33 (12.2%) | 27 (13.6%) |  |  |
| Ⅲ | 19 (7.0%) | 9 (4.5%) |  |  |
| Ⅳ | 6 (2.2%) | 2 (1.0%) |  |  |

Table S3 Radiomic features retained by LASSO in the tumoral region

| **Feature Name** | **Coefficient** | **Transform** | **Feature Class** |
| --- | --- | --- | --- |
| wavelet_LHH_firstorder_Minimum | -0.0012 | Wavelet (LHH) | Intensity |
| wavelet_LHH_glcm_lmc2 | 0.2085 | Wavelet (LHH) | GLCM |
| wavelet_LHH_firstorder_Skewness | -0.0032 | Wavelet (LHH) | Shape |
| wavelet_HHL_glszm_SizeZoneNonUniformityNormalized | -14.1561 | Wavelet (HHL) | GLSZM |
| wavelet_HHH_glszm_GrayLevelVariance | 0.0021 | Wavelet (HHH) | GLSZM |
| wavelet_HHH_glszm_GrayLevelNonUniformityNormalized | 24.1840 | Wavelet (HHH) | GLSZM |
| wavelet_HHH_glrlm_LowGrayLevelRunEmphasis | 0.0075 | Wavelet (HHH) | GLRLM |
| wavelet_HHH_glrlm_HighGrayLevelRunEmphasis | -38.0921 | Wavelet (HHH) | GLRLM |
| squareroot_glszm_LargeAreaLowGrayLevelEmphasis | -0.148 | Square root | GLSZM |
| square_firstorder_Energy | -0.0015 | Square | Intensity |
| logarithm_glszm_LargeAreaLowGrayLevelEmphasis | 0.0013 | Logarithm | GLSZM |
| lbp_2D_glszm_ZoneEntropy | 0.0067 | LBP 2D | GLSZM |
| lbp_2D_glszm_SizeZoneNonUniformityNormalized | 0.0098 | LBP 2D | GLSZM |
| lbp_2D_glszm_GrayLevelNonUniformity | 2.1425 | LBP 2D | GLSZM |
| gradient_ngtdm_Strength | 0.0855 | Gradient | NGTDM |
| gradient_glcm_MCC | -8.1212 | Gradient | GLCM |

Table S4 Radiomic features retained by LASSO in the peritumoral region

| **Feature Name** | **Coef ficient** | **Transform** | **Feature Class** |
| --- | --- | --- | --- |
| wavelet_LLH_firstorder_Uniformity | 16.0218 | Wavelet (LLH) | Intensity |
| wavelet_HHL_glszm_SizeZoneNonUniformityNormalized | -25.2537 | Wavelet (HHL) | GLSZM |
| wavelet_HHH_glszm_GrayLevelVariance | 0.0035 | Wavelet (HHH) | GLSZM |
| wavelet_HHH_glszm_GrayLevelNonUniformityNormalized | 73.9891 | Wavelet (HHH) | GLSZM |
| squareroot_glcm_DifferenceVariance | 1.3598 | Square root | GLCM |
| square_firstorder_Range | 0.0042 | Square | Intensity |
| original_glcm_lmc1 | -3.4127 | Original | GLCM |
| lbp_2D_glszm_ZoneEntropy | 0.0098 | LBP 2D | GLSZM |
| lbp_2D_glszm_SizeZoneNonUniformityNormalized | 0.0015 | LBP 2D | GLSZM |
| lbp_2D_glszm_GrayLevelNonUniformity | 2.6802 | LBP 2D | GLSZM |
| gradient_gldm_SmallDependenceLowGrayLevelEmphasis | 5.7628 | Gradient | GLDM |
| gradient_glcm_MCC | 0.8932 | Gradient | GLCM |

Table S5 CLEAR checklist

| **Section** | **No** | **Item** | **Yes** | **No** | **n/a** | **Page** |
| --- | --- | --- | --- | --- | --- | --- |
| **Title** |  |  |  |  |  |  |
|  | 1 | Relevant title, specifying the radiomic methodology | 🗹 | ☐ | ☐ | Page 1 |
| **Abstract** |  |  |  |  |  |  |
|  | 2 | Structured summary with relevant information | 🗹 | ☐ | ☐ | Page 1 |
| **Keywords** |  |  |  |  |  |  |
|  | 3 | Relevant keywords for radiomics | 🗹 | ☐ | ☐ | Page 1 |
| **Introduction** |  |  |  |  |  |  |
|  | 4 | Scientific or clinical background | 🗹 | ☐ | ☐ | Page 3 |
|  | 5 | Rationale for using a radiomic approach | 🗹 | ☐ | ☐ | Page 3 |
|  | 6 | Study objective(s) | 🗹 | ☐ | ☐ | Page 3 |
| **Method** |  |  |  |  |  |  |
| ***Study Design*** | 7 | Adherence to guidelines or checklists (e.g., CLEAR checklist) | 🗹 | ☐ | ☐ | Page 4 |
|  | 8 | Ethical details (e.g., approval, consent, data protection) | 🗹 | ☐ | ☐ | Page 4 |
|  | 9 | Sample size calculation | 🗹 | ☐ | ☐ | Page 4 |
|  | 10 | Study nature (e.g., retrospective, prospective) | 🗹 | ☐ | ☐ | Page 4 |
|  | 11 | Eligibility criteria | 🗹 | ☐ | ☐ | Page 4 and supplementary material page 1 |
|  | 12 | Flowchart for technical pipeline | 🗹 | ☐ | ☐ | Page 5 |
| ***Data*** | 13 | Data source (e.g., private, public) | 🗹 | ☐ | ☐ | Page 4 |
|  | 14 | Data overlap | 🗹 | ☐ | ☐ | Page 4 |
|  | 15 | Data split methodology | 🗹 | ☐ | ☐ | Page 5 |
|  | 16 | Imaging protocol (i.e., image acquisition and processing) | 🗹 | ☐ | ☐ | Page 6 and supplementary material page 2 |
|  | 17 | Definition of non-radiomic predictor variables | 🗹 | ☐ | ☐ | Page 4 |
|  | 18 | Definition of the reference standard (i.e., outcome variable) | 🗹 | ☐ | ☐ | Page 4 |
| ***Segmentation*** | 19 | Segmentation strategy | 🗹 | ☐ | ☐ | Page 6 and supplementary material page 3 |
|  | 20 | Details of operators performing segmentation | 🗹 | ☐ | ☐ | Page 5 and supplementary material page 3 |
| ***Pre-processing*** | 21 | Image pre-processing details | 🗹 | ☐ | ☐ | Page 6 and supplementary material page 3 |
|  | 22 | Resampling method and its parameters | 🗹 | ☐ | ☐ | Page 4 |
|  | 23 | Discretization method and its parameters | 🗹 | ☐ | ☐ | Page 6 and Page 8 |
|  | 24 | Image types (e.g., original, filtered, transformed) | 🗹 | ☐ | ☐ | Page 6 |
| ***Feature extraction*** | 25 | Feature extraction method | 🗹 | ☐ | ☐ | Page 6 and supplementary material page 4 |
|  | 26 | Feature classes | 🗹 | ☐ | ☐ | Page 7 and supplementary material page 4 |
|  | 27 | Number of features | 🗹 | ☐ | ☐ | Page 6 and supplementary material page 4 |
|  | 28 | Default configuration statement for remaining parameters | 🗹 | ☐ | ☐ | Page 6 and supplementary material page 4 |
| ***Data preparation*** | 29 | Handling of missing data | 🗹 | ☐ | ☐ | Page 4 and supplementary material page 5 |
|  | 30 | Details of class imbalance | 🗹 | ☐ | ☐ | Page 4 and supplementary material page 5 |
|  | 31 | Details of segmentation reliability analysis | 🗹 | ☐ | ☐ | Supplementary material page 3 |
|  | 32 | Feature scaling details (e.g., normalization, standardization) | 🗹 | ☐ | ☐ | Page 6 and supplementary material page 3 |
|  | 33 | Dimension reduction details | 🗹 | ☐ | ☐ | Page 6 and supplementary material page 3 |
| ***Modeling*** | 34 | Algorithm details | 🗹 | ☐ | ☐ | Page 7 |
|  | 35 | Training and tuning details | 🗹 | ☐ | ☐ | Page 7 |
|  | 36 | Handling of confounders | 🗹 | ☐ | ☐ | Page 7 |
|  | 37 | Model selection strategy | 🗹 | ☐ | ☐ | Page 7 and supplementary material page 6 |
| ***Evaluation*** | 38 | Testing technique (e.g., internal, external) | 🗹 | ☐ | ☐ | Page 4 |
|  | 39 | Performance metrics and rationale for choosing | 🗹 | ☐ | ☐ | Page 7 |
|  | 40 | Uncertainty evaluation and measures (e.g., confidence intervals) | 🗹 | ☐ | ☐ | Page 7 |
|  | 41 | Statistical performance comparison (e.g., DeLong’s test) | 🗹 | ☐ | ☐ | Page 8 |
|  | 42 | Comparison with non-radiomic and combined methods | 🗹 | ☐ | ☐ | Page 7 |
|  | 43 | Interpretability and explainability methods | 🗹 | ☐ | ☐ | Page 8 |
| **Results** |  |  |  |  |  |  |
|  | 44 | Baseline demographic and clinical characteristics | 🗹 | ☐ | ☐ | Page 9 |
|  | 45 | Flowchart for eligibility criteria | 🗹 | ☐ | ☐ | Page 5 |
|  | 46 | Feature statistics (e.g., reproducibility, feature selection) | 🗹 | ☐ | ☐ | Page 10 |
|  | 47 | Model performance evaluation | 🗹 | ☐ | ☐ | Page 11 |
|  | 48 | Comparison with non-radiomic and combined approaches | 🗹 | ☐ | ☐ | Page 13 |
| **Discussion** |  |  |  |  |  |  |
|  | 49 | Overview of important findings | 🗹 | ☐ | ☐ | Page 17 |
|  | 50 | Previous works with differences from the current study | 🗹 | ☐ | ☐ | Page 18 |
|  | 51 | Practical implications | 🗹 | ☐ | ☐ | Page 21 |
|  | 52 | Strengths and limitations (e.g., bias and generalizability issues) | 🗹 | ☐ | ☐ | Page 21 |
| **Open Science** |  |  |  |  |  |  |
| ***Data availability*** | 53 | Sharing images along with segmentation data [n/e] | 🗹 | ☐ | ☐ | Page 7 and supplementary material page 4 |
|  | 54 | Sharing radiomic feature data | 🗹 | ☐ | ☐ | Supplementary material page 4 |
| ***Code availability*** | 55 | Sharing pre-processing scripts or settings | 🗹 | ☐ | ☐ | Page 7 and supplementary material page 4 |
|  | 56 | Sharing source code for modeling | 🗹 | ☐ | ☐ | Supplementary material page 4 |
| ***Model availability*** | 57 | Sharing final model files | 🗹 | ☐ | ☐ | Supplementary material page 7 |
|  | 58 | Sharing a ready-to-use system [n/e] | ☐ | 🗹 | ☐ |  |

Table S6 The radiomics quality score (RQS) tool

| **No** | **Criteria** | **Points** |
| --- | --- | --- |
| 1 | **Image protocol quality** - well-documented image protocols (for example, contrast, slice thickness, energy, etc.) and/or usage of public image protocols allow reproducibility/replicability | +1 (public protocol is used)  +1 (protocols are well-documented) |
| 2 | **Multiple segmentations** - possible actions are: segmentation by different physicians/algorithms/software, perturbing segmentations by (random) noise, segmentation at different breathing cycles. Analyse feature robustness to segmentation variabilities | +1 (segmentation by different doctors) |
| 3 | **Phantom study on all scanners** - detect inter-scanner differences and vendor-dependent features. Analyse feature robustness to these sources of variability | +1 (analyse feature robustness to these sources) |
| 4 | **I****maging at multiple time points** - collect images of individuals at additional time points. Analyse feature robustness to temporal variabilities (for example, organ movement, organ expansion/ shrinkage) |  |
| 5 | **Feature reduction or adjustment for multiple testing** - decreases the risk of overfitting. Overfitting is inevitable if the number of features exceeds the number of samples. Consider feature robustness when selecting features | +3 (measure is implemented) |
| 6 | **Multivariable analysis with non-radiomics features** (for example, EGFR mutation) - is expected to provide a more holistic model. Permits correlating/inferencing between radiomics and non-radiomics features | +1 (multivariable analysis with non-radiomics features) |
| 7 | **Detect and discuss biological correlates** - demonstration of phenotypic differences (possibly associated with underlying gene–protein expression patterns) deepens understanding of radiomics and biology | +1 (detect and discuss biological correlates) |
| 8 | **Cut-off analyses** - determine risk groups by either the median, a previously published cut-off or report a continuous risk variable. Reduces the risk of reporting overly optimistic results | +1 (cut-off analyses) |
| 9 | **Discrimination statistics** - report discrimination statistics (for example, C-statistic, ROC curve, AUC) and their statistical significance (for example, p-values, confidence intervals). One can also apply resampling method (for example, bootstrapping, cross-validation) | +1 (discrimination statistic is reported)  +1 (resampling method technique is applied) |
| 10 | **Calibration statistics** - report calibration statistics (for example, Calibration-in-the-large/slope, calibration plots) and their statistical significance (for example, P-values, confidence intervals). One can also apply resampling method (for example, bootstrapping, cross-validation) | +1 (calibration statistic is reported)  +1 (resampling method technique is applied) |
| 11 | **Prospective study registered in a trial database** - provides the highest level of evidence supporting the clinical validity and usefulness of the radiomics biomarker | +7 (Prospective study) |
| 12 | **Validation** - the validation is performed without retraining and without adaptation of the cut-off value, provides crucial information with regard to credible clinical performance | +5 (Three centers) |
| 13 | **Comparison to ‘gold standard’** - assess the extent to which the model agrees with/is superior to the current ‘gold standard’ method (for example, TNM-staging for survival prediction). This comparison shows the added value of radiomics | +2 (TNM stage) |
| 14 | **Potential clinical utility** - report on the current and potential application of the model in a clinical setting (for example, decision curve analysis). | +2 (decision curve analysis) |
| 15 | **Cost-effectiveness analysis** - report on the cost-effectiveness of the clinical application (for example, QALYs generated) | +1 (clinical impact curves) |
| 16 | **Open science and data** - make code and data publicly available. Open science facilitates knowledge transfer and reproducibility of the study |  |
|  | Total points (36 = 100%) | 31 (86.1%) |

AUC=area under the curve; EGFR=epidermal growth factor receptor.

Table S7 The radiomics quality score (METRICS) tool

| **Items/Conditions** | **Definitions** | **Weights** | **Yes/No** |
| --- | --- | --- | --- |
| Study Design |  |  |  |
| Item#1 | Adherence to radiomics and/or machine learning-specific checklists or guidelines | 0.0368 | yes |
| Item#2 | Eligibility criteria that describe a representative study population | 0.0735 | yes |
| Item#3 | High-quality reference standard with a clear definition | 0.0919 | yes |
| Imaging Data |  |  |  |
| Item#4 | Multi-center | 0.0438 | yes |
| Item#5 | Clinical translatability of the imaging data source for radiomics analysis | 0.0292 | yes |
| Item#6 | Imaging protocol with acquisition parameters | 0.0438 | yes |
| Item#7 | The interval between imaging used and reference standard | 0.0292 | yes |
| SegmentationC |  |  |  |
| Condition#1 | Does the study include segmentation? |  | yes |
| Condition#2 | Does the study include fully automated segmentation? |  | no |
| Item#8 | Transparent description of segmentation methodology | 0.0337 | no |
| Item#9 | Formal evaluation of fully automated segmentationC | 0.0225 | n/a |
| Item#10 | Test set segmentation masks produced by a single reader or automated tool | 0.0112 | no |
| Image Processing and Feature Extraction |  |  |  |
| Condition#3 | Does the study include hand-crafted feature extraction? |  | yes |
| Item#11 | Appropriate use of image preprocessing techniques with transparent description | 0.0622 | yes |
| Item#12 | Use of standardized feature extraction softwareC | 0.0311 | yes |
| Item#13 | Transparent reporting of feature extraction parameters, otherwise providing a default configuration statement | 0.0415 | yes |
| Feature Processing |  |  |  |
| Condition#4 | Does the study include tabular data? |  | yes |
| Condition#5 | Does the study include end-to-end deep learning? |  | yes |
| Item#14 | Removal of non-robust featuresC | 0.0200 | yes |
| Item#15 | Removal of redundant featuresC | 0.0200 | yes |
| Item#16 | Appropriateness of dimensionality compared to data sizeC | 0.0300 | yes |
| Item#17 | Robustness assessment of end-to-end deep learning pipelinesC | 0.0200 | yes |
| Preparation for Modeling |  |  |  |
| Item#18 | Proper data partitioning process | 0.0599 | yes |
| Item#19 | Handling of confounding factors | 0.0300 | yes |
| Metrics and Comparison |  |  |  |
| Item#20 | Use of appropriate performance evaluation metrics for task | 0.0352 | yes |
| Item#21 | Consideration of uncertainty | 0.0234 | yes |
| Item#22 | Calibration assessment | 0.0176 | yes |
| Item#23 | Use of uni-parametric imaging or proof of its inferiority | 0.0117 | yes |
| Item#24 | Comparison with a non-radiomic approach or proof of added clinical value | 0.0293 | yes |
| Item#25 | Comparison with simple or classical statistical models | 0.0176 | yes |
| Testing |  |  |  |
| Item#26 | Internal testing | 0.0375 | yes |
| Item#27 | External testing | 0.0749 | yes |
| Open Science |  |  |  |
| Item#28 | Data availability | 0.0075 | no |
| Item#29 | Code availability | 0.0075 | no |
| Item#30 | Model availability | 0.0075 | no |
| Total METRICS score: |  |  | 93.1% |
| Quality category: |  |  | Excellent |

Table S8 DeLong's Test Comparison Between XGBoost and Other Models

| **Comparative Model** | **Time Point**  **(Dataset&Year)** | **XGBoost AUC** | **Comparative Model AUC** | **P-Value** | **Significance**  **(P<0.05)** |
| --- | --- | --- | --- | --- | --- |
| aorsf | Validation set, 1-year | 0.862 | 0.796 | 0.018 | Yes |
|  | Validation set, 3-year | 0.885 | 0.770 | <0.001 | Yes |
|  | Validation set, 5-year | 0.870 | 0.759 | <0.001 | Yes |
|  | Test set 1, 1-year | 0.823 | 0.755 | 0.023 | Yes |
|  | Test set 1, 3-year | 0.869 | 0.741 | <0.001 | Yes |
|  | Test set 1, 5-year | 0.849 | 0.760 | 0.007 | Yes |
|  | Test set 2, 1-year | 0.850 | 0.716 | <0.001 | Yes |
|  | Test set 2, 3-year | 0.731 | 0.702 | 0.395 | No |
|  | Test set 2, 5-year | 0.800 | 0.741 | 0.066 | No |
|  | Total Significant Time Points | - | - | - | 7/9 (77.8%) |
| Bart | Validation set, 1-year | 0.862 | 0.815 | 0.082 | No |
|  | Validation set, 3-year | 0.885 | 0.780 | <0.001 | Yes |
|  | Validation set, 5-year | 0.870 | 0.807 | 0.030 | Yes |
|  | Test set 1, 1-year | 0.823 | 0.752 | 0.014 | Yes |
|  | Test set 1, 3-year | 0.869 | 0.694 | <0.001 | Yes |
|  | Test set 1, 5-year | 0.849 | 0.790 | 0.057 | No |
|  | Test set 2, 1-year | 0.850 | 0.760 | 0.008 | Yes |
|  | Test set 2, 3-year | 0.731 | 0.678 | 0.131 | No |
|  | Test set 2, 5-year | 0.800 | 0.722 | 0.018 | Yes |
|  | Total Significant Time Points | - | - | - | 6/9 (66.7%) |
| Deephit | Validation set, 1-year | 0.862 | 0.734 | <0.001 | Yes |
|  | Validation set, 3-year | 0.885 | 0.660 | <0.001 | Yes |
|  | Validation set, 5-year | 0.870 | 0.689 | <0.001 | Yes |
|  | Test set 1, 1-year | 0.823 | 0.680 | <0.001 | Yes |
|  | Test set 1, 3-year | 0.869 | 0.761 | 0.002 | Yes |
|  | Test set 1, 5-year | 0.849 | 0.697 | <0.001 | Yes |
|  | Test set 2, 1-year | 0.850 | 0.705 | <0.001 | Yes |
|  | Test set 2, 3-year | 0.731 | 0.695 | 0.317 | No |
|  | Test set 2, 5-year | 0.800 | 0.714 | 0.014 | Yes |
|  | Total Significant Time Points | - | - | - | 8/9 (88.9%) |
| Deepsurv | Validation set, 1-year | 0.862 | 0.842 | 0.490 | No |
|  | Validation set, 3-year | 0.885 | 0.865 | 0.503 | No |
|  | Validation set, 5-year | 0.870 | 0.826 | 0.155 | No |
|  | Test set 1, 1-year | 0.823 | 0.826 | 0.928 | No |
|  | Test set 1, 3-year | 0.869 | 0.811 | 0.087 | No |
|  | Test set 1, 5-year | 0.849 | 0.760 | 0.011 | Yes |
|  | Test set 2, 1-year | 0.850 | 0.772 | 0.030 | Yes |
|  | Test set 2, 3-year | 0.731 | 0.655 | 0.040 | Yes |
|  | Test set 2, 5-year | 0.800 | 0.705 | 0.008 | Yes |
|  | Total Significant Time Points | - | - | - | 4/9 (44.4%) |
| Dnnsurv | Validation set, 1-year | 0.862 | 0.697 | <0.001 | Yes |
|  | Validation set, 3-year | 0.885 | 0.767 | <0.001 | Yes |
|  | Validation set, 5-year | 0.870 | 0.829 | 0.200 | No |
|  | Test set 1, 1-year | 0.823 | 0.777 | 0.164 | No |
|  | Test set 1, 3-year | 0.869 | 0.676 | <0.001 | Yes |
|  | Test set 1, 5-year | 0.849 | 0.821 | 0.424 | No |
|  | Test set 2, 1-year | 0.850 | 0.825 | 0.496 | No |
|  | Test set 2, 3-year | 0.731 | 0.692 | 0.303 | No |
|  | Test set 2, 5-year | 0.800 | 0.808 | 0.825 | No |
|  | Total Significant Time Points | - | - | - | 3/9 (33.3%) |
| GBM | Validation set, 1-year | 0.862 | 0.788 | 0.013 | Yes |
|  | Validation set, 3-year | 0.885 | 0.742 | <0.001 | Yes |
|  | Validation set, 5-year | 0.870 | 0.720 | <0.001 | Yes |
|  | Test set 1, 1-year | 0.823 | 0.633 | <0.001 | Yes |
|  | Test set 1, 3-year | 0.869 | 0.750 | 0.001 | Yes |
|  | Test set 1, 5-year | 0.849 | 0.736 | 0.002 | Yes |
|  | Test set 2, 1-year | 0.850 | 0.716 | <0.001 | Yes |
|  | Test set 2, 3-year | 0.731 | 0.692 | 0.317 | No |
|  | Test set 2, 5-year | 0.800 | 0.729 | 0.061 | No |
|  | Total Significant Time Points | - | - | - | 7/9 (77.8%) |
| Glmnet | Validation set, 1-year | 0.862 | 0.863 | 0.976 | No |
|  | Validation set, 3-year | 0.885 | 0.851 | 0.271 | No |
|  | Validation set, 5-year | 0.870 | 0.790 | 0.012 | Yes |
|  | Test set 1, 1-year | 0.823 | 0.809 | 0.674 | No |
|  | Test set 1, 3-year | 0.869 | 0.832 | 0.289 | No |
|  | Test set 1, 5-year | 0.849 | 0.767 | 0.023 | Yes |
|  | Test set 2, 1-year | 0.850 | 0.796 | 0.155 | No |
|  | Test set 2, 3-year | 0.731 | 0.806 | 0.055 | No |
|  | Test set 2, 5-year | 0.800 | 0.731 | 0.069 | No |
|  | Total Significant Time Points | - | - | - | 2/9 (22.2%) |
| RSF | Validation set, 1-year | 0.862 | 0.836 | 0.368 | No |
|  | Validation set, 3-year | 0.885 | 0.815 | 0.024 | Yes |
|  | Validation set, 5-year | 0.870 | 0.801 | 0.031 | Yes |
|  | Test set 1, 1-year | 0.823 | 0.798 | 0.435 | No |
|  | Test set 1, 3-year | 0.869 | 0.823 | 0.177 | No |
|  | Test set 1, 5-year | 0.849 | 0.762 | 0.013 | Yes |
|  | Test set 2, 1-year | 0.850 | 0.718 | <0.001 | Yes |
|  | Test set 2, 3-year | 0.731 | 0.733 | 0.960 | No |
|  | Test set 2, 5-year | 0.800 | 0.796 | 0.912 | No |
|  | Total Significant Time Points | - | - | - | 4/9 (44.4%) |
| SVM | Validation set, 1-year | 0.862 | 0.778 | 0.004 | Yes |
|  | Validation set, 3-year | 0.885 | 0.815 | 0.024 | Yes |
|  | Validation set, 5-year | 0.870 | 0.801 | 0.031 | Yes |
|  | Test set 1, 1-year | 0.823 | 0.700 | <0.001 | Yes |
|  | Test set 1, 3-year | 0.869 | 0.823 | 0.190 | No |
|  | Test set 1, 5-year | 0.849 | 0.762 | 0.015 | Yes |
|  | Test set 2, 1-year | 0.850 | 0.650 | <0.001 | Yes |
|  | Test set 2, 3-year | 0.731 | 0.733 | 0.960 | No |
|  | Test set 2, 5-year | 0.800 | 0.796 | 0.912 | No |
|  | Total Significant Time Points | - | - | - | 6/9 (66.7%) |

Table S9 Incremental value of Radscore for clinical metrics

| **Year** | **IDI** | ***P*-Value** | **NRI** | ***P*-Value** | **MIR Score** | ***P*-Value** |
| --- | --- | --- | --- | --- | --- | --- |
| 1-year | 0.318 | 0.013 | 0.049 | 0.018 | 0.523 | 0.011 |
| 3-year | 0.140 | 0.001 | 0.271 | 0.022 | 0.201 | 0.005 |
| 5-year | 0.112 | 0.003 | 0.234 | 0.027 | 0.270 | 0.047 |

IDI, integrated discrimination improvement; NRI, net reclssification improvement; MIR, median improvement risk

Table S10 Area under the ROC curve and 95% confidence intervals

| **Group** | | **Year** | **Radiomics Model** | | **Nomogram** | | **Multi-omics Model** | |
| --- | --- | --- | --- | --- | --- | --- | --- | --- |
|  |  |  | AUC | 95%CI | AUC | 95%CI | AUC | 95%CI |
| Tumor | Training set | 1-year | 0.900 | 0.852-0.948 |  |  |  |  |
|  |  | 3-year | 0.916 | 0.875-0.957 |  |  |  |  |
|  |  | 5-year | 0.873 | 0.820-0.926 |  |  |  |  |
|  | Validation set | 1-year | 0.849 | 0.795-0.903 |  |  |  |  |
|  |  | 3-year | 0.879 | 0.830-0.928 |  |  |  |  |
|  |  | 5-year | 0.829 | 0.770-0.888 |  |  |  |  |
|  | Test set 1 | 1-year | 0.841 | 0.780-0.902 |  |  |  |  |
|  |  | 3-year | 0.810 | 0.740-0.880 |  |  |  |  |
|  |  | 5-year | 0.711 | 0.630-0.792 |  |  |  |  |
|  | Test set 2 | 1-year | 0.811 | 0.740-0.882 |  |  |  |  |
|  |  | 3-year | 0.749 | 0.665-0.833 |  |  |  |  |
|  |  | 5-year | 0.622 | 0.530-0.714 |  |  |  |  |
| Peritumor | Training set | 1-year | 0.928 | 0.890-0.966 |  |  |  |  |
|  |  | 3-year | 0.812 | 0.750-0.874 |  |  |  |  |
|  |  | 5-year | 0.839 | 0.780-0.898 |  |  |  |  |
|  | Validation set | 1-year | 0.863 | 0.810-0.916 |  |  |  |  |
|  |  | 3-year | 0.873 | 0.820-0.926 |  |  |  |  |
|  |  | 5-year | 0.793 | 0.730-0.856 |  |  |  |  |
|  | Test set 1 | 1-year | 0.765 | 0.690-0.840 |  |  |  |  |
|  |  | 3-year | 0.781 | 0.710-0.852 |  |  |  |  |
|  |  | 5-year | 0.831 | 0.770-0.892 |  |  |  |  |
|  | Test set 2 | 1-year | 0.785 | 0.710-0.860 |  |  |  |  |
|  |  | 3-year | 0.756 | 0.680-0.832 |  |  |  |  |
|  |  | 5-year | 0.680 | 0.595-0.765 |  |  |  |  |
| Combine | Training set | 1-year | 0.916 | 0.875-0.957 | 0.940 | 0.905-0.975 |  |  |
|  |  | 3-year | 0.913 | 0.870-0.956 | 0.992 | 0.980-1.000 |  |  |
|  |  | 5-year | 0.899 | 0.850-0.948 | 0.927 | 0.890-0.964 |  |  |
|  | Validation set | 1-year | 0.862 | 0.810-0.914 | 0.884 | 0.835-0.933 |  |  |
|  |  | 3-year | 0.885 | 0.835-0.935 | 0.915 | 0.870-0.960 |  |  |
|  |  | 5-year | 0.870 | 0.820-0.920 | 0.909 | 0.865-0.953 |  |  |
|  | Test set 1 | 1-year | 0.823 | 0.760-0.886 | 0.872 | 0.815-0.929 |  |  |
|  |  | 3-year | 0.869 | 0.810-0.928 | 0.881 | 0.825-0.937 |  |  |
|  |  | 5-year | 0.849 | 0.790-0.908 | 0.794 | 0.730-0.858 |  |  |
|  | Test set 2 | 1-year | 0.850 | 0.790-0.910 |  |  | 0.989 | 0.970-1.000 |
|  |  | 3-year | 0.731 | 0.650-0.812 |  |  | 0.996 | 0.990-1.000 |
|  |  | 5-year | 0.800 | 0.730-0.870 |  |  | 1.000 | 1.000-1.000 |

Table S11 Brier scores of calibration curves for each model

| **Brier score** | **Radiomics Model** | | | **Nomogram Model** | | | **Multi-omics Model** | | |
| --- | --- | --- | --- | --- | --- | --- | --- | --- | --- |
|  | 1-year | 3-year | 5-year | 1-year | 3-year | 5-year | 1-year | 3-year | 5-year |
| Training set | 0.042 | 0.013 | 0.039 | 0.168 | 0.143 | 0.081 |  |  |  |
| Validation set | 0.083 | 0.024 | 0.011 | 0.197 | 0.015 | 0.038 |  |  |  |
| Test set1 | 0.137 | 0.008 | 0.010 | 0.086 | 0.012 | 0.047 |  |  |  |
| Test set2 | 0.148 | 0.056 | 0.025 |  |  |  | 0.219 | 0.011 | 0.041 |

Table S12 Interclass correlation coefficient of IVIM-DWI and DCE-MRI parameters

| **Group** | **Parameters** | **ICC** | **95% CI** |
| --- | --- | --- | --- |
| IVIM-DWI | Apparent Diffusion Coefficient | 0.920 | 0.860–0.960 |
|  | True Diffusion Coefficient | 0.900 | 0.830–0.950 |
|  | Perfusion Fraction | 0.880 | 0.800–0.940 |
|  | Pseudodiffusion Coefficient | 0.850 | 0.760–0.920 |
| DCE-MRI | Transfer Constant | 0.910 | 0.850–0.960 |
|  | Rate Constant | 0.890 | 0.820–0.950 |
|  | Plasma Volume Fraction | 0.870 | 0.790–0.930 |
|  | Extravascular Extracellular Space Volume Fraction | 0.890 | 0.820–0.950 |
